# Association of Habitual Alcohol Intake With Risk of Cardiovascular Disease

**DOI:** 10.1001/jamanetworkopen.2022.3849

**Published:** 2022-03-25

**Authors:** Kiran J. Biddinger, Connor A. Emdin, Mary E. Haas, Minxian Wang, George Hindy, Patrick T. Ellinor, Sekar Kathiresan, Amit V. Khera, Krishna G. Aragam

**Affiliations:** 1 Program in Medical and Population Genetics, Broad Institute of MIT and Harvard, Cambridge, Massachusetts; 2 Center for Genomic Medicine, Massachusetts General Hospital, Harvard Medical School, Boston; 3 Cardiovascular Research Center, Massachusetts General Hospital, Harvard Medical School, Boston; 4 now with Regeneron Pharmaceuticals, Tarrytown, New York; 5 Qatar University, Doha, Qatar; 6 Verve Therapeutics, Cambridge, Massachusetts

## Abstract

**Importance:**

Observational studies have consistently proposed cardiovascular benefits associated with light alcohol consumption, while recent genetic analyses (ie, mendelian randomization studies) have suggested a possible causal link between alcohol intake and increased risk of cardiovascular disease. However, traditional approaches to genetic epidemiology assume a linear association and thus have not fully evaluated dose-response estimates of risk across different levels of alcohol intake.

**Objectives:**

To assess the association of habitual alcohol intake with cardiovascular disease risk and to evaluate the direction and relative magnitude of cardiovascular risk associated with different amounts of alcohol consumption.

**Design, Setting, and Participants:**

This cohort study used the UK Biobank (2006-2010, follow-up until 2016) to examine confounding in epidemiologic associations between alcohol intake and cardiovascular diseases. Using both traditional (ie, linear) and nonlinear mendelian randomization, potential associations between alcohol consumption and cardiovascular diseases (eg, hypertension and coronary artery disease) as well as corresponding association shapes were assessed. Data analysis was conducted from July 2019 to January 2022.

**Exposures:**

Genetic predisposition to alcohol intake.

**Main Outcomes and Measures:**

The association between alcohol consumption and cardiovascular diseases, including hypertension, coronary artery disease, myocardial infarction, stroke, heart failure, and atrial fibrillation.

**Results:**

This study included 371 463 participants (mean [SD] age, 57.0 [7.9] years; 172 400 [46%] men), who consumed a mean (SD) 9.2 (10.6) standard drinks per week. Overall, 121 708 participants (33%) had hypertension. Light to moderate alcohol consumption was associated with healthier lifestyle factors, adjustment for which attenuated the cardioprotective epidemiologic associations with modest intake. In linear mendelian randomization analyses, a 1-SD increase in genetically predicted alcohol consumption was associated with 1.3-fold (95% CI, 1.2-1.4) higher risk of hypertension (*P* < .001) and 1.4-fold (95% CI, 1.1-1.8) higher risk of coronary artery disease (*P* = .006). Nonlinear mendelian randomization analyses suggested nonlinear associations between alcohol consumption and both hypertension and coronary artery disease: light alcohol intake was associated with minimal increases in cardiovascular risk, whereas heavier consumption was associated with exponential increases in risk of both clinical and subclinical cardiovascular disease.

**Conclusions and Relevance:**

In this cohort study, coincident, favorable lifestyle factors attenuated the observational benefits of modest alcohol intake. Genetic epidemiology suggested that alcohol consumption of all amounts was associated with increased cardiovascular risk, but marked risk differences exist across levels of intake, including those accepted by current national guidelines.

## Key Points

**Question:**

What is the risk of cardiovascular disease associated with different amounts of habitual alcohol consumption?

**Findings:**

In this cohort study of 371 463 individuals, genetic evidence supported a nonlinear, consistently risk-increasing association between all amounts of alcohol consumption and both hypertension and coronary artery disease, with modest increases in risk with light alcohol intake and exponentially greater risk increases at higher levels of consumption.

**Meaning:**

In this study, alcohol consumption at all levels was associated with increased risk of cardiovascular disease, but clinical and public health guidance around habitual alcohol use should account for the considerable differences in cardiovascular risk across different levels of alcohol consumption, even those within current guideline-recommended limits.

## Teaser

This cohort study assesses the association between habitual alcohol intake and cardiovascular disease risk and evaluates the direction and relative magnitude of cardiovascular risk associated with different amounts of alcohol consumption.

## Introduction

Controversy has surrounded the association between alcohol intake and cardiovascular disease (CVD), which remains the leading global cause of death.^1,2,3^ Observational studies have repeatedly demonstrated a lower risk of CVD with light to moderate alcohol intake compared with either abstinence or heavy consumption, suggesting J- or U-shaped epidemiologic associations.^4,5,6,7,8,9^ However, the observed cardiac benefits of alcohol have been hypothesized to be the product of residual confounding because of favorable lifestyle, socioeconomic, and behavioral factors that tend to coincide with modest alcohol intake.^10,11^

Efforts to address this complex association through a randomized clinical trial have been met with logistical and ethical challenges, culminating in the discontinuation of a trial of modest alcohol consumption led by the National Institutes of Health.^12^ In the absence of a randomized trial, a technique using human genetic data (ie, mendelian randomization [MR]) has enabled assessment for potential causal associations by leveraging naturally occurring genetic variants as unbiased proxies for an exposure (ie, alcohol intake).^13^ Given the random allocation of genetic variants at conception, MR obviates concerns of confounding and reverse causality, 2 key limitations of observational epidemiology.

Prior genetic analyses using an MR approach have provided evidence to suggest a causal link between alcohol consumption and increased risk of cardiovascular disease.^14,15,16,17^ However, traditional methods in MR often presume linearity and may therefore be limited in their assessments of relative risks across levels of alcohol intake, which have the potential to inform public health decisions around quantitative risk thresholds.^18,19^ Indeed, the paucity of quantitative data focused on the consequences of moderate alcohol consumption (ie, 1 vs 2 drinks per day) has contributed to variable public health recommendations on low-risk drinking around the world and to the US Department of Agriculture (USDA) maintaining its long-standing, sex-specific recommendations of fewer than 15 drinks per week for men and fewer than 8 drinks per week for women in the 2020-2025 Dietary Guidelines for Americans.^8,20,21,22^ More recently, emerging techniques in nonlinear MR (NLMR) have used large-scale, individual-level genetic data to enable simultaneous assessments of potential causality and association shape.^18,19,23,24,25,26^

To further explore the association between alcohol intake and CVD, we first reexamined how coincident lifestyle and behavioral factors affect the well-established J-shaped observational associations. Next, through traditional MR and NLMR approaches, we evaluated the association of alcohol consumption with CVD, with an emphasis on better understanding relative differences in risk across levels of intake.

## Methods

### Study Population

The primary study population comprised 371 463 unrelated individuals of European genetic ancestry from the UK Biobank (eMethods 1-4 in the Supplement). Informed consent was obtained for all UK Biobank study participants, and analysis was approved by the Mass General Brigham Health Care institutional review board. Select analyses were replicated in 30 716 individuals from the Mass General Brigham Biobank (eMethods 5 in the Supplement). This study followed the Strengthening the Reporting of Observational Studies in Epidemiology (STROBE) reporting guideline.

### Genetic Instruments for Alcohol Consumption

Genetic instruments for habitual alcohol consumption were constructed using single-nucleotide variants (SNVs) associated with alcohol use disorder (AUD; 9 SNVs) and the Alcohol Use Disorder Identification Test–Consumption (AUDIT-C) questionnaire (13 SNVs), as identified in a recent genomewide association study (eTable 1 in the Supplement).^27^ To derive appropriate and specific genetic proxies for habitual alcohol consumption, we first removed SNVs independently associated with relevant risk factors (smoking, body mass index [BMI], physical activity, vegetable intake, red meat intake, overall health rating, C-reactive protein level, and total cholesterol level) to create refined, nonpleiotropic genetic instruments: 4 SNVs were removed from the AUD instrument to create the AUD-Restricted (AUD-R) instrument (5 remaining SNVs), and 3 SNVs were removed from the AUDIT-C instrument to create the AUDIT-C–Restricted (AUDIT-C-R) instrument (10 remaining SNVs). We then assessed all 4 instruments for the 3 assumptions of MR: (1) association with exposure (alcohol phenotypes [eMethods 3 in the Supplement]), with an *F* statistic greater than 10 signifying a strong genetic instrument^28^; (2) no association with confounders; and (3) no direct association with the outcome. We standardized each instrument to a 1–drink per day increase in consumption using empirical, UK Biobank estimates, to arrive at population-specific genetic proxies for habitual alcohol consumption. The AUD-R genetic score was designated the primary instrument owing to residual pleiotropy detected within the AUDIT-C-R instrument (eMethods 6 in the Supplement).

### Study End Points

We focused on 6 CVD phenotypes: hypertension, coronary artery disease (CAD), myocardial infarction (MI), stroke, heart failure, and atrial fibrillation (eTable 2 in the Supplement). In addition, 10 continuous variables were examined: systolic blood pressure (SBP), diastolic blood pressure (DBP), low-density lipoprotein (LDL) cholesterol level, high-density lipoprotein (HDL) cholesterol level, total cholesterol level, triglyceride level, apolipoproteins A and B levels, γ-glutamyl transferase level, and C-reactive protein level.

### Statistical Analysis

Drinking groups were defined as abstainers (0 drinks/wk), light (>0-8.4 drinks/wk), moderate (>8.4-15.4 drinks/wk), heavy (>15.4-24.5 drinks/wk) and abusive (>24.5 drinks/wk) (eMethods 3 in the Supplement). We first assessed the prevalence and hazards of CVDs within each drinking group; the latter was estimated by Cox proportional hazards using abstainers as the reference groups. We then evaluated potential differences in smoking frequency, BMI, self-reported physical activity, cooked vegetable intake, red meat consumption, and self-reported health by drinking category to assess whether light to moderate alcohol consumption is associated with a healthier overall lifestyle. Adjusting for these 6 lifestyle factors, we reestimated hazards of CVD to assess for possible confounding (eMethods 7 in the Supplement).

We then conducted 2-sample MR, prioritizing inverse-variance weighted (IVW) meta-analyses of the association of each SNV with the outcome divided by the association of the same SNV with alcohol consumption; weighted median, MR-Egger, and MR–Pleiotropy Residual Sum and Outlier (MR-PRESSO) analyses were secondarily performed to address potential invalid instruments, outlying SNVs, and directional pleiotropy (eMethods 8 in the Supplement). In addition, we used allele score methods, which combine all externally weighted SNVs into a single instrument that is tested for association with each outcome (eMethods 9 in the Supplement). To additionally test for pleiotropy, analyses were repeated in lifelong abstainers, a population devoid of alcohol consumption, to assess any direct association between the genetic instrument and the outcome (eMethods 6 in the Supplement). For continuous traits, we considered significant any association surpassing a Bonferroni-corrected threshold of *P* < .005 [.05 / 10 traits] and, for cardiovascular diseases, a threshold of *P* < .008 [.05 / 6 diseases].

Although traditional (linear) MR estimates the change in odds of the outcome per change in the exposure, these analyses have limited ability to assess for nonuniform directionality of the exposure-outcome relationship or differential risks across levels of the exposure.^19^ To directly test for nonlinearity, the genetic association between exposure and outcome may be tested at various intervals of the exposure. This method allows for assessment of localized average causal effects in deciles of residual (IV-free) alcohol intake, which can be used to re-create the overall association using either fractional polynomial or piecewise linear methods (eMethods 10 in the Supplement). We applied NLMR methods as validated previously to test the shape of each potential association, prioritizing diseases and continuous traits with robust evidence from our traditional MR analyses.^18^ Sensitivity analyses included removal of abstainers and multivariable NLMR (eMethods 10 in the Supplement).^26^ All analyses were conducted using PLINK version 2.0 and R version 3.5 (R Project for Statistical Computing).

## Results

### Characteristics in the UK Biobank

Baseline characteristics of the 371 463 study participants from the UK Biobank are shown in Table 1. The mean (SD) age was 57.0 (7.9) years, 172 400 (46%) were men, and the mean (SD) alcohol consumption was 9.2 (10.6) standard drinks per week; 121 708 participants (33%) had hypertension, and 27 667 participants (7.5%) had CAD. Among light drinkers (mean [SD] consumption, 4.9 [2.7] drinks/week), alcohol intake comprised 38% beer, 29% red wine, 24% champagne or white wine, 6% spirits, 3% fortified wine, and 0.2% other alcoholic beverages; among heavy drinkers (mean [SD] consumption, 21 [3.8] drinks/week), alcohol intake comprised 38% beer, 24% red wine, 28% champagne or white wine, 7% spirits, 2% fortified wine, and 0.1% other alcoholic beverages (eTable 3 in the Supplement).

**Table 1.** Baseline Characteristics of Individuals in the UK Biobank

Characteristic	Participants, No. (%) (N = 371 463)
Age, mean (SD)	56.97 (7.93)
Men	172 400 (46.41)
Women	199 063 (53.49)
UK BiLEVE array	43 297 (11.66)
Weekly alcohol consumption, mean (SD)	9.16 (10.61)
BMI, mean (SD)	27.41 (4.75)
Blood pressure, mean (SD), mm Hg	
Systolic	140.23 (19.64)
Diastolic	82.26 (10.66)
Hypertension	121 708 (32.76)
Coronary artery disease	27 667 (7.45)
Myocardial infarction	14 503 (3.90)
Stroke	8710 (2.34)
Heart failure	5812 (1.56)
Atrial fibrillation	14 367 (3.87)

Abbreviation: BMI, body mass index (calculated as weight in kilograms divided by height in meters squared).

### Observational Associations With Cardiovascular Diseases and Lifestyle Factors

**Figure 1.**
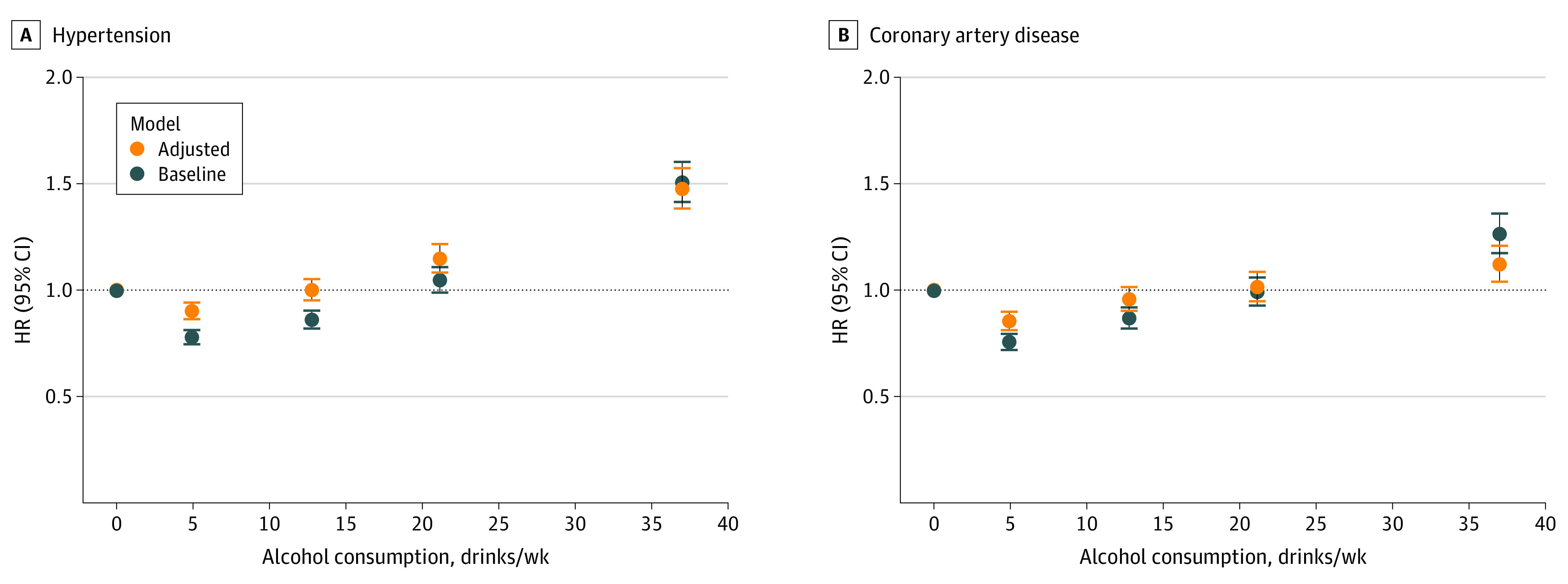
Epidemiological Associations Between Alcohol Consumption and Incident Cardiovascular Disease Baseline Cox proportional hazards models are shown in blue, and lifestyle-adjusted models are shown in orange. Lifestyle factors were smoking, body mass index, red meat intake, vegetable intake, physical activity, and self-reported health. Associations between subcategories of alcohol consumption and incident cardiovascular diseases are presented as hazard ratios for hypertension and coronary artery disease (CAD). Alcohol consumption is reported as US standard drinks per week, equivalent to 14 g of alcohol. Error bars represent the 95% CI.

Well-established J- or U-shaped curves were recapitulated for the association between alcohol consumption and both the prevalence and hazards of hypertension, CAD, MI, stroke, heart failure, and atrial fibrillation (Figure 1; eFigures 1 and 2 in the Supplement). However, individuals in the light and moderate consumption group had healthier lifestyle behaviors than abstainers, self-reporting better overall health and exhibiting lower rates of smoking, lower BMI, higher physical activity, and higher vegetable intake (eFigure 3 in the Supplement). Adjustment for the aforementioned lifestyle factors attenuated the cardioprotective associations with modest alcohol intake. For example, in baseline models, moderate intake was associated with significantly lower risk of hypertension and CAD, but adjustment for just 6 lifestyle factors rendered these results insignificant

### Associations Between Genetic Instruments and Alcohol Intake Phenotypes

The primary genetic instrument (AUD-R) strongly associated with alcohol intake in the UK Biobank (β = 7.0 standard drinks per week; *P* < .001), with a corresponding *F* statistic of 780. Genetic instruments were strongly associated with a range of alcohol phenotypes and did not appear to associate with confounders (eTables 4-6 in the Supplement).

### Traditional MR

Alcohol consumption due to the primary genetic instrument was associated with increased γ-glutamyl transferase level, a well-established marker of alcohol use,^29^ as well as increases in cardiovascular risk factors, such as SBP, DBP, and LDL cholesterol level. A lack of association in 34 423 lifelong abstainers and nonsignificant MR-Egger intercepts suggested no significant pleiotropy. However, a potential pleiotropic effect was detected in the association between alcohol and both HDL cholesterol and apolipoprotein A levels among lifelong abstainers. Associations of genetic instruments with 31 continuous phenotypes in current drinkers and lifelong abstainers, as well as in specific categories of alcohol consumption, are summarized in eTables 7 and 8 in the Supplement.

Genetic evidence supported strong associations between alcohol use and increased risk of hypertension and CAD (Table 2; eFigure 4 in the Supplement). In traditional 2-sample MR analyses, a 1-SD increase in genetically predicted alcohol consumption was associated with a higher risk of hypertension (IVW estimate: odds ratio [OR], 1.3; 95% CI, 1.2-1.4; *P* < .001) and CAD (IVW estimate: OR, 1.4; 95% CI, 1.1-1.8; *P* = .006). Secondary MR analyses (weighted median, MR-Egger, MR-PRESSO, and excluding abstainers) and MR analyses of other cardiovascular disease phenotypes supported the primary observations (Table 2; eFigure 4 and eTables 9 and 10 in the Supplement).

**Table 2.** Two-Sample Mendelian Randomization Estimates for Associations Between Alcohol Consumption and 6 Cardiovascular Phenotypes^a^

Cardiovascular phenotype	Odds ratio (95% CI)	*P* value
Hypertension	1.28 (1.18-1.39)	1.73 × 10^−9^
Coronary artery disease	1.38 (1.10-1.74)	6.00 × 10^−3^
Myocardial infarction	1.37 (1.05-1.78)	2.00 × 10^−2^
Stroke	1.26 (1.04-1.54)	2.10 × 10^−2^
Heart failure	1.39 (1.08-1.78)	9.00 × 10^−3^
Atrial fibrillation	1.24 (1.08-1.44)	3.00 × 10^−3^

^a^ Genetic instruments (the Alcohol Use Disorder–Restricted) were used to test for association with disease (in current drinkers) or for potential pleiotropy (in lifelong abstainers), as specified. Results are displayed for 2-sample mendelian randomization analyses using the inverse-variance weighted method, and odds ratios are reported per 1-SD increase in genetically predicted alcohol consumption. Bonferroni-corrected *P* < .008 was considered statistically significant.

To begin to assess the risk of cardiovascular disease associated with different levels of habitual alcohol consumption, we conducted allele score analyses stratified by amount of alcohol consumed (eFigure 5 in the Supplement). In both light and moderate drinkers, a 1–drink per day increase in the allele score was associated with at least nominally significantly increased odds of hypertension (light drinkers: OR, 1.3; 95% CI, 1.1-1.5; *P* = .003; moderate drinkers: OR, 1.7; 95% CI, 1.3-2.2; *P* < .001) and CAD (light drinkers: OR, 1.7; 95% CI, 1.2-2.4; *P* < .001; moderate drinkers: OR, 1.8; 95% CI, 1.1-2.8; *P* = .02). In abusive drinkers, a 1–drink per day increase in the allele score was associated with even greater risks of hypertension (OR, 2.6; 95% CI, 1.6-4.2; *P* < .001) and CAD (OR, 5.7; 95% CI, 2.4-13.5; *P* < .001). These patterns persisted after stratifying by sex (eTable 11 in the Supplement); for example, directionally consistent associations of genetically predicted alcohol intake with hypertension and CAD were observed in both male (eg, hypertension: OR, 1.4; 95% CI, 1.1-1.8; *P* = .01) and female (eg, CAD: OR, 1.7; 95% CI, 0.9-3.0; *P* < .09) light drinkers, although the results were not statistically significant for women.

### Evaluating Association Shapes

**Figure 2.**
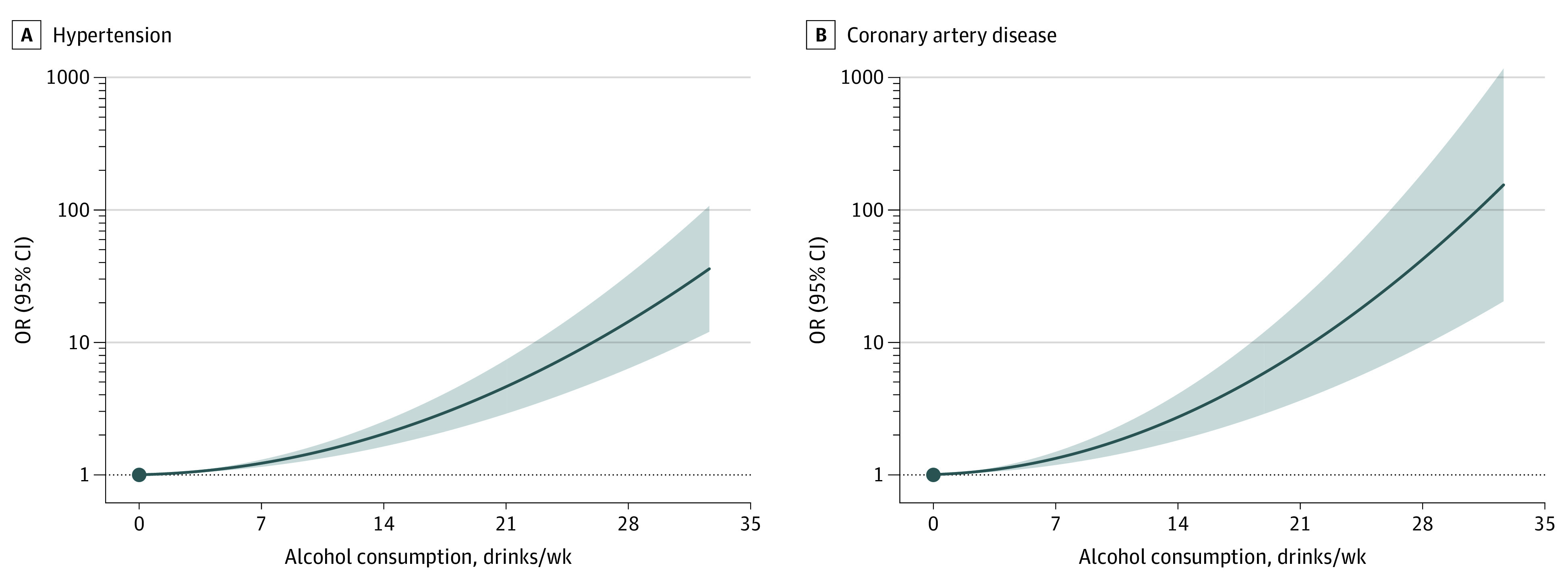
Genetic Associations of Alcohol Consumption With Cardiovascular Disease Phenotypes Using fractional polynomial nonlinear mendelian randomization analyses with Alcohol Use Disorder–Restricted instrument, localized average causal effects were metaregressed against mean consumption in strata of residual alcohol intake, and exposure-outcome associations were reconstructed as the derivative of the best fit model for hypertension and coronary artery disease. Alcohol consumption is reported as US standard drinks per week, with each standard drink equivalent to 14 g of alcohol. Solid lines refer to odds ratio (OR) estimates, and shaded areas denote 95% CIs for the model.

To better assess differential risk profiles across strata of alcohol consumption, we pursued NLMR analyses prioritizing outcomes with robust evidence from previous traditional MR analyses. Three separate statistical tests indicated that nonlinear models approximated the association between alcohol intake and both hypertension and CAD better than linear models (eTable 12A in the Supplement); specifically, quadratic models best fit these associations (both models, *P* < .001) (Figure 2). For each condition, all amounts of alcohol consumption were associated with an increased risk of disease. Furthermore, increased alcohol consumption was associated with increases in disease risk that were exponential and unequal in magnitude, even when comparing light and moderate levels of consumption (ie, between 1 and 2 drinks per day). Similar trends toward nonlinear and single-directional (ie, quadratic) associations were noted for other cardiovascular diseases and for all-cause mortality (eFigures 6 and 7 in the Supplement).

**Figure 3.**
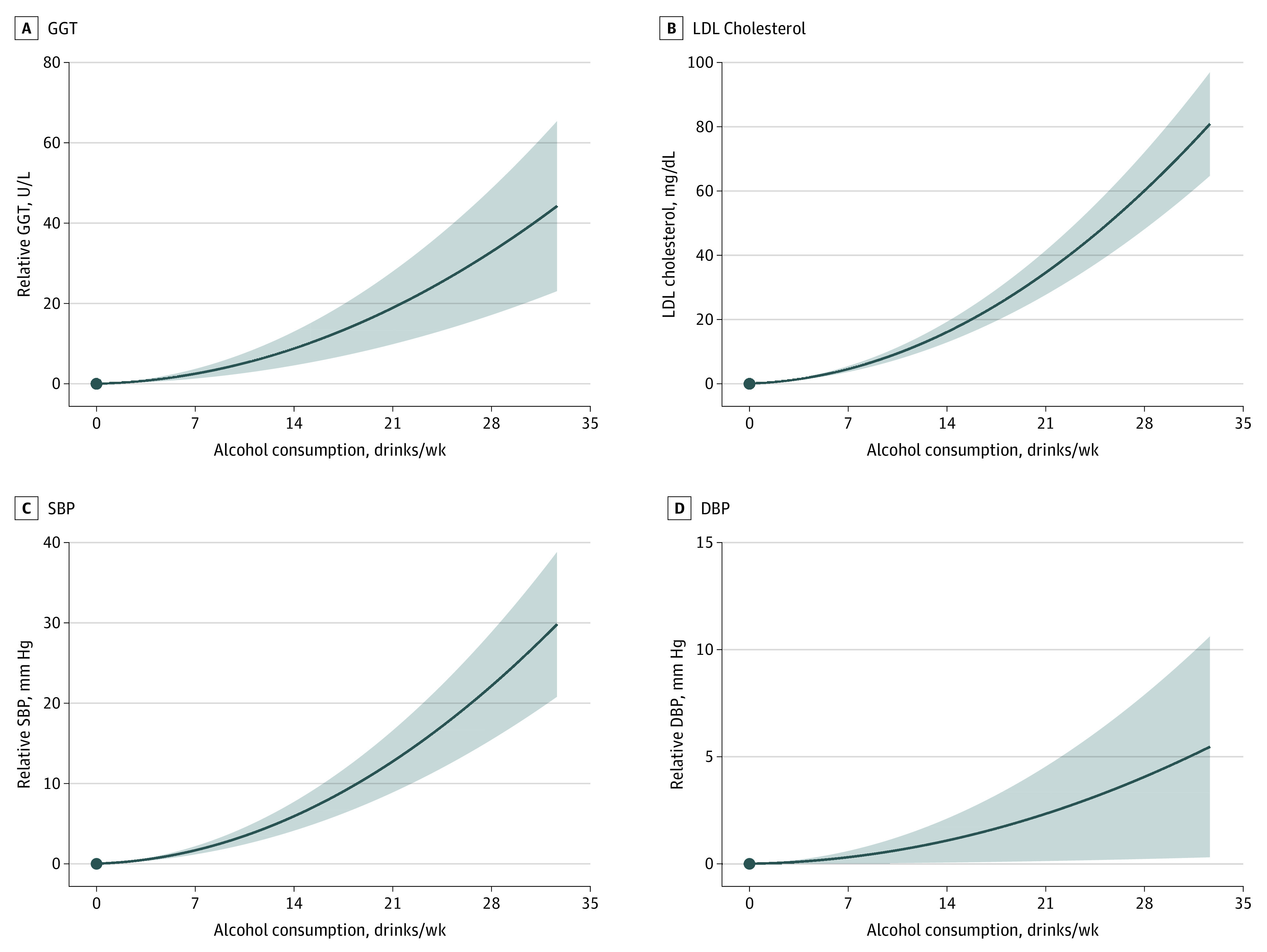
Genetic Associations of Alcohol Consumption With Continuous Traits and Cardiovascular Risk Factors Using fractional polynomial nonlinear mendelian randomization analyses with Alcohol Use Disorder–Restricted instruments, localized average causal effects were metaregressed against mean consumption in each strata of alcohol, and these plots were reconstructed as the derivative of the best fit model for γ-glutamyl transferase (GGT), low-density lipoprotein (LDL) cholesterol, systolic blood pressure (SBP), and diastolic blood pressure (DBP). Alcohol consumption is reported as US standard drinks per week, with each standard drink equivalent to 14 g of alcohol. Solid lines refer to odds ratio (OR) estimates, and shaded areas denote 95% CIs for the model.

NLMR also suggested nonlinear associations between alcohol intake and continuous risk factors, such as SBP and LDL cholesterol levels (eTable 12B in the Supplement), with consistently positive and quadratic associations observed between alcohol consumption and SBP (model, *P* < .001), DBP (model, *P* = .001), and LDL cholesterol level (model, *P* < .001) (Figure 3).

Sensitivity analyses using different genetic instruments (AUDIT-C-R score and also a single SNV at the biologically relevant *ADH1B* gene) and excluding abstainers largely supported the primary observations, as did secondary sex-stratified analyses and use of medication-adjusted values of SBP and DBP (eFigures 8-13 and eTable 12C and D in the Supplement). Tests of nonlinearity using the AUDIT-C-R instrument further supported nonlinear associations between alcohol intake and all primary outcomes, although, notably, piecewise linear analyses suggested a threshold effect, wherein alcohol intake was not associated with increased cardiovascular risk until roughly 7 to 14 drinks per week. Slight decreases in cardiovascular risk were observed with modest alcohol intake when associating the secondary AUDIT-C-R score with hypertension and in select secondary analyses conducted in women (eFigures 8 and 12 in the Supplement). However, similar patterns were not observed with the single SNV instrument at *ADH1B*; for analyses of LDL cholesterol level, SBP, and DBP in women; when removing abstainers; or when conducting linear MR in female light drinkers, thereby suggesting that these findings were likely attributable to residual pleiotropy in the AUDIT-C-R instrument and reduced power of sex-stratified NLMR analyses, respectively (eFigures 5, 8, and 10-12 and eTable 11 in the Supplement). Multivariable NLMR adjusting for smoking, BMI, and depression supported the primary observations (eFigure 14 in the Supplement).

### Nonlinear Genetic Associations in the Mass General Brigham Biobank

We assessed for similar nonlinear associations in the Mass General Brigham Biobank (30 716 participants) (eTable 13 in the Supplement). The primary genetic instrument was strongly associated with habitual alcohol intake in the Mass General Brigham Biobank and showed directionally consistent associations with DBP and directionally consistent results with SBP (eTable 14A in the Supplement). NLMR again yielded quadratic models as those best capturing the associations between alcohol consumption and DBP (model, *P* < .001), suggesting exponential increases in DBP with progressively greater alcohol intake (eTable 14B and eFigure 15 in the Supplement). The results for SBP were not statistically significant.

## Discussion

In this study, we assessed the association of habitual alcohol consumption with cardiovascular disease risk. Epidemiological analyses identified that coincident lifestyle factors may confound established observational trends. Human genetic data suggested causal associations between alcohol intake and risk of hypertension and CAD that increase with even modest alcohol consumption and are exponential in magnitude.

These results permit several conclusions. First, the reported cardioprotective effects of light to moderate alcohol consumption may be the product of confounding lifestyle factors. Consistent with prior studies, we found J- and U-shaped epidemiologic curves for the association of alcohol intake with cardiovascular disease, but we also found that light to moderate alcohol consumers exhibited healthier lifestyles than abstainers.^10,11^ Adjusting for only a few lifestyle factors ascertained by the UK Biobank, we observed attenuation in the apparent protective associations between modest alcohol intake and cardiovascular risk, suggesting that adjustments for yet unmeasured or unknown factors may further attenuate—if not, eliminate—the residual, cardioprotective associations observed among light drinkers.

Second, human genetic evidence is suggestive of a causal relationship between alcohol consumption and cardiovascular disease that is consistently risk increasing, with the magnitude of risk rising exponentially at higher levels of intake. Here, using linear MR, we added to the evidence base that alcohol intake may be associated with a range of cardiovascular diseases and risk factors.^14,15,16,17^ However, applying NLMR and formal tests for nonlinearity, we found that light alcohol consumption was associated with minimal cardiovascular risk, but similar to recent epidemiological findings, risk of cardiovascular disease increased exponentially at higher levels of intake.^8^ These results carry at least 2 important clinical implications: (1) they substantiate prior claims that no amount of alcohol is protective against cardiovascular disease, and (2) they newly demonstrate that the adverse effects of alcohol unduly affect those who consume heavily, implying that for an equivalent reduction in alcohol intake, the improvements to cardiovascular health may be significant for heavier drinkers but only slight for those who consume modestly.

Third, the substantial differences in cardiovascular risk across the spectrum of alcohol consumption may have important implications for clinical and public health recommendations around habitual alcohol use. Specifically, our results suggest that consuming as many as 7 drinks per week is associated with relatively modest increases in cardiovascular risk. However, nonlinear modeling uncovered unequal increases in cardiovascular risk when progressing from 0 to 7 vs 7 to 14 drinks per week in both men and women. Although risk thresholds are inherently somewhat subjective, these findings again bring into question whether an average consumption of 2 drinks per day (14 drinks per week) should be designated a low-risk behavior.^8,20,21,22^ Furthermore, as several-fold increases in risk were observed for those consuming 21 or more drinks per week, our results emphasize the importance of aggressive efforts to reduce alcohol intake among heavy drinkers.

### Limitations

The present study has several limitations. First, despite efforts to minimize the effects of pleiotropy, it remains possible that the associations between alcohol intake and cardiovascular disease represent a shared genetic basis, rather than a direct causal relationship. Second, our primary genetic instrument comprised SNVs associated with a diagnosis of AUD—an indirect measure of alcohol use—rather than SNVs directly associated with a continuous measure of alcohol consumption; notably, there may be some differences in the genetic architecture of habitual alcohol consumption and AUD. However, we empirically evaluated our instruments in the UK Biobank and found that the AUD genetic instrument demonstrated strong associations with all tested alcohol phenotypes, including weekly intake and drinking category, indicating that the instrument did not simply reflect a susceptibility to heavy alcohol consumption. Similarly, the AUDIT-C questionnaire is also designed to screen for heavy alcohol consumption rather than habitual alcohol consumption.^30^ Nevertheless, future assessments testing our genetic instruments—as well as others for continuous alcohol consumption—in additional, large genetic data sets will be of importance.

## Conclusions

The findings of this study suggest that the observed cardioprotective effects of light to moderate alcohol intake may be largely mediated by confounding lifestyle factors. Genetic analyses suggest causal associations between alcohol intake and cardiovascular disease but with unequal and exponential increases in risk at greater levels of intake, which should be accounted for in health recommendations around the habitual consumption of alcohol.

## Notes

Supplement. **eMethods 1.** UK Biobank Data Collection **eMethods 2.** Exclusions **eMethods 3.** Alcohol Variables **eMethods 4.** Continuous Variables **eMethods 5.** Mass General Brigham Biobank **eMethods 6.** Genetic Instruments **eMethods 7.** Observational Analysis **eMethods 8.** Two-Sample MR Analyses **eMethods 9.** Allele Score Analyses **eMethods 10.** Nonlinear MR **eTable 1.** Single-Nucleotide Variants Included in Genetic Instruments **eTable 2**. Definitions of Cardiovascular Disease Phenotypes in the UK Biobank **eTable 3. **Mean Weekly Standard Measures (1 measure, 14 g) in Drinking Categories and Beverage Composition of Alcohol Consumption **eTable 4.** Assessments for Pleiotropy in Single-Nucleotide Variants in Lifelong Abstainers **eTable 5. **Assessments of MR Assumptions in Primary Genetic Instrument **eTable 6. **Assessments of MR Assumptions in Secondary Genetic Instruments **eTable 7. **Primary Genetic Associations Between Alcohol and Continuous Traits **eTable 8.** Secondary Genetic Associations Between Alcohol and Continuous Traits **eTable 9.** Secondary Genetic Associations Between Alcohol and Cardiovascular Disease Phenotypes **eTable 10.** MR-PRESSO Sensitivity Analyses for 2SMR Analyses **eTable 11.** Sex-Stratified Allele Score Associations Between Alcohol and Primary Cardiovascular Disease Outcomes **eTable 12. **Nonlinear MR Tests **eTable 13.** Study Characteristics in Mass General Brigham Biobank **eTable 14.** Associations of AUD-R Allele Score With Alcohol Consumption and Blood Pressure Measurements in Mass General Brigham Biobank **eFigure 1.** Alcohol Consumption and Prevalence of Cardiovascular Diseases **eFigure 2. **Secondary Analyses for Confounding in Epidemiological Associations Between Alcohol Consumption and Cardiovascular Disease **eFigure 3.** Mean Values of 6 Different Lifestyle Factors Within Alcohol Consumption Subcategories **eFigure 4. **Genetic Associations of Alcohol With CVD Phenotypes Using Allele Scores **eFigure 5. **Genetic Allele Score Associations of Alcohol With CVD Phenotypes Stratified by Category of Alcohol Consumption **eFigure 6.** Fractional Polynomial Nonlinear MR Analyses, Using AUD-R Genetic Instruments, of Alcohol and Secondary Cardiovascular Disease Phenotypes **eFigure 7. **Nonlinear MR Analyses of Alcohol and Total Mortality **eFigure 8.** Fractional Polynomial Nonlinear MR Analyses, Using Secondary Genetic Instruments, of Alcohol and 6 Cardiovascular Disease Phenotypes **eFigure 9. **Piecewise Nonlinear MR Analyses of Alcohol and 6 Cardiovascular Disease Phenotypes **eFigure 10. **Fractional Polynomial Nonlinear MR Analyses of Alcohol and Continuous Traits **eFigure 11. **Piecewise Nonlinear MR Analyses of Alcohol and Continuous Traits **eFigure 12. **Sex-Stratified Nonlinear MR Analyses for Primary Outcomes **eFigure 13.** Nonlinear MR Analyses of Alcohol and Medication-Corrected Blood Pressure **eFigure 14. **Multivariable Fractional Polynomial Nonlinear MR Analyses, Adjusting for Smoking, BMI, and Depression **eFigure 15.** Fractional Polynomial Nonlinear MR Analyses of Alcohol and Blood Pressure in Mass General Brigham Biobank
eReferences.
