# Error in Abstract

**DOI:** 10.1001/jamanetworkopen.2022.12024

**Published:** 2022-04-20

In the Original Investigation titled “Association of Habitual Alcohol Intake With Risk of Cardiovascular Disease,”^1^ published March 25, 2022, there was an error in the Abstract. The first sentence of the Conclusions and Relevance section should have read “In this cohort study, adjustment for coincident, favorable lifestyle factors attenuated the observational benefits of modest alcohol intake.” This article has been corrected.^1^
