# Supplementary material for: Association of Habitual Alcohol Intake With Risk of Cardiovascular Disease
Source: JAMA Netw Open. 2022 Mar 25;5(3):e223849. doi: 10.1001/jamanetworkopen.2022.3849 (PMC8956974; doi:10.1001/jamanetworkopen.2022.3849)
Supplement: Supplement. — eMethods 1. UK Biobank Data Collection eMethods 2. Exclusions eMethods 3. Alcohol Variables eMethods 4. Continuous Variables eMethods 5. Mass General Brigham Biobank eMethods 6. Genetic Instruments eMethods 7. Observational Analysis eMethods 8. Two-Sample MR Analyses eMethods 9. Allele Score Analyses eMethods 10. Nonlinear MR eTable 1. Single-Nucleotide Variants Included in Genetic Instruments eTable 2. Definitions of Cardiovascular Disease Phenotypes in the UK Biobank eTable 3. Mean Weekly Standard Measures (1 measure, 14 g) in Drinking Categories and Beverage Composition of Alcohol Consumption eTable 4. Assessments for Pleiotropy in Single-Nucleotide Variants in Lifelong Abstainers eTable 5. Assessments of MR Assumptions in Primary Genetic Instrument eTable 6. Assessments of MR Assumptions in Secondary Genetic Instruments eTable 7. Primary Genetic Associations Between Alcohol and Continuous Traits eTable 8. Secondary Genetic Associations Between Alcohol and Continuous Traits eTable 9. Secondary Genetic Associations Between Alcohol and Cardiovascular Disease Phenotypes eTable 10. MR-PRESSO Sensitivity Analyses for 2SMR Analyses eTable 11. Sex-Stratified Allele Score Associations Between Alcohol and Primary Cardiovascular Disease Outcomes eTable 12. Nonlinear MR Tests eTable 13. Study Characteristics in Mass General Brigham Biobank eTable 14. Associations of AUD-R Allele Score With Alcohol Consumption and Blood Pressure Measurements in Mass General Brigham Biobank eFigure 1. Alcohol Consumption and Prevalence of Cardiovascular Diseases eFigure 2. Secondary Analyses for Confounding in Epidemiological Associations Between Alcohol Consumption and Cardiovascular Disease eFigure 3. Mean Values of 6 Different Lifestyle Factors Within Alcohol Consumption Subcategories eFigure 4. Genetic Associations of Alcohol With CVD Phenotypes Using Allele Scores eFigure 5. Genetic Allele Score Associations of Alcohol With CVD Phenotypes Stratified by Category of Alcohol Consumption eFi [file jamanetwopen-e223849-s001.pdf]

Supplemental Online Content

Biddinger KJ, Emdin CA, Haas ME, et al. Association of Habitual Alcohol Intake With Risk of Cardiovascular Disease. *JAMA Netw Open.* 2022;5(3):e223849. doi:10.1001/jamanetworkopen.2022.3849

eMethods 1. UK Biobank Data Collection

eMethods 2. Exclusions

eMethods 3. Alcohol Variables

eMethods 4. Continuous Variables

eMethods 5. Mass General Brigham Biobank

eMethods 6. Genetic Instruments

eMethods 7. Observational Analysis

eMethods 8. Two-Sample MR Analyses

eMethods 9. Allele Score Analyses

eMethods 10. Nonlinear MR

eTable 1. Single-Nucleotide Variants Included in Genetic Instruments

eTable 2. Definitions of Cardiovascular Disease Phenotypes in the UK Biobank

eTable 3. Mean Weekly Standard Measures (1 measure, 14 g) in Drinking Categories and Beverage Composition of Alcohol Consumption

eTable 4. Assessments for Pleiotropy in Single-Nucleotide Variants in Lifelong Abstainers

eTable 5. Assessments of MR Assumptions in Primary Genetic Instrument

eTable 6. Assessments of MR Assumptions in Secondary Genetic Instruments

eTable 7. Primary Genetic Associations Between Alcohol and Continuous Traits

eTable 8. Secondary Genetic Associations Between Alcohol and Continuous Traits

eTable 9. Secondary Genetic Associations Between Alcohol and Cardiovascular Disease Phenotypes

eTable 10. MR-PRESSO Sensitivity Analyses for 2SMR Analyses

eTable 11. Sex-Stratified Allele Score Associations Between Alcohol and Primary Cardiovascular Disease Outcomes

eTable 12. Nonlinear MR Tests

eTable 13. Study Characteristics in Mass General Brigham Biobank

eTable 14. Associations of AUD-R Allele Score With Alcohol Consumption and Blood Pressure Measurements in Mass General Brigham Biobank

eFigure 1. Alcohol Consumption and Prevalence of Cardiovascular Diseases

© 2022 Biddinger KJ et al. *JAMA Network Open.*

eFigure 2. Secondary Analyses for Confounding in Epidemiological Associations Between Alcohol Consumption and Cardiovascular Disease

eFigure 3. Mean Values of 6 Different Lifestyle Factors Within Alcohol Consumption Subcategories

eFigure 4. Genetic Associations of Alcohol With CVD Phenotypes Using Allele Scores

eFigure 5. Genetic Allele Score Associations of Alcohol With CVD Phenotypes Stratified by Category of Alcohol Consumption

eFigure 6. Fractional Polynomial Nonlinear MR Analyses, Using AUD-R Genetic Instruments, of Alcohol and Secondary Cardiovascular Disease Phenotypes

eFigure 7. Nonlinear MR Analyses of Alcohol and Total Mortality

eFigure 8. Fractional Polynomial Nonlinear MR Analyses, Using Secondary Genetic Instruments, of Alcohol and 6 Cardiovascular Disease Phenotypes

eFigure 9. Piecewise Nonlinear MR Analyses of Alcohol and 6 Cardiovascular Disease Phenotypes

eFigure 10. Fractional Polynomial Nonlinear MR Analyses of Alcohol and Continuous Traits

eFigure 11. Piecewise Nonlinear MR Analyses of Alcohol and Continuous Traits

eFigure 12. Sex-Stratified Nonlinear MR Analyses for Primary Outcomes

eFigure 13. Nonlinear MR Analyses of Alcohol and Medication-Corrected Blood Pressure

eFigure 14. Multivariable Fractional Polynomial Nonlinear MR Analyses, Adjusting for Smoking, BMI, and Depression

eFigure 15. Fractional Polynomial Nonlinear MR Analyses of Alcohol and Blood Pressure in Mass General Brigham Biobank

eReferences.

This supplemental material has been provided by the authors to give readers additional information about their work.

© 2022 Biddinger KJ et al. *JAMA Network Open.*

eMethods 1. UK Biobank Data Collection

Analyses were conducted in the UK Biobank, a prospective cohort from the United Kingdom. Nine million individuals were invited to participate, of which 502 629 subjects were enrolled and surveyed at assessment centers across the UK from 2006-2010.[1] Participants ranged in age from 40 to 69 years. Data collection included verbal interviews and touchscreen questionnaires to gather a multitude of lifestyle information, including alcohol consumption, physical activity, and vegetable intake. All subjects were genotyped using either the Affymetrix UK BiLEVE Axiom array (first 50 000 subjects) or the Affymetrix UK Biobank Axiom® array (remaining 450 000 participants). Subjects also underwent blood, urine, and saliva samples for analysis. Blood biochemistry analyses were used to determine biomarker levels. Prevalent cardiovascular diseases were recorded at study entry through self-report confirmed in a verbal interview with a trained nurse, or via linked electronic health record data from the National Health Service (NHS). Incident diseases were defined, among those not meeting criteria at baseline, through the application of phenotype definitions to linked, in-patient hospital and death registry data. Participants were censored at whichever came first between disease diagnosis, date of death, or date of last follow-up. The date of last follow-up was February 9, 2016 for participants enrolled in Wales, February 16, 2016 for participants enrolled in England, and October 31, 2015 for participants enrolled in Scotland. Region-specific censor dates are available at https://biobank.ctsu.ox.ac.uk/crystal/exinfo.cgi?src=Data_providers_and_dates.

© 2022 Biddinger KJ et al. *JAMA Network Open.*

eMethods 2. Exclusions

We excluded participants with unreliable data, which was defined – per centralized sample quality control performed by UK Biobank – as inferred sex unequal to reported sex, kinship not inferred, putative sex chromosome aneuploidy, withdrawn consent, or excessive heterozygosity or missingness in genetic data.[2] For subjects of 2nd degree or closer relatedness, one subject was randomly removed to prevent individuals with similar lifestyle factors and genetics from skewing the data. We further limited analyses to participants of European genetic ancestry to minimize potential confounding of Mendelian randomization analyses by genetic ancestry. The remaining subjects (n=376 424) were subset to those without missingness in alcohol reporting data (n=371 463). All of these individuals had genetic data, alcohol data, data for the primary covariates (age, sex, genotyping array, and PCs), and data for the 6 disease outcomes (except for 150 individuals with missing heart failure values, who were excluded from analyses of heart failure). Of the primary continuous outcomes, 18 025 individuals had missing LDL-C values, 24 532 had missing SBP values, 24 524 had missing DBP values, and 17 550 had missing GGT values; individuals with missing outcome data were excluded from those analyses.

© 2022 Biddinger KJ et al. *JAMA Network Open.*

eMethods 3. Alcohol Variables

Participants self-reported their alcohol intake upon assessment by typing in answers on a touchscreen. Subjects were first asked roughly how frequently they consumed alcohol (e.g. daily/almost daily, 3-4 times a week, once or twice a week, one to three times a month, special occasions only, or never), and, based on whether they drank more than one to three times a month, were then asked to report their consumption for each drink (above) either per week (more than 1-3 times/month) or per month (1-3 times/month or less). We aggregated this data to construct the following variables:

Weekly alcohol: A standardized variable of American measures of alcohol consumption per week, with 1 standard drink equivalent to 14 g of alcohol. The variable was created by calculating total alcohol content for each reported drink using British measurements (1 pint of beer or cider=16g, 1 standard glass of white/red wine=16.8g, 1 glass of fortified wine=14.08g, 1 shot of spirits or liquor=8g, 1 glass of other alcohol drinks (i.e. alcopops)=12g).[3 4] Then, grams of alcohol were converted to American standard measures, with 14g equal to 1 measure (1 pint of beer or cider=1.14 measures, 1 standard glass of white/red wine=1.2 measures, 1 glass of fortified wine=1.01 measures, 1 shot of spirits or liquor=0.57 measures, 1 glass of other alcohol drinks (i.e. alcopops)=0.86 measures).[5]

Ln(weekly alcohol): Created by taking the natural log of weekly standard drinks added to one to prevent removal of nondrinker (0) values. This log transformation gave the data a more normalized distribution for statistical testing.

Drinking group: Created as a categorical variable to batch subjects into five distinct groups using definitions derived from physicians' categorization of alcohol use.[6] By multiplying these daily limits by 7, groups were defined as: abstainers (0 drinks/week), light (0-8.4 drinks/week), moderate (8.4-15.4 drinks/week), heavy (15.4-24.5 drinks/week) and abusive (>24.5 drinks/week).

Current drinker status: Created as a binomial variable defined as 1 if the subject reported currently consuming alcohol (weekly alcohol > 0) or 0 if the subject did not (weekly alcohol = 0).

Over weekly limits: Created as a binomial variable defined as 1 if the subject consumed alcohol over weekly limits, stratified by sex, or 0 if the subject did not. Weekly limits were calculated from the "Dietary Guidelines for Americans 2015-2020" by the U.S. Department of Health and Human Services and U.S. Department of Agriculture recommendations for low-risk drinking (<=14 standard drinks/week for men and <=7 drinks/week for women).[5]

© 2022 Biddinger KJ et al. *JAMA Network Open.*

eMethods 4. Continuous Variables

For each trait, means and statistical differences were calculated after removing unreported values for the individual category. The variables were collected as follows. BMI was calculated from measured height and weight at assessment. Smoking was a self-reported categorical variable, defined as never smoked (0), previously smoked (1), or current smoker (2). Physical activity was a self-reported variable representing the average number of days per week during which the participant spent more than 10 minutes doing moderate physical activity. Vegetable intake was a self-reported variable representing the average number of heaped tablespoons of cooked vegetables that a subject would eat per day. Red meat was another self-reported variable calculated as the sum of the participant's frequency of eating beef, lamb, or pork. Eating frequency was labeled as (0) never, (1) less than once a week, (2) once a week, (3) 2-4 times per week, (4) 5-6 times per week, or (5) once or more per day. Overall health was a self-reported categorical variable of the participant's rating of their own overall health defined as (1) excellent, (2) good, (3) fair, or (4) poor. BMI, biomarkers, and blood pressure were normalized to follow a normal distribution in traditional genetic analyses (analyses in which the focus was testing for significant association), but not for non-linear genetic analyses (in which the focus was on clinically interpretable findings).

© 2022 Biddinger KJ et al. *JAMA Network Open.*

eMethods 5. Mass General Brigham Biobank

The Mass General Brigham Biobank – a patient-based cohort based in the United States – was used as a secondary cohort to replicate select genetic analyses primarily conducted in the UK Biobank.[7] The biobank consisted of 30,716 individuals with genetic data, 14,412 of whom had self-reported alcohol consumption information and 28,179 of whom had blood pressure measurements. If a participant had more than one blood pressure measurement, the maximum value was used in analyses. Participants were asked to group their alcohol consumption patterns into categorizations ranging from "None, or less than 1 drink per month" to "More than 6 drinks per day". In order to maximize power for analyses conducted in Mass General Brigham, only the primary genetic score (AUD-R) was used as the genetic instrument, and only continuous blood pressure measurements were used as the outcome.

© 2022 Biddinger KJ et al. *JAMA Network Open.*

eMethods 6. Genetic Instruments

Genetic instruments were constructed using externally-derived summary statistics from a genome-wide association study (GWAS) of alcohol consumption in 274 424 subjects from the Million Veteran Program, focusing on analyses conducted in Eastern Europeans to reflect the UK Biobank study population.[8] This study assessed genetic associations with Alcohol Use Disorder (AUD) and Alcohol Use Disorder Identification Test-Consumption (AUDIT-C), each used as a proxy for alcohol use. AUD is a medical diagnosis of severe overdrinking. AUDIT-C is a screening questionnaire meant to identify hazardous alcohol use; it is a three-question exam scored from 0-12 that asks (a) “how often do you have a drink containing alcohol?”, (b) “how many standard drinks do you have on a typical day”, and (c) “how often do you have six or more drinks on one occasion?”.[9] From all significant loci for AUD (n=13), only independent SNPs after conditional analyses were chosen, with insertion/deletion polymorphisms removed (n=9 total SNPs remaining). From all significant loci in the AUDIT-C GWAS (n=19), only independent SNPs after conditional analyses were chosen; no insertion/deletion polymorphisms were among the lead associations (n=13 total SNPs remaining).

Mendelian randomization assumes that a genetic instrument influences an exposure, and that the corresponding change in the exposure is the only way by which the instrument affects the outcome. Therefore, refined SNP lists were created by removing SNPs that had any significant associations with tested lifestyle or risk factors in lifelong abstainers. In lifelong abstainers – a population in which no one had ever consumed alcohol, and therefore associations could not be mediated by differential alcohol intake – any associations would therefore be due to pleiotropy in the instrument. Accordingly, insignificant associations with lifestyle/risk factors in lifelong abstainers was taken to demonstrate a lack of pleiotropy. The tested potential confounders were smoking, BMI, physical activity, vegetable intake, red meat intake, overall health rating, C-Reactive Protein, and total cholesterol. For the AUD SNP list (n=9), the Bonferroni p-value was Bonferroni p = 0.05/(9 SNPs * 8 confounders)=6.94E-04. For the AUDIT-C SNP list (n=13), the Bonferroni p-value was Bonferroni p = 0.05/(13 SNPs * 8 confounders)=4.801E-04. Four SNPs from the AUD and three SNPs from the AUDIT-C instruments were significantly associated with confounders and consequently removed for the refined SNP lists. From the AUD instrument, rs1260326 (GCKR), rs570436 (SIX3), rs13107325 (SLC39A8), and rs11075992 (FTO) were removed to create the AUD-R instrument. From the AUDIT-C instrument, rs1260326 (GCKR), rs13107325 (SLC39A8), and rs9937709 (FTO) were removed to create the AUDIT-C-R instrument. The remaining AUD-R SNP list contained 5 SNPs, and the remaining AUDIT-C-R SNP list contained 10 SNPs.

Aggregated allele scores were constructed using PLINK software v2.0. Allele scores were normalized and then standardized to a 1-drink/day (7 standard drinks/week) increase using R software. Associations with allele scores and confounders, calculated using linear regression models in lifelong abstainers, were used to assess for pleiotropic effect in associations, and results are available in eTables 5-6. Genetic risk scores were validated for association with the exposure by regressing several alcohol phenotypes--both log-transformed and untransformed weekly alcohol intake, drinking group, current drinking status, and over limits drinking status--onto the score. Individual drinks, such as beer intake or wine intake, were also regressed onto the score for validation. For all continuous variables, linear regression was used. For all dichotomous variables, logistic regression was used. In this study, all regression models for the gene scores were adjusted for age at assessment, sex, genotyping array, and principal components 1-10. Genotyping array represented a dummy variable to account for the specific genotyping platform used for each subject (Affymetrix UK BiLEVE Axiom array or the Affymetrix UK Biobank Axiom® array). Adjusting for principal components accounts for population stratification according to ancestral background.[10]

While both AUD-R and AUDIT-C-R scores were not associated with confounders in lifelong abstainers, 2SMR analyses between the AUDIT-C-R score and CVD phenotypes revealed significant MR-Egger intercepts for multiple associations (eTable S9), whereas these intercepts were insignificant when the AUD-R score was used (Table 2); MR-PRESSO tests for horizontal pleiotropy were also inflated for the AUDIT-C-R, but not AUD-R, instrument

© 2022 Biddinger KJ et al. *JAMA Network Open.*

(eTable S10). As noted below (Two-Sample MR Analyses section), MR-Egger intercepts are used to assess for pleiotropic effects in two sample MR analyses. Two sample MR analyses determine the association between exposure and outcome by assessing for a linear relationship between, for each SNP, association dosage with exposure and association dosage with the outcome. A significant y-intercept – assessed via MR-Egger analyses – implies that there is an association with the outcome that is independent of the exposure; in other words, some of the association is not mediated by alcohol intake. Nominally significant MR-Egger intercepts using AUDIT-C-R suggested a potential bias in the instrument.[11] One source for potential residual pleiotropy in the AUDIT-C-R instrument could have been the SNPs rs4794018 and rs35572189, which both reached nominal, but not Bonferroni-level, significance for associations with BMI in lifelong abstainers (both p = 0.002). Given these potential biases that were present in the AUDIT-C-R instrument but not in the AUD-R instrument, the AUD-R instrument was selected to be the primary instrument for this study, with the AUDIT-C-R used for secondary analyses.

Using the AUD-R primary instrument, a test for trend indicated that the genetic association with alcohol was not significantly different in categories of alcohol consumption (p=0.311), and genetically predicted alcohol values ranged from 1.65 to 14.08 drinks/week.

© 2022 Biddinger KJ et al. *JAMA Network Open.*

eMethods 7. Observational Analysis

We focused on six cardiovascular disease phenotypes: hypertension, coronary artery disease, myocardial infarction, stroke, heart failure, and atrial fibrillation. We assessed the prevalence and hazards of cardiovascular diseases within each drinking group, the latter estimated by cox proportional hazards using abstainers as reference and incident disease as the outcome. Analyses were conducted using the cox proportional hazards function in R from the 'Survival' package. Abstainers who reported formerly drinking (n=12,977) were removed from analyses as they may exhibit the residual health effects of alcohol. We then evaluated other behavioral and lifestyle factors by drinking category to assess whether light to moderate alcohol consumption correlates with a healthier overall lifestyle. Six specific lifestyle measures were assessed--smoking frequency, normalized BMI, self-reported physical activity, cooked vegetable intake, red meat consumption, and self-reported health. Though BMI can be influenced by alcohol, this study followed the precedent set by others that have labeled BMI as a lifestyle factor rather than an outcome.[12] [13] Adjusting for these six lifestyle factors, we re-estimated hazards of CVD by drinking group to assess for a change in the shape of observational associations due to possible confounding. In secondary analyses, we re-ran adjusted models without self-reported health, and also trialed sex-stratified models. Secondary analyses were performed for hypertension only, the most prevalent of all cardiovascular phenotypes assessed.

© 2022 Biddinger KJ et al. *JAMA Network Open.*

eMethods 8. Two-Sample MR Analyses

Two sample Mendelian randomization was conducted using the R packages 'Mendelian Randomization' and 'TwoSampleMR'. Summary statistics from an alcohol consumption GWAS in MVP (Kranzler et. al) provided beta coefficients and standard errors between each SNP and the exposure.[8] Regression models of each SNP in the gene score with the outcome of interest were used to calculate the beta coefficient and standard error between each SNP and the exposure. These datasets were combined and used for the two sample Mendelian randomization.

Inverse variance weighted (IVW) meta-analyses of the SNP-specific associations with the exposure and outcome provide a weighted average of the slope estimates from the origin to each point. For each SNP, the association with the exposure (alcohol) and the association with the outcome (CVD) are compared. If the exposure is causally associated with a greater risk of the outcome, SNPs associated with a greater change in exposure should be associated with a greater risk in the outcome (proportionality/linearity of exposure-associations and outcome-associations for each SNP). IVW tests the significance of the relationship between the two associations and provides an estimate of the effect of the exposure on the outcome. Weighted median analyses, which use similar calculations with only the median SNPs, provide an estimate that is less likely to be biased by outlier SNPs. MR-Egger analyses, which are similar to previous analyses but allow for a non-zero intercept in analysis, were used to check for pleiotropy. IVW and weighted median methods rely on the assumption that there is no y-intercept; in other words, that the relationship with the outcome of each SNP in the dataset is directly proportional to its association with the exposure, and that there is no inflation due to associations with confounders or the outcome itself. MR Egger models allow for a non-zero intercept in order to check if the pleiotropy condition is being violated. MR-PRESSO analyses, conducted using the MRPRESSO package in R, were employed to assess and correct for horizontal pleiotropy by removing outliers. Associations using these methods were primarily tested using outcome statistics in current drinkers and checked for pleiotropy by calculating outcome statistics in nondrinkers.

Using traditional two-sample MR methods to assess for potential causal associations, we investigated genetic associations of alcohol consumption with the six aforementioned cardiovascular diseases, and also with ten continuous traits: systolic and diastolic blood pressure, LDL cholesterol, HDL cholesterol, total cholesterol, triglycerides, apolipoproteins A and B, gamma-glutamyl transferase, and C reactive protein. For each instrument-outcome association, we pursued an IVW random-effects meta-analysis of the effect of each SNP on the outcomes divided by the effect of the same SNP on alcohol consumption. We also pursued weighted median and MR-Egger regression analyses to address potential invalid instruments and directional pleiotropy. For continuous traits, we considered significant any association surpassing a Bonferroni-corrected threshold of $p < 0.005$ [0.05/10 traits], and for cardiovascular diseases, a threshold of $p < 0.008$ [0.05/6 diseases].

© 2022 Biddinger KJ et al. *JAMA Network Open.*

eMethods 9. Allele Score Analyses

Given individual-level data in the UK Biobank, we also constructed externally weighted allele scores for each participant by multiplying the dosage of the allele for increased alcohol consumption by the variant's reported beta coefficient from the Million Veteran Program discovery GWAS and summing across all variants. We then used logistic and linear regression to test for associations with the six aforementioned cardiovascular diseases and a full complement of 31 continuous traits (including those from 2SMR). Regression models were run in current alcohol users and adjusted for age, sex, top 10 principle components of ancestry and genotyping array. Regression models were also run in all subjects to verify associations, and in lifelong abstainers to check for pleiotropy. Statistical significance for the disease and continuous phenotypes were denoted as Bonferroni-corrected threshold of $p < 0.008$ [0.05/6 disease phenotypes] and $p < 0.002$ [0.05/31 traits], respectively.

© 2022 Biddinger KJ et al. *JAMA Network Open.*

eMethods 10. Nonlinear MR

Whereas Mendelian randomization has been employed to assess for a potential causal association in the absence of a randomized controlled trial, traditional approaches often presume linearity.[14,15] A non-linear model may be fit to the data, but – for a non-linear association to be detected – instrumental variables must capture an appreciable portion of the exposure, which genetic instruments often fail to do; consequently, recent studies have employed unique approaches to increase the variance explained by an instrument. For example, to mitigate these limitations, one study utilized a combination of genetics and study area (in conjunction with sex-specific differences in alcohol intake) as proxies for alcohol intake, but still arrived at linear relationships between alcohol intake and cardiovascular disease risk.[16] Here, we applied methods to formally assess for non-linear associations between alcohol intake and cardiovascular disease. To statistically test for non-linear associations – and assess for differential risks at different levels of intake – genetic associations with the outcome may be tested in quantiles of the exposure. However, the genetic instrument itself influences the exposure, subjecting the study to potential collider bias; by extension, stratified allele score analyses should be taken as secondary to more comprehensive non-linear Mendelian randomization. The methods outlined below test genetic associations in conditioned quantiles of residualized exposure (that is, reported alcohol intake minus the influence of the genetic instrument) in order to overcome these limitations and comprehensively assess potential non-linear effects.[14,15,17]

To test the shape of each potential causal association, genetic associations were tested in deciles of residual alcohol intake, a measurement of the exposure devoid of the genetic instrumental variable (IV-free exposure). Specifically, residual alcohol intake was calculated by subtracting genetically predicted alcohol intake from reported alcohol intake (as done previously), and associations were tested in deciles of the residual alcohol intake variable.[17] Residualization was required before partitioning the cohort by amount of alcohol intake in order to avoid overadjustment and collider biases, as the genetic score affects reported alcohol intake. Instead, by stratifying on residual alcohol intake, which defines a participant's alcohol intake if all participants had the same genotype, no such biases are introduced as this variable is unaffected by the genetic score.

Residual alcohol intake was split into deciles – in order to maximize power for stratified analyses – and any outlying values >Q3+1.5*IQR were removed. The association between the genetic score and each cardiovascular outcome was tested within deciles of residual alcohol intake using linear or logistic regression adjusting for genotyping array, sex, age at assessment, and principal components 1-10. In order to standardize effects to a 1 drink per day increase, the decile-specific regression coefficient for the genetic score and the outcome was divided by the regression coefficient for the genetic score and the exposure (alcohol). The resultant association estimate – based on this standardized ratio of coefficients – was referred to as a localized average causal effect (LACE) estimate, as per Staley et al.[14] Interval-specific LACE values were determined and then subsequently used to reconstruct the overall association between alcohol and each tested cardiovascular phenotype using either the "piecewise linear" or "fractional polynomial" method, as described below. In both cases, 0 standard drinks per week was used as the reference value, and abstainers who were former drinkers were excluded as they would be grouped in low categories of intake but could still demonstrate effects of increased alcohol intake. Because all non-linear comparisons are conducted within separate strata of residualized alcohol intake, it is encouraged to focus on the relative slopes of the association rather than absolute risks across the range of the exposure.[13]

For piecewise non-linear MR, the association between alcohol and each outcome – reported as the relative beta coefficient (for continuous traits) or relative odds ratio (for dichotomous traits) – was reconstructed using LACE estimates as the gradient for the corresponding value of reported alcohol intake. LACE values reflect the change in relative beta coefficient or odds ratio for every 1 drink per week increase within each strata of residual alcohol intake. A trend test indicated that exposure associations did not significantly vary in strata of alcohol intake – except for the top and bottom two quantiles – and so a constant exposure association estimate was used in all strata, as in previous studies.[17]

For fractional polynomial non-linear MR, the strata-specific relationships between mean alcohol intake and LACE values were meta-analyzed. Using the standard powers for fitting fractional polynomial models, −2, −1, −0.5, 0 (log function), 0.5, 1, 2, and 3, the best fit models of both degree 1 and 2 were chosen.[14,18] The cutoff for choosing the best fitting model of degree 2 over that of degree 1 was p=0.05, as judged by the maximum likelihood test; however, even best fitting polynomials of degree 2 demonstrated consistently risk increasing estimates for all primary analyses. The association between the exposure (alcohol) and the outcome was then mathematically reconstructed as the derivative of the fractional polynomial model. Code was derived from https://github.com/jrs95/nlmr/tree/master/R, as validated and published previously.[14,17]

This adapted code was also used to calculate several tests to assess fit for disease phenotypes. Three tests of non-linearity were reported: fractional polynomial non-linearity tests, quadratic tests, and Cochran Q tests.[14] The fractional polynomial test involves testing the best fit model against a linear model to determine whether a non-linear model better fits the data. The quadratic test between exposure and outcome is the same as a trend test between exposure and LACE values – i.e. determining if genetic associations vary in different strata of the exposure by meta-regressing LACE values against mean exposure in each strata.A heterogeneity test using Cochran's Q statistic determines whether the difference in LACE values is more than expected by chance. Powers of best-fit disease models (p_1 for models of degree 1, and p_1 and p_2 for models of degree 2) were also recorded with corresponding p-values of fit.

Non-linear MR analyses were performed using the AUD-R allele score as the primary instrument to determine the shape of genetic associations with disease. Secondary analyses were conducted using the AUDIT-C-R allele score as well as a single-SNP instrument comprising the number of alcohol-increasing alleles of rs1229984 at *ADH1B* (a well-established gene for which association with alcohol is known to be mediated by alcohol dehydrogenase). Non-linear MR methods were also used to assess potential causal associations with the ten continuous traits tested with 2SMR techniques, calculating relative beta coefficients rather than odds ratios. Non-linear MR analyses were focused on those disease phenotypes and continuous traits with strong evidence of association with alcohol intake from 2SMR analyses (specifically, Bonferroni significant IVW association, strong weighted median and MR-Egger associations, and no evidence of pleiotropy from MR-Egger intercept or IVW association in lifelong abstainers): hypertension, CAD, systolic and diastolic blood pressure, LDL cholesterol, and gamma glutamyltransferase. Furthermore, as an important sensitivity analysis, we reapplied primary analyses excluding abstainers, as done in previous epidemiological studies.[19] As previously noted, the genetic instrument is not associated with alcohol consumption in this population, because these individuals do not drink alcohol; nonetheless, abstainers have been previously shown to be an overall healthier population than light to moderate drinkers. Thus, in non-linear Mendelian randomization analyses, the genetic instrument may not as accurately reflect the differential alcohol consumption of abstainers vs. light drinkers and therefore non-linear Mendelian randomization results at low levels of intake could be biased by the inclusion of this abstainer population. We also pursued, as a sensitivity analysis, non-linear Mendelian randomization of medication-corrected blood pressure readings, following previously outlined methods.[20]

Lastly, to further assess for residual pleiotropy, and given prior evidence of genetic correlation between alcohol use and smoking initiation, BMI, and depression we conducted multivariable non-linear Mendelian randomization as outlined previously.[21–23] Genetic instruments for BMI and smoking were both derived from GWAS external to the study populations; the genetic instrument for depression was constructed from a meta-analysis that included the UK Biobank, but we included only SNPs that also reached genome-wide significance in externally replication.[24-26] In calculating strata-specific estimates for alcohol use, we adjusted for genetically-proxied BMI and smoking; these estimates were then integrated into non-linearity assessments as outlined above.

© 2022 Biddinger KJ et al. *JAMA Network Open.*

eTable 1. Single-Nucleotide Variants Included in Genetic Instruments

(A) AUD Genetic Instrument

rsid	Locus	Chromosome	Effect_allele	Ref_allele	Effect_freq	Beta_exposure	SE_exposure
rs1260326	GCKR	2	C	T	0.591	0.072	0.009
rs570436	SIX3	2	T	C	0.577	0.056	0.009
rs1229984	ADH1B	4	C	T	0.970	0.555	0.031
rs1154433	ADH1C	4	G	A	0.404	0.069	0.009
rs13107325	SLC39A8	4	C	T	0.921	0.125	0.016
rs4715221	MDFC1	10	A	G	0.698	0.053	0.009
rs17125651	N/A	10	C	T	0.134	0.071	0.012
rs4936277	DRD2	11	A	G	0.560	0.057	0.009
rs11075992	FTO	16	T	C	0.605	0.055	0.009

(B) AUDIT-C Genetic Instrument

rsid	Locus	Chromosome	Effect_allele	Ref_allele	Effect_freq	Beta_exposure	SE_exposure
rs1260326	GCKR	2	C	T	0.591	0.045	0.005
rs2717071	VKR2	2	A	G	0.628	0.034	0.006
rs12639940	KLB	4	A	G	0.614	0.033	0.006
rs1229984	ADH1B	4	C	T	0.970	0.340	0.016
rs1229978	ADH1C	4	C	T	0.403	0.042	0.006
rs13107325	SLC39A8	4	C	T	0.921	0.103	0.010
rs62339861	DCLK2	4	C	T	0.792	0.037	0.007
rs2961817	ISL1	5	A	G	0.657	0.033	0.006
rs185177474	KCTD16	5	A	C	0.031	0.089	0.016
rs12425096	SCN8A	12	A	C	0.775	0.035	0.006
rs9937709	FTO	16	A	G	0.587	0.044	0.005
rs4794018	IGF2BP1	17	C	T	0.337	0.032	0.006
rs35572189	BAHCC1	17	A	G	0.368	0.035	0.006

© 2022 Biddinger KJ et al. *JAMA Network Open.*

eTable 2. Definitions of Cardiovascular Disease Phenotypes in the UK Biobank

Disease	Definition
Hypertension	Self-reported history of hypertension, essential hypertension or high blood pressure during verbal interview with trained nurse; or hospitalization with or death due to ICD-10 code for essential hypertension, hypertensive heart disease, hypertensive renal disease, or secondary hypertension (I10, I11, I12, I13, I15); or hospitalization with ICD-9 code for essential hypertension, hypertensive heart disease, hypertensive renal disease, or secondary hypertension (401, 402, 403, 404, 405)
Coronary artery disease	Self-reported history of myocardial infarction (MI), coronary artery bypass grafting, coronary artery angioplasty or triple heart bypass during verbal interview with trained nurse; or hospitalization with or death due to ICD-10 code for acute or subsequent myocardial infarction (I21, I22, I23, I24.1, I25.2); or hospitalization with ICD-9 code for myocardial infarction (410, 411, 412); or hospitalization with OPCS-4 code for coronary artery bypass grafting (K40, K41, K45) or coronary angioplasty ± stenting (K49, K50.2, K75)
Myocardial infarction	Self-reported history of myocardial infarction (MI) during verbal interview with trained nurse (field 20002, Code 1075); or ICD-9 or ICD-10 code for hospitalization for acute or subsequent myocardial infarction prior to baseline interview (http://biobank.ctsu.ox.ac.uk/crystal/docs/alg_outcome_mi.pdf)
Stroke	Self-reported history of stroke during verbal interview with trained nurse; or hospitalization with or death due to ICD-10 code for nontraumatic subarachnoid hemorrhage, nontraumatic intracerebral hemorrhage, cerebral infarction, or unspecified stroke (I60-64); or hospitalization with or death due to ICD-9 code for subarachnoid hemorrhage, intracerebral hemorrhage, occlusion of cerebral arteries, or acute cerebrovascular disease (430, 431, 434, 436), as adjudicated centrally by the UK Biobank (http://biobank.ctsu.ox.ac.uk/crystal/refer.cgi?id=462)
Heart failure	Self-reported history of heart failure or cardiomyopathy during verbal interview with trained nurse; or hospitalization with or death due to ICD-10 code for hypertensive heart disease, cardiomyopathy or heart failure (I11.0, I13.0, I13.2, I25.5, I42.0, I42.5, I42.8, I42.9, I50.0, I50.1,

© 2022 Biddinger KJ et al. *JAMA Network Open.*

	I50.9); or hospitalization with ICD-9 code for heart failure or other primary cardiomyopathies (4254, 4280, 4281, 4289); excluding individuals with history of hypertrophic cardiomyopathy during verbal interview with trained nurse, or hospitalization with or death due to ICD-10 code for hypertrophic cardiomyopathy (I42.1, I42.2)
Atrial fibrillation	Self-reported history of atrial fibrillation, atrial flutter, or cardioversion during verbal interview with trained nurse; or hospitalization with or death due to ICD-10 code for atrial fibrillation or atrial flutter (I48); or hospitalization with ICD-9 code for atrial fibrillation or atrial flutter (4273); or hospitalization with OPCS-4 code for percutaneous transluminal ablation (K57.1, K 62.1, K62.2, K62.3, K62.4)

© 2022 Biddinger KJ et al. *JAMA Network Open.*

eTable 3. Mean Weekly Standard Measures (1 measure, 14 g) in Drinking Categories and Beverage Composition of Alcohol Consumption

For each drinking category, composition is represented as the percentage of total standard measures of alcohol accounted for by each beverage.

	Mean Weekly Alcohol Consumption		Beer	Red Wine	Champagne /White Wine	Spirits	Fortified Wine	Other
Light Drinkers	4.92		37.72%	29.24%	23.79%	5.94%	2.80%	0.19%
Moderate Drinkers	12.77		38.12%	26.49%	26.33%	6.91%	2.07%	0.07%
Heavy Drinkers	21.15		38.46%	24.47%	28.40%	7.12%	1.51%	0.05%
Abusive Drinkers	37.00		34.69%	21.78%	29.70%	12.33%	1.44%	0.06%

© 2022 Biddinger KJ et al. *JAMA Network Open.*

eTable 4. Assessments for Pleiotropy in Single-Nucleotide Variants in Lifelong Abstainers

(A) AUD Genetic Instrument

SNP	Gene	Smoking	BMI	Phys Act	Vegetable intake	Red meat intake	Overall health rating	C reactive protein	Cholesterol	Included in refined instrument
rs1260326	GCKR	0.156	0.923	0.571	0.145	0.062	0.847	3.11E-36	1.12E-32	No
rs570436	SIX3	2.45E-04	0.783	0.567	0.073	0.081	0.380	0.278	0.540	No
rs1229984	ADH1B	0.729	0.153	0.095	0.783	0.260	0.005	0.455	0.458	Yes
rs1154433	ADH1C	0.955	0.072	0.115	0.708	0.302	0.283	0.116	0.060	Yes
rs13107325	SLC39A8	0.831	7.99E-06	0.697	0.071	0.782	0.183	0.871	0.007	No
rs4715221	MDFC1	0.419	0.695	0.704	0.853	0.025	0.015	0.843	0.538	Yes
rs17125651	N/A	0.517	0.146	0.472	0.270	0.611	0.566	0.872	0.819	Yes
rs4936277	DRD2	0.796	0.758	0.891	0.474	0.975	0.003	0.455	0.021	Yes
rs11075992	FTO	0.426	2.79E-64	0.068	0.058	0.616	0.022	1.69E-06	2.52E-05	No

(B) AUDIT-C Genetic Instrument

SNP	Gene	Smoking	BMI	Phys Act	Vegetable intake	Red meat intake	Overall health rating	C reactive protein	Cholesterol	Included in refined instrument
rs1260326	GCKR	0.156	0.923	0.571	0.145	0.062	0.847	3.11E-36	1.12E-32	No
rs2717071	VKR2	0.099	0.408	0.821	0.548	0.893	0.340	0.129	0.428	Yes
rs12639940	KLB	0.247	0.246	0.095	0.133	0.004	0.218	0.996	0.155	Yes
rs1229984	ADH1B	0.729	0.153	0.095	0.783	0.260	0.005	0.455	0.458	Yes
rs1229978	ADH1C	0.958	0.070	0.115	0.705	0.323	0.270	0.113	0.061	Yes
rs13107325	SLC39A8	0.831	7.99E-06	0.697	0.071	0.782	0.183	0.871	0.007	No
rs62339861	DCLK2	0.051	0.005	0.013	0.846	0.264	0.445	0.761	0.046	Yes
rs2961817	ISL1	0.934	0.615	0.200	0.180	0.099	0.433	0.019	0.235	Yes
rs185177474	KCTD16	0.297	0.934	0.272	0.849	0.120	0.470	0.112	0.278	Yes
rs12425096	SCN8A	0.066	0.383	0.323	0.740	0.286	0.382	0.807	0.463	Yes
rs9937709	FTO	0.557	2.14E-60	0.073	0.073	0.871	0.012	5.62E-06	1.12E-05	No
rs4794018	IGF2BP1	0.012	0.002	0.874	0.189	0.934	0.620	0.246	0.599	Yes
rs35572189	BAHCC1	0.008	0.002	0.127	0.161	0.311	0.367	0.981	0.929	Yes

© 2022 Biddinger KJ et al. *JAMA Network Open.*

eTable 5. Assessments of MR Assumptions in Primary Genetic Instrument

(A) AUD-R allele score associations with traditional alcohol phenotypes

Phenotype	Beta	95% Lower Bound	95% Upper Bound	P-Value	F Statistic
Ln Alcohol	0.824	0.769	0.878	2.34E-169	834.1
Weekly Alcohol	7.000	6.604	7.396	1.39E-174	778.7
Drinking Group	0.748	0.696	0.800	5.18E-174	759.2
**Phenotype**	**Odds Ratio**	**95% Lower Bound Odds**	**95% Upper Bound Odds**	**P-Value**	
Drinker	2.740	2.454	3.062	3.16E-71	
Overlimits	4.272	3.856	4.733	6.70E-170	

(B) AUD-R allele score associations with individual alcoholic beverages

	Beta Coefficient	95% Lower Bound	95% Upper Bound	P-value
Weekly Alcohol	7.000	6.604	7.396	1.39E-174
Weekly Beer	1.835	1.622	2.049	1.16E-63
Weekly Red Wine	2.130	1.890	2.370	7.56E-68
Weekly Champagne/White Wine	1.449	1.250	1.647	2.65E-46
Weekly Spirits	0.838	0.625	1.050	1.17E-14
Weekly Fortified Wine	0.138	0.087	0.190	1.18E-07

(C) AUD-R allele score associations with possible confounders (tested in lifelong abstainers only)

	Beta Coefficient	95% Lower Bound	95% Upper Bound	P-value
Smoking	-0.085	-0.170	0.001	0.052
BMI	0.269	-0.481	1.018	0.482
Phys Act	0.308	-0.054	0.670	0.095
Vegetable intake	0.020	-0.330	0.371	0.909
Red meat intake	-0.150	-0.398	0.097	0.234
Overall health rating	0.078	-0.029	0.185	0.154

© 2022 Biddinger KJ et al. *JAMA Network Open.*

eTable 6. Assessments of MR Assumptions in Secondary Genetic Instruments

(A) AUDIT-C-R allele score associations with traditional alcohol phenotypes

**Phenotype**	**Beta**	**95% Lower Bound**	**95% Upper Bound**	**P-Value**	**F Statistic**
Ln Alcohol	0.817	0.769	0.865	1.31E-213	1012
Weekly Alcohol	7.000	6.569	7.431	1.06E-221	955.2
Drinking Group	0.739	0.693	0.786	3.30E-216	911.5
**Phenotype**	**Odds Ratio**	**95% Lower Bound Odds**	**95% Upper Bound Odds**	**P-Value**	
Drinker	2.829	2.562	3.122	1.73E-94	
Overlimits	4.005	3.661	4.383	1.09E-200	

(B) AUDIT-C-R allele score associations with individual alcoholic beverages

	**Beta Coefficient**	**95% Lower Bound**	**95% Upper Bound**	**P-value**
Weekly Alcohol	7.000	6.569	7.431	1.06E-221
Weekly Beer	1.870	1.681	2.060	1.43E-83
Weekly Red Wine	2.081	1.869	2.294	4.74E-82
Weekly Champagne/White Wine	1.463	1.287	1.639	1.40E-59
Weekly Spirits	0.911	0.723	1.100	2.67E-21
Weekly Fortified Wine	0.101	0.056	0.146	1.32E-05

(C) AUDIT-C-R allele score associations with possible confounders (tested in lifelong abstainers only)

	**Beta Coefficient**	**95% Lower Bound**	**95% Upper Bound**	**P-value**
Smoking	-0.029	-0.106	0.049	0.467
BMI	-0.089	-0.770	0.591	0.797
Phys Act	0.248	-0.081	0.576	0.140
Vegetable intake	-0.043	-0.361	0.275	0.790
Red meat intake	-0.201	-0.426	0.024	0.079
Overall health rating	0.048	-0.050	0.145	0.337

(D) AUD allele score associations with traditional alcohol phenotypes

**Phenotype**	**Beta**	**95% Lower Bound**	**95% Upper Bound**	**P-Value**	**F Statistic**
Ln Alcohol	0.806	0.762	0.851	2.84E-244	1157
Weekly Alcohol	7.000	6.604	7.396	5.60E-262	1113
Drinking	0.742	0.700	0.784	3.18E-257	1073

© 2022 Biddinger KJ et al. *JAMA Network Open.*

Group					
**Phenotype**	**Odds Ratio**	**95% Lower Bound Odds**	**95% Upper Bound Odds**	**P-Value**	
Drinker	2.759	2.519	3.022	6.89E-106	
Overlimits	4.000	3.683	4.345	1.55E-236	

(E) AUD allele score associations with individual alcoholic beverages

	**Beta Coefficient**	**95% Lower Bound**	**95% Upper Bound**	**P-value**
Weekly Alcohol	7.000	6.604	7.396	5.60E-262
Weekly Beer	1.981	1.807	2.155	2.64E-110
Weekly Red Wine	2.054	1.859	2.250	2.61E-94
Weekly Champagne/White Wine	1.466	1.304	1.628	1.90E-70
Weekly Spirits	0.715	0.542	0.889	5.93E-16
Weekly Fortified Wine	0.105	0.064	0.147	7.70E-07

(F) AUD allele score associations with possible confounders (tested in lifelong abstainers only)

	**Beta Coefficient**	**95% Lower Bound**	**95% Upper Bound**	***P*-value**
Smoking	-0.068	-0.140	0.003	0.062
BMI	-0.802	-1.432	-0.172	0.013
Phys Act	0.186	-0.118	0.490	0.231
Vegetable intake	-0.142	-0.436	0.152	0.344
Red meat intake	-0.146	-0.354	0.062	0.169
Overall health rating	0.019	-0.071	0.109	0.675

(G) AUDIT-C allele score associations with traditional alcohol phenotypes

**Phenotype**	**Beta**	**95% Lower Bound**	**95% Upper Bound**	***P*-Value**	**F Statistic**
Ln Alcohol	0.806	0.764	0.848	1.02E-268	1240
Weekly Alcohol	7.000	6.621	7.379	2.32E-286	1193
Drinking Group	0.736	0.695	0.776	1.31E-276	1130

**Phenotype**	**Odds Ratio**	**95% Lower Bound Odds**	**95% Upper Bound Odds**	***P*-Value**
Drinker	2.838	2.601	3.098	8.88E-121
Overlimits	3.820	3.532	4.132	3.25E-245

(H) AUDIT-C allele score associations with individual alcoholic beverages

	Beta Coefficient	95% Lower Bound	95% Upper Bound	*P*-value
Weekly Alcohol	7.000	6.621	7.379	2.32E-286
Weekly Beer	2.001	1.834	2.167	7.70E-123
Weekly Red Wine	1.968	1.781	2.155	1.21E-94
Weekly Champagne/White Wine	1.523	1.368	1.678	7.85E-83
Weekly Spirits	0.766	0.600	0.931	1.36E-19
Weekly Fortified Wine	0.093	0.053	0.133	5.52E-06

(I) AUDIT-C allele score associations with possible confounders (tested in lifelong abstainers only)

	Beta Coefficient	95% Lower Bound	95% Upper Bound	*P*-value
Smoking	-0.048	-0.118	0.022	0.176
BMI	-1.193	-1.805	-0.581	1.33E-04
Phys Act	0.136	-0.159	0.431	0.366
Vegetable intake	-0.142	-0.428	0.144	0.330
Red meat intake	-0.147	-0.349	0.055	0.155
Overall health rating	-0.002	-0.089	0.086	0.968

© 2022 Biddinger KJ et al. *JAMA Network Open.*

eTable 7. Primary Genetic Associations Between Alcohol and Continuous Traits

Outcome statistics were derived in current drinkers unless otherwise specified.

(A) 2SMR Associations using AUD-R genetic instrument

	**Current Drinkers IVW Odds Ratio (*P*-value)**	**Current Drinkers Weighted Median Odds Ratio (*P*-value)**	**Current Drinkers MR Egger Odds Ratio (*P*-value)**	**Current Drinkers MR Egger Intercept (*P*-value)**	**Lifelong Abstainers IVW Odds Ratio (*P*-value)**
SBP	0.133 (3.03E-14)	0.141 (2.37E-15)	0.151 (1.32E-10)	-0.003 (0.264)	0.052 (0.058)
DBP	0.096 (4.39E-04)	0.091 (3.56E-07)	0.064 (0.064)	0.005 (0.182)	0.034 (0.175)
HDL Cholesterol	-0.048 (0.091)	-0.042 (0.009)	-0.047 (0.329)	-1.09E-04 (0.983)	-0.061 (0.022)
Apolipoprotein A	-0.075 (0.036)	-0.072 (1.70E-05)	-0.089 (0.114)	0.002 (0.725)	-0.086 (0.001)
LDL Cholesterol	0.151 (4.44E-16)	0.165 (1.36E-04)	0.205 (5.28E-06)	-0.008 (0.085)	-0.018 (0.553)
Apolipoprotein B	0.084 (0.010)	0.086 (9.75E-07)	0.105 (0.033)	-0.003 (0.547)	-0.041 (0.258)
Triglycerides	-0.006 (0.794)	-0.012 (0.472)	-0.018 (0.602)	0.002 (0.611)	0.025 (0.280)
Total Cholesterol	0.103 (2.88E-06)	0.117 (2.22E-11)	0.138 (4.40E-12)	-0.005 (0.010)	-0.041 (0.224)
Gamma glutamyl transferase	0.087 (9.56E-05)	0.072 (3.22E-05)	0.067 (0.034)	0.003 (0.366)	0.034 (0.170)
C reactive protein	0.085 (0.011)	0.084 (1.90E-06)	0.081 (0.132)	0.001 (0.901)	-0.006 (0.788)

(B) Allele score associations using AUD-R genetic instrument

	**Current Drinkers Beta [95% CI]**	**Current Drinkers *P*-Value**	**Abstain NFD Beta [95% CI]**	**Abstain NFD *P*-Value**
Alanine aminotransferase	0.043 [-0.017; 0.103]	0.159	-0.197 [-0.343; -0.051]	0.008
Albumin	0.114 [0.055; 0.173]	1.52E-04	-0.141 [-0.294; 0.012]	0.071
Alkaline phosphatase	0.288 [0.228; 0.348]	4.23E-21	0.108 [-0.036; 0.251]	0.143
Apolipoprotein A	-0.152 [-0.209; -0.094]	2.28E-07	-0.159 [-0.32; 0.001]	0.052
Apolipoprotein B	0.171 [0.111; 0.23]	1.99E-08	-0.089 [-0.239; 0.06]	0.241
Aspartate aminotransferase	0.053 [-0.007; 0.112]	0.082	-0.046 [-0.193; 0.1]	0.536
C reactive protein	0.173 [0.113; 0.232]	1.46E-08	-0.093 [-0.241; 0.055]	0.218
Calcium	0.122 [0.062; 0.181]	6.05E-05	-0.097 [-0.248; 0.055]	0.210
Cholesterol	0.208 [0.149; 0.267]	4.83E-12	-0.085 [-0.237; 0.067]	0.274
Creatinine	-0.147 [-0.207; -0.088]	1.21E-06	-0.122 [-0.267; 0.023]	0.099
Cystatin C	0.100 [0.04; 0.16]	0.001	0.030 [-0.114; 0.174]	0.682
Direct bilirubin	0.058 [0; 0.117]	0.049	0.028 [-0.123; 0.179]	0.716
Gamma glutamyltransferase	0.176 [0.116; 0.235]	7.28E-09	-0.018 [-0.168; 0.132]	0.817

© 2022 Biddinger KJ et al. *JAMA Network Open.*

HDL Cholesterol	-0.097 [-0.154; -0.039]	0.001	-0.142 [-0.305; 0.021]	0.088
Insulin-like growth factor 1	-0.338 [-0.397; -0.279]	2.24E-29	-0.188 [-0.34; -0.035]	0.016
LDL Cholesterol	0.306 [0.247; 0.366]	8.36E-24	-0.054 [-0.202; 0.094]	0.472
Lipoprotein A	-0.022 [-0.083; 0.038]	0.467	0.009 [-0.14; 0.159]	0.904
Oestradiol	-0.016 [-0.073; 0.042]	0.597	-0.004 [-0.146; 0.138]	0.955
Phosphate	0.074 [0.015; 0.134]	0.014	-0.033 [-0.185; 0.119]	0.673
Rheumatoid factor	0.028 [-0.032; 0.088]	0.362	0 [-0.149; 0.148]	0.998
SHBG	-0.112 [-0.171; -0.053]	1.94E-04	0.013 [-0.141; 0.167]	0.870
Testosterone	0.009 [-0.049; 0.067]	0.759	-0.007 [-0.165; 0.151]	0.929
Total bilirubin	0.072 [0.014; 0.131]	0.016	-0.095 [-0.246; 0.057]	0.221
Total protein	0.189 [0.129; 0.248]	5.00E-10	0.001 [-0.147; 0.15]	0.985
Triglycerides	-0.012 [-0.072; 0.049]	0.703	0.045 [-0.097; 0.187]	0.532
Urate	-0.185 [-0.245; -0.126]	1.02E-09	-0.146 [-0.296; 0.005]	0.057
Urea	-0.211 [-0.271; -0.152]	3.53E-12	-0.068 [-0.216; 0.079]	0.363
Vitamin D	-0.086 [-0.146; -0.027]	0.004	-0.143 [-0.301; 0.015]	0.077
BMI	0.236 [0.179; 0.293]	5.21E-16	-0.028 [-0.19; 0.135]	0.740
SBP	0.269 [0.21; 0.329]	7.31E-19	0.099 [-0.055; 0.253]	0.206
DBP	0.194 [0.135; 0.253]	1.38E-10	0.056 [-0.096; 0.208]	0.472

© 2022 Biddinger KJ et al. *JAMA Network Open.*

eTable 8. Secondary Genetic Associations Between Alcohol and Continuous Traits

Outcome statistics were derived in current drinkers unless otherwise specified.

(A) 2SMR Associations using AUDIT-C-R genetic instrument

	Current Drinkers IVW Odds Ratio (*P*-value)	Current Drinkers Weighted Median Odds Ratio (*P*-value)	Current Drinkers MR Egger Odds Ratio (*P*-value)	Current Drinkers MR Egger Intercept (*P*-value)	Lifelong Abstainers IVW Odds Ratio (*P*-value)
SBP	0.149 (0.022)	0.241 (3.31E-16)	0.272 (0.003)	-0.008 (0.077)	0.012 (0.811)
DBP	0.124 (0.019)	0.121 (1.10E-05)	0.149 (0.085)	-0.002 (0.708)	-0.018 (0.715)
HDL Cholesterol	-0.038 (0.310)	-0.062 (0.017)	-0.096 (0.089)	0.004 (0.182)	-0.063 (0.172)
Apolipoprotein A	-0.069 (0.135)	-0.077 (0.011)	-0.160 (0.012)	0.006 (0.061)	-0.090 (0.068)
LDL Cholesterol	0.191 (4.40E-04)	0.246 (3.25E-10)	0.323 (1.16E-06)	-0.009 (0.010)	0.002 (0.966)
Apolipoprotein B	0.112 (0.003)	0.132 (2.67E-06)	0.167 (0.003)	-0.004 (0.213)	-0.014 (0.741)
Triglycerides	0.021 (0.599)	-0.021 (0.454)	-0.040 (0.509)	0.004 (0.188)	0.044 (0.225)
Total Cholesterol	0.144 (1.78E-05)	0.172 (3.75E-09)	0.213 (2.56E-06)	-0.005 (0.046)	-0.020 (0.652)
Gamma glutamyl transferase	0.156 (0.031)	0.116 (2.92E-05)	0.097 (0.404)	0.004 (0.511)	0.045 (0.204)
C reactive protein	0.105 (0.026)	0.121 (7.52E-06)	0.136 (0.076)	-0.002 (0.595)	-0.016 (0.712)

(B) Allele score associations using AUDIT-C-R genetic instrument

	Current Drinkers Beta (95% CI)	Current Drinkers *P*-Value	Abstain NFD Beta (95% CI)	Abstain NFD *P*-Value
Alanine aminotransferase	0.073 [0.021; 0.126]	0.006	-0.154 [-0.287; -0.022]	0.023
Albumin	0.106 [0.054; 0.158]	6.93E-05	-0.136 [-0.275; 0.004]	0.056
Alkaline phosphatase	0.242 [0.189; 0.295]	2.52E-19	0.126 [-0.005; 0.257]	0.059
Apolipoprotein A	-0.088 [-0.139; -0.038]	6.30E-04	-0.142 [-0.288; 0.004]	0.057
Apolipoprotein B	0.149 [0.096; 0.201]	3.08E-08	-0.062 [-0.198; 0.074]	0.373
Aspartate	0.041 [-0.011; 0.094]	0.124	-0.030 [-0.163;	0.658

© 2022 Biddinger KJ et al. *JAMA Network Open.*

aminotransferase			0.103]	
C reactive protein	0.140 [0.088; 0.193]	1.77E-07	-0.047 [-0.181; 0.088]	0.497
Calcium	0.118 [0.065; 0.17]	1.05E-05	-0.099 [-0.236; 0.039]	0.160
Cholesterol	0.192 [0.14; 0.244]	4.73E-13	-0.090 [-0.228; 0.048]	0.201
Creatinine	-0.083 [-0.135; -0.03]	0.002	-0.067 [-0.199; 0.065]	0.321
Cystatin C	0.114 [0.061; 0.167]	2.31E-05	0.049 [-0.082; 0.18]	0.467
Direct bilirubin	0.069 [0.018; 0.121]	0.008	0.053 [-0.084; 0.19]	0.451
Gamma glutamyltransferase	0.210 [0.158; 0.263]	4.46E-15	-0.004 [-0.14; 0.133]	0.957
HDL Cholesterol	-0.046 [-0.097; 0.004]	0.072	-0.126 [-0.274; 0.023]	0.098
Insulin-like growth factor 1	-0.218 [-0.27; -0.166]	1.77E-16	-0.154 [-0.293; -0.016]	0.029
LDL Cholesterol	0.256 [0.203; 0.308]	1.75E-21	-0.062 [-0.197; 0.073]	0.367
Lipoprotein A	0.011 [-0.042; 0.064]	0.693	-0.062 [-0.198; 0.074]	0.372
Oestradiol	-0.043 [-0.093; 0.008]	0.101	0.031 [-0.098; 0.16]	0.639
Phosphate	0.088 [0.035; 0.14]	0.001	-0.050 [-0.188; 0.088]	0.477
Rheumatoid factor	-0.030 [-0.083; 0.023]	0.272	0.047 [-0.088; 0.182]	0.499
SHBG	-0.060 [-0.112; -0.008]	0.025	0.007 [-0.133; 0.148]	0.917
Testosterone	-0.027 [-0.079; 0.024]	0.295	0.068 [-0.075; 0.212]	0.351
Total bilirubin	0.068 [0.016; 0.12]	0.010	-0.118 [-0.256; 0.02]	0.093
Total protein	0.149 [0.097; 0.202]	2.55E-08	-0.014 [-0.149; 0.121]	0.835
Triglycerides	0.032 [-0.021; 0.085]	0.239	-0.010 [-0.139; 0.119]	0.880
Urate	-0.110 [-0.162; -0.057]	4.24E-05	-0.106 [-0.243; 0.031]	0.128
Urea	-0.187 [-0.239; -0.134]	3.63E-12	-0.107 [-0.241; 0.027]	0.117
Vitamin D	-0.083 [-0.136; -0.031]	0.002	-0.106 [-0.249; 0.038]	0.149
BMI	0.136 [0.085; 0.186]	1.32E-07	-0.108 [-0.256; 0.04]	0.152
SBP	0.184 [0.131; 0.236]	6.40E-12	0.037 [-0.103; 0.177]	0.607
DBP	0.142 [0.09; 0.194]	1.00E-07	0.017 [-0.121; 0.155]	0.805

© 2022 Biddinger KJ et al. *JAMA Network Open.*

eTable 9. Secondary Genetic Associations Between Alcohol and Cardiovascular Disease Phenotypes

(A) 2SMR Associations using AUD-R genetic instrument

	Current Drinkers IVW Odds Ratio (*P*-value)	Current Drinkers Weighted Median Odds Ratio (*P*-value)	Current Drinkers MR Egger Odds Ratio (*P*-value)	Current Drinkers MR Egger Intercept (*P*-value)	Lifelong Abstainers IVW Odds Ratio (*P*-value)
Hypertension	1.28 (1.73E-09)	1.31 (1.06E-12)	1.31 (4.79E-05)	0.00 (0.724)	1.17 (0.050)
Coronary artery disease	1.38 (0.006)	1.47 (2.17E-08)	1.54 (0.014)	-0.02 (0.394)	1.20 (0.126)
Myocardial infarction	1.37 (0.020)	1.52 (8.53E-06)	1.61 (0.007)	-0.02 (0.188)	1.30 (0.192)
Stroke	1.26 (0.021)	1.28 (0.030)	1.34 (0.040)	-0.01 (0.250)	1.21 (0.572)
Heart failure	1.39 (0.009)	1.50 (0.004)	0.47 (0.010)	-0.02 (0.280)	0.67 (0.096)
Atrial fibrillation	1.24 (0.003)	1.25 (0.009)	1.23 (0.050)	0.00 (0.860)	0.94 (0.811)

(B) 2SMR Associations using AUDIT-C-R genetic instrument

	Current Drinkers IVW Odds Ratio (*P*-value)	Current Drinkers Weighted Median Odds Ratio (*P*-value)	Current Drinkers MR Egger Odds Ratio (*P*-value)	Current Drinkers MR Egger Intercept (*P*-value)	Lifelong Abstainers IVW Odds Ratio (*P*-value)
Hypertension	1.247 (0.061)	1.438 (1.08E-07)	1.634 (0.001)	-0.018 (0.022)	1.048 (0.758)
Coronary artery disease	1.422 (0.014)	1.655 (4.09E-05)	2.010 (2.39E-04)	-0.022 (0.020)	1.140 (0.505)
Myocardial infarction	1.319 (0.159)	1.941 (5.28E-05)	2.203 (0.001)	-0.032 (0.008)	1.294 (0.369)
Stroke	1.315 (0.062)	1.490 (0.027)	1.690 (0.018)	-0.016 (0.150)	1.171 (0.396)
Heart failure	1.234 (0.415)	1.822 (0.011)	2.300 (0.017)	-0.039 (0.023)	0.770 (0.535)
Atrial fibrillation	1.265 (0.023)	1.392 (0.013)	1.480 (0.016)	-0.010 (0.213)	0.792 (0.367)

(C) 2SMR Associations using AUD genetic instrument

	Current Drinkers IVW Odds Ratio (*P*-value)	Current Drinkers Weighted Median Odds Ratio (*P*-value)	Current Drinkers MR Egger Odds Ratio (*P*-value)	Current Drinkers MR Egger Intercept (*P*-value)	Lifelong Abstainers IVW Odds Ratio (*P*-value)
Hypertension	1.230 (0.027)	1.287 (7.93E-12)	1.402 (0.022)	-0.015 (0.255)	1.047 (0.679)
Coronary artery	1.262 (0.023)	1.487 (9.72E-09)	1.547 (0.093)	-0.022 (0.093)	1.225 (0.138)

© 2022 Biddinger KJ et al. *JAMA Network Open.*

disease					
Myocardial infarction	1.228 (0.041)	1.413 (0.001)	1.567 (0.001)	-0.026 (0.024)	1.126 (0.461)
Stroke	1.194 (0.035)	1.294 (0.015)	1.370 (0.022)	-0.015 (0.205)	1.209 (0.396)
Heart failure	1.257 (0.027)	1.441 (0.006)	1.496 (0.020)	-0.019 (0.212)	0.606 (0.021)
Atrial fibrillation	1.172 (0.011)	1.227 (0.010)	1.208 (0.061)	-0.003 (0.704)	0.925 (0.658)

(D) 2SMR Associations using AUDIT-C genetic instrument

	**Current Drinkers IVW Odds Ratio (*P*-value)**	**Current Drinkers Weighted Median Odds Ratio (*P*-value)**	**Current Drinkers MR Egger Odds Ratio (*P*-value)**	**Current Drinkers MR Egger Intercept (*P*-value)**	**Lifelong Abstainers IVW Odds Ratio (*P*-value)**
Hypertension	1.226 (0.117)	1.423 (2.36E-07)	1.798 (0.001)	-0.025 (0.010)	0.958 (0.772)
Coronary artery disease	1.306 (0.042)	1.570 (1.99E-04)	1.978 (2.43E-04)	-0.026 (0.007)	1.047 (0.789)
Myocardial infarction	1.198 (0.241)	1.132 (0.449)	2.012 (0.001)	-0.032 (0.003)	1.087 (0.726)
Stroke	1.247 (0.061)	1.380 (0.040)	1.662 (0.014)	-0.018 (0.091)	1.110 (0.523)
Heart failure	1.165 (0.437)	1.355 (0.159)	1.937 (0.035)	-0.031 (0.050)	0.551 (0.115)
Atrial fibrillation	1.170 (0.076)	1.106 (0.409)	1.377 (0.036)	-0.010 (0.188)	0.739 (0.187)

© 2022 Biddinger KJ et al. *JAMA Network Open.*

eTable 10. MR-PRESSO Sensitivity Analyses for 2SMR Analyses

The Global Test RSS_{obs} (residual sum of squares) measures the amount of residual variance, with a lower value indicating a better fit of the genetic model; a low Global Test p-value indicates the presence of horizontal pleiotropy. If one or more SNPs were significant outliers, a new outlier-corrected OR is (if there was influence of horizontal pleiotropy/outliers) the new OR/p-val excluding outliers. The distortion test assesses whether the original and outlier-corrected models are significantly different.

(A) AUD-R Associations with CVD phenotypes

	**Global Test RSS_{obs}**	**Global Test *P*-value**	**Number of Detected Outliers**	**Outlier-corrected OR (*P*-value)**	**Distortion Test *P*-value**
Hypertension	10.17224	0.285	0	NA	NA
CAD	28.52291	0.019	1	1.467 (0.004)	0.409
MI	23.54193	0.024	0	NA	NA
HF	6.277019	0.456	0	NA	NA
Stroke	1.661363	0.884	0	NA	NA
AF	0.8754201	0.962	0	NA	NA

(B) AUD-R Associations with continuous traits

	**Global Test RSS_{obs}**	**Global Test *P*-value**	**Number of Detected Outliers**	**Outlier-corrected Beta (*P*-value)**	**Distortion Test *P*-value**
SBP	12.870	0.196	0	NA	NA
DBP	30.372	0.012	1	0.021 (0.031)	0.324
HDL Cholesterol	23.115	0.021	1	-0.029 (0.237)	<0.001
Apolipoprotein A	32.605	0.006	1	-0.055 (0.148)	0.066
LDL Cholesterol	67.018	0.001	1	0.167 (0.020)	0.62
Apolipoprotein B	27.169	0.020	1	0.068 (0.101)	0.259
Triglycerides	14.214	0.134	0	NA	NA
Total Cholesterol	27.343	0.018	0	NA	NA
Gamma glutamyl transferase	17.148	0.065	0	NA	NA
C reactive protein	26.632	0.019	1	0.066 (0.087)	0.241

(C) AUDIT-C-R Associations with CVD phenotypes

	**Global Test RSS_{obs}**	**Global Test *P*-value**	**Number of Detected Outliers**	**Outlier-corrected OR (*P*-value)**	**Distortion Test *P*-value**
Hypertension	106.788	<0.001	3	1.214 (0.038)	0.203
CAD	49.077	<0.001	2	1.188 (0.253)	<0.001
MI	48.779	0.001	2	1.047 (0.813)	<0.001
HF	30.072	0.012	1	1.406 (0.116)	0.672
Stroke	12.884	0.329	0	NA	NA
AF	11.251	0.438	0	NA	NA

© 2022 Biddinger KJ et al. *JAMA Network Open.*

(D) AUDIT-C-R Associations with continuous traits

	**Global Test RSS_{obs}**	**Global Test *P*-value**	**Number of Detected Outliers**	**Outlier-corrected Beta (*P*-value)**	**Distortion Test *P*-value**
SBP	131.176	<0.001	4	0.130 (0.357)	0.025
DBP	69.845	<0.001	2	0.133 (0.004)	0.677
HDL Cholesterol	42.911	<0.001	1	-0.012 (0.678)	0.254
Apolipoprotein A	70.688	<0.001	1	-0.040 (0.325)	0.381
LDL Cholesterol	113.503	<0.001	2	0.118 (0.051)	<0.001
Apolipoprotein B	41.035	0.001	2	0.105 (0.007)	0.787
Triglycerides	46.151	<0.001	1	0.004 (0.899)	0.139
Total Cholesterol	41.642	0.002	1	0.159 (<0.001)	0.709
Gamma glutamyl transferase	133.577	<0.001	4	0.125 (0.025)	0.448
C reactive protein	57.111	<0.001	2	0.102 (0.010)	0.890

© 2022 Biddinger KJ et al. *JAMA Network Open.*

eTable 11. Sex-Stratified Allele Score Associations Between Alcohol and Primary Cardiovascular Disease Outcomes

(A) AUD-R genetic instrument

	Light Drinkers (57690 men, 90463 women)	Moderate Drinkers (41277 men, 33672 women)	Heavy Drinkers (26595 men, 12971 women)	Abusive Drinkers (18919 men, 4865 women)
Hypertension, Men	1.39 (95% CI, 1.08-1.78, p=0.010)	1.60 (95% CI, 1.16-2.20, p=0.004)	1.92 (95% CI, 1.27-2.91, p=0.002)	2.80 (95% CI, 1.65-4.74, p<0.001)
Hypertension, Women	1.22 (95% CI, 0.97-1.53, p=0.093)	2.01 (95% CI, 1.30-3.11, p=0.002)	1.78 (95% CI, 0.87-3.65, p=0.113)	1.71 (95% CI, 0.54-5.41, p=0.364)
Coronary Artery Disease, Men	1.74 (95% CI, 1.19-2.55, p=0.004)	1.75 (95% CI, 1.07-2.87, p=0.027)	2.25 (95% CI, 1.14-4.44, p=0.019)	6.60 (95% CI, 2.68-16.28, p<0.001)
Coronary Artery Disease, Women	1.66 (95% CI, 0.92-3.01, p=0.093)	1.74 (95% CI, 0.53-5.66, p=0.359)	1.10 (95% CI, 0.15-8.04, p=0.927)	1.00 (95% CI, 0.05-18.42, p=0.998)

(B) AUDIT-C-R genetic instrument

	Light Drinkers (57690 men, 90463 women)	Moderate Drinkers (41277 men, 33672 women)	Heavy Drinkers (26595 men, 12971 women)	Abusive Drinkers (18919 men, 4865 women)
Hypertension, Men	1.27 (95% CI, 1.01-1.59, p=0.037)	1.32 (95% CI, 1.00-1.75, p=0.053)	1.47 (95% CI, 1.02-2.10, p=0.038)	1.78 (95% CI, 1.13-2.78, p=0.012)
Hypertension, Women	1.05 (95% CI, 0.85-1.28, p=0.667)	1.24 (95% CI, 0.86-1.79, p=0.244)	0.83 (95% CI, 0.46-1.51, p=0.548)	1.48 (95% CI, 0.56-3.93, p=0.430)
Coronary Artery Disease, Men	1.63 (95% CI, 1.16-2.29, p=0.005)	1.55 (95% CI, 1.01-2.39, p=0.046)	2.19 (95% CI, 1.22-3.93, p=0.008)	2.65 (95% CI, 1.28-5.50, p=0.009)
Coronary Artery Disease, Women	1.40 (95% CI, 0.83-2.34, p=0.204)	1.52 (95% CI, 0.56-4.12, p=0.406)	1.35 (95% CI, 0.25-7.28, p=0.727)	0.17 (95% CI, 0.02-1.69, p=0.131)

© 2022 Biddinger KJ et al. *JAMA Network Open.*

eTable 12. Nonlinear MR Tests

A low fractional polynomial non-linearity p-value indicates a non-linear relationship better fits the relationship than a linear model, and a low quadratic test p-value indicates a non-linear relationship between the exposure and the outcome. The Cochran Q p-value tests if LACE values differ more than would be expected by chance. Fractional polynomial powers (p_1 for models of degree 1 and p_1 and p_2 for models of degree 2) are also listed with respective p-values.

(A) Disease phenotypes, testing using AUD-R as genetic instrument

	**Fractional polynomial non-linearity *P*-value**	**Quadratic *P*-value**	**Cochran Q *P*-value**	**p_1 (*P*-value)**	**p_2 (*P*-value)**
Hypertension	1.31E-05	7.14E-05	3.07E-05	2 (4.93E-09)	NA
CAD	9.24E-04	8.36E-04	0.002	2 (4.56E-06)	NA
MI	0.004	0.003	7.83E-04	2 (1.65E-04)	NA
Stroke	0.216	0.162	0.660	3 (0.175)	NA
Heart Failure	0.053	0.029	0.013	3 (0.032)	NA
Atrial Fibrillation	0.491	0.384	0.604	2 (0.044)	NA

(B) Continuous traits, testing using AUD-R as genetic instrument

	**Fractional polynomial non-linearity *P*-value**	**Quadratic *P*-value**	**Cochran Q *P*-value**	**p_1 (*P*-value)**	**p_2 (*P*-value)**
GGT	0.001	0.001	0.038	2 (2.35E-07)	NA
LDL-C	6.32E-10	5.92E-10	3.36E-06	2 (1.79E-22)	NA
SBP	0.035	0.003	0.267	2 (1.16E-14)	NA
DBP	0.130	0.109	0.649	2 (0.001)	NA

(C) Disease phenotypes, testing using AUDIT-C-R as genetic instrument

	**Fractional polynomial non-linearity *P*-value**	**Quadratic *P*-value**	**Cochran Q *P*-value**	**p_1 (*P*-value)**	**p_2 (*P*-value)**
Hypertension	8.86E-04	5.45E-06	6.17E-05	1 (9.91E-06)	log 1 (1.71E-06)
CAD	0.002	0.001	0.002	2 (8.08E-05)	NA
MI	0.010	0.005	0.003	2 (0.003)	NA
Stroke	0.304	0.184	0.224	3 (0.3035)	NA
Heart Failure	0.209	0.174	0.002	2 (0.147)	NA
Atrial Fibrillation	0.129	0.105	0.591	2 (0.065)	NA

(D) Continuous traits, testing using AUDIT-C-R as genetic instrument

	**Fractional polynomial non-linearity *P*-**	**Quadratic *P*-value**	**Cochran Q *P*-value**	**p_1 (*P*-value)**	**p_2 (*P*-value)**

© 2022 Biddinger KJ et al. *JAMA Network Open.*

	**value**				
GGT	0.003	0.002	0.001	2 (4.31E-10)	NA
LDL-C	7.46E-05	2.96E-05	0.017	2 (7.55E-18)	NA
SBP	0.014	0.006	0.223	2 (1.42E-09)	NA
DBP	0.037	0.023	0.328	2 (0.011)	NA

© 2022 Biddinger KJ et al. *JAMA Network Open.*

eTable 13. Study Characteristics in Mass General Brigham Biobank

Table. Baseline Characteristics of Individuals in the Mass General Brigham Biobank	
No. Individuals	30716
Age, mean (SD)	57.23 (17.26)
Men, No. (%)	13935 (45.37)
Weekly Alcohol Consumption, Mean (SD)	3.52 (5.83)
Blood Pressure, Mean (SD), mmHg	
Systolic	150.00 (21.64)
Diastolic	89.45 (11.51)

© 2022 Biddinger KJ et al. *JAMA Network Open.*

eTable 14. Associations of AUD-R Allele Score With Alcohol Consumption and Blood Pressure Measurements in Mass General Brigham Biobank

(A) Allele score associations using linear regression.

	Beta Coefficient	95% Lower Bound	95% Upper Bound	P-value
Weekly Alcohol	7.000	5.149	8.851	1.32E-13
Systolic Blood Pressure	3.597	-0.923	8.117	0.119
Diastolic Blood Pressure	3.507	0.848	6.166	0.010

(B) Non-linearity tests. A low fractional polynomial non-linearity p-value indicates a non-linear relationship better fits the relationship than a linear model, and a low quadratic test p-value indicates a non-linear relationship between the exposure and the outcome. The Cochran Q p-value tests if LACE values differ more than would be expected by chance. Fractional polynomial powers (p_1 for models of degree 1 and p_1 and p_2 for models of degree 2) are also listed with respective p-values.

	**Fractional polynomial non-linearity *P*-value**	**Quadratic *P*-value**	**Cochran Q *P*-value**	**p_1 (*P*-value)**	**p_2 (*P*-value)**
Systolic Blood Pressure	0.304	0.297	0.042	2 (0.068)	NA
Diastolic Blood Pressure	0.006	0.003	0.001	2 (0.001)	NA

© 2022 Biddinger KJ et al. *JAMA Network Open.*

eFigure 1. Alcohol Consumption and Prevalence of Cardiovascular Diseases

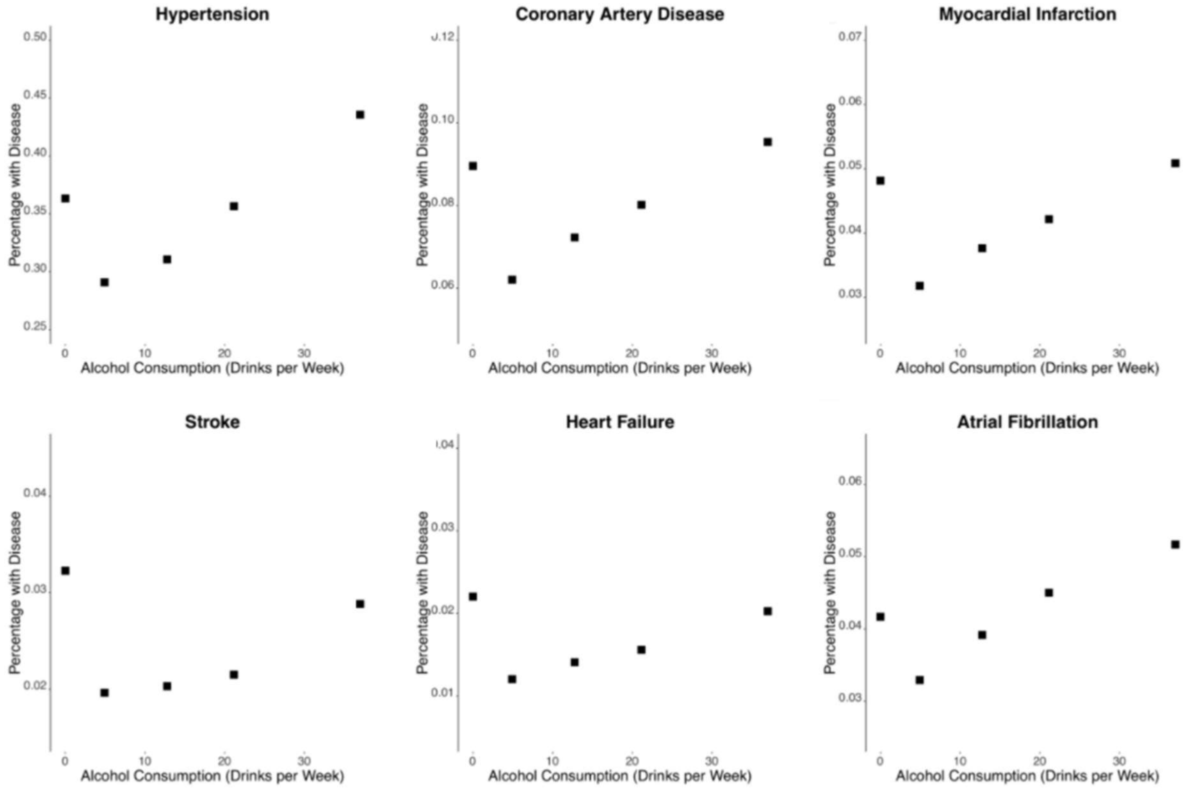

© 2022 Biddinger KJ et al. *JAMA Network Open.*

eFigure 2. Secondary Analyses for Confounding in Epidemiological Associations Between Alcohol Consumption and Cardiovascular Disease

(I) Secondary cardiovascular disease phenotypes: Myocardial infarction, stroke, heart failure, and atrial fibrillation. Baseline cox proportional hazards models are shown in black, and lifestyle-adjusted models models are shown in gray. Lifestyle factors were smoking, BMI, red meat intake, vegetable intake, physical activity, and self-reported health.

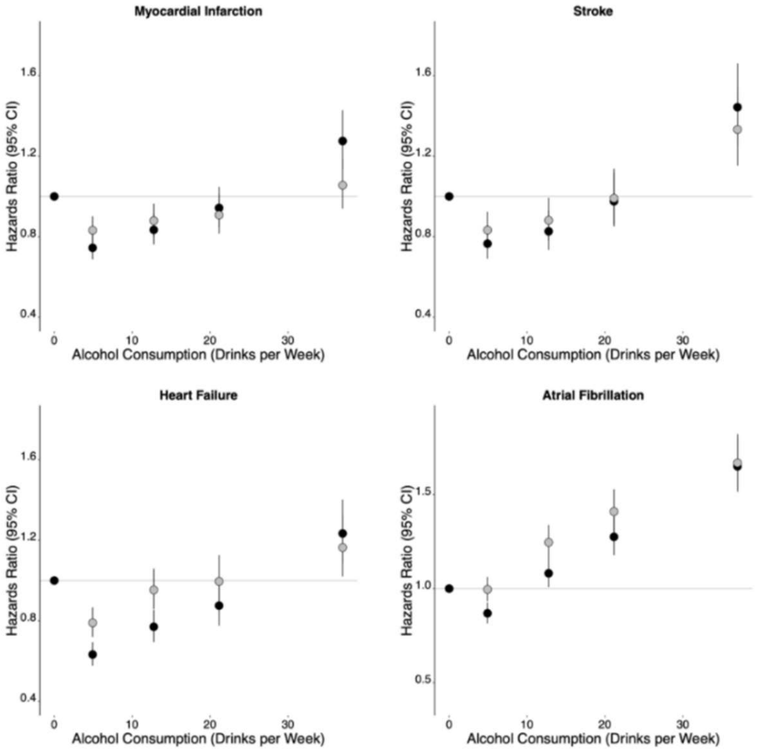

© 2022 Biddinger KJ et al. *JAMA Network Open.*

(II) Sensitivity analyses for hypertension: unadjusted for self-reported health, men only, and women only. Baseline cox proportional hazards models are shown in black, and lifestyle-adjusted models models are shown in gray. Unless otherwise noted, lifestyle factors were smoking, BMI, red meat intake, vegetable intake, physical activity, and self-reported health.

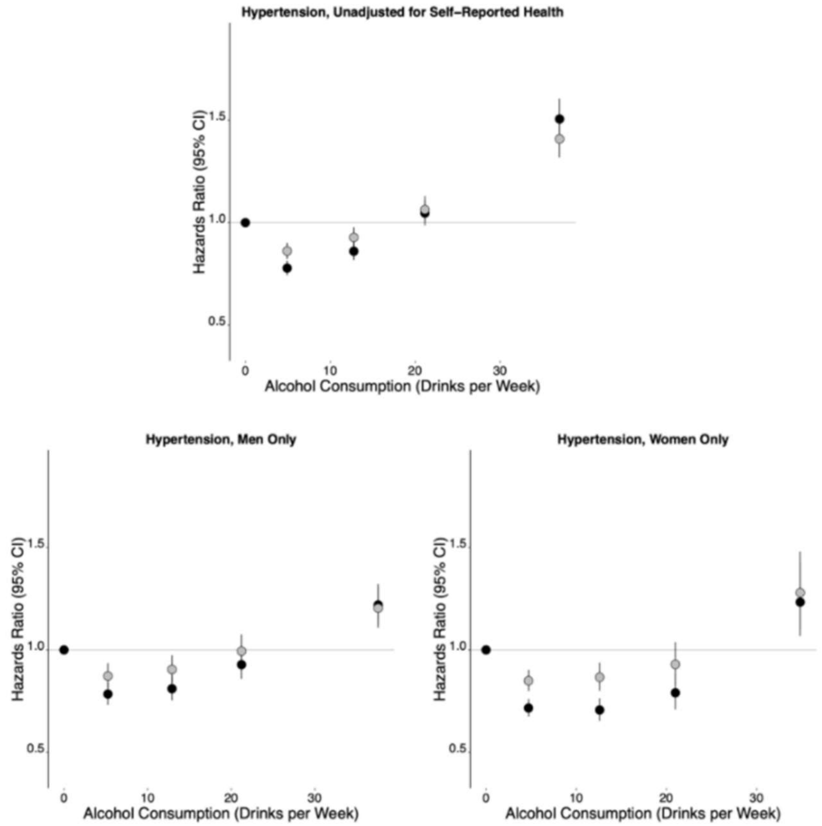

© 2022 Biddinger KJ et al. *JAMA Network Open.*

eFigure 3. Mean Values of 6 Different Lifestyle Factors Within Alcohol Consumption Subcategories

Self-reported health is coded such that a lower value represents a better description of health.

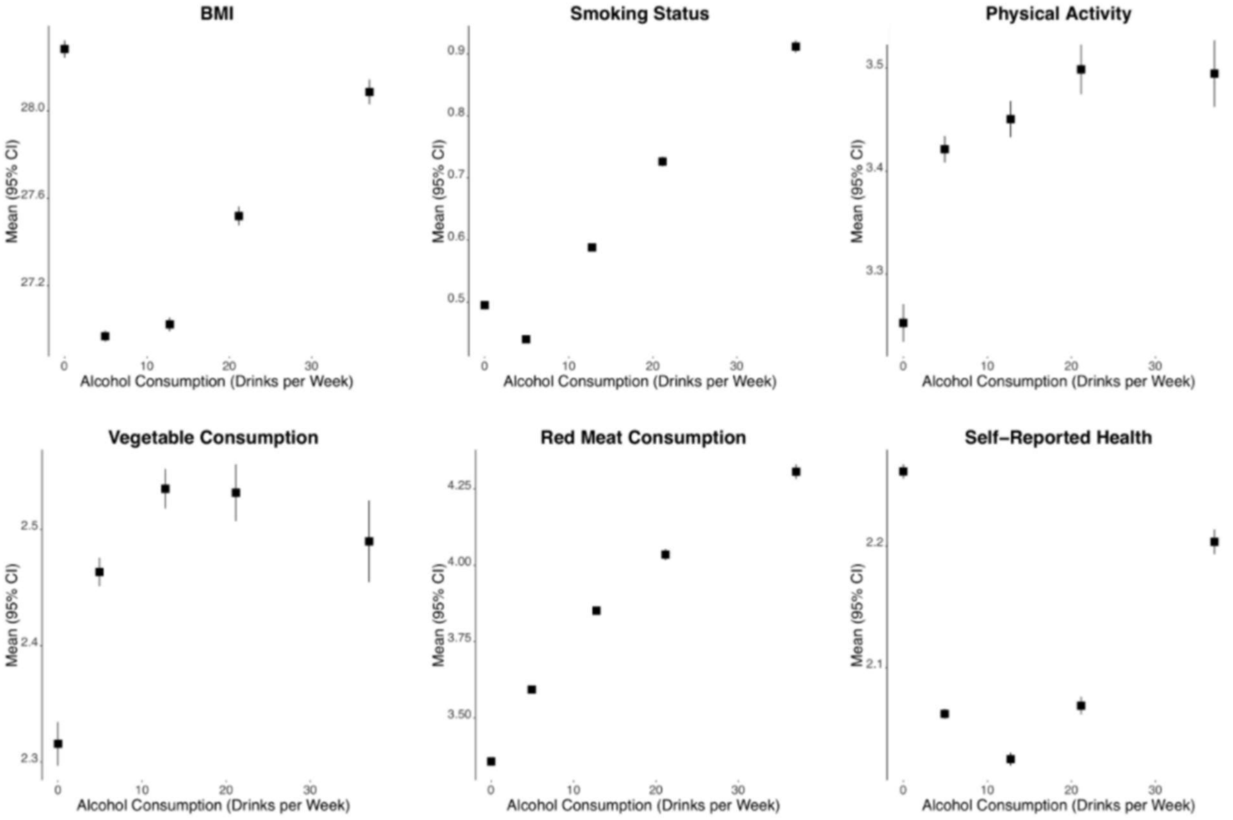

© 2022 Biddinger KJ et al. *JAMA Network Open.*

eFigure 4. Genetic Associations of Alcohol With CVD Phenotypes Using Allele Scores

(I) Allele score associations using AUD-R genetic instrument. Models were run in (A) all subjects, (B) current drinkers, and (C) lifelong abstainers. Associations were determined using logistic regression models adjusting for age at assessment, sex, genotyping array, and principle components 1-10.

A

Disease Phenotype	Incident Cases, No.	Controls, No.	Odds Ratio (95%CI)	P value
Coronary artery disease	27667	343796	1.570 [1.294; 1.906]	4.86e-06
Myocardial infarction	14503	356960	1.540 [1.188; 1.997]	0.001
Heart failure	5812	365651	1.413 [0.948; 2.108]	0.090
Stroke	8710	362753	1.266 [0.915; 1.752]	0.155
Atrial fibrillation	14367	357096	1.422 [1.099; 1.839]	0.007
Hypertension	121708	249755	1.506 [1.354; 1.677]	6.07e-14

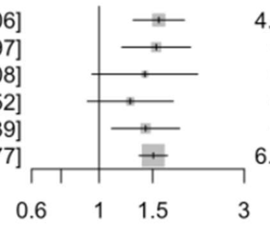

B

Disease Phenotype	Incident Cases, No.	Controls, No.	Odds Ratio (95%CI)	P value
Coronary artery disease	20371	269543	1.929 [1.526; 2.438]	3.86e-08
Myocardial infarction	10586	279328	1.887 [1.376; 2.587]	8.15e-05
Heart failure	4018	285701	1.945 [1.173; 3.226]	0.010
Stroke	6078	283836	1.602 [1.065; 2.408]	0.024
Atrial fibrillation	11012	278902	1.553 [1.146; 2.106]	0.005
Hypertension	92179	197735	1.660 [1.462; 1.884]	4.41e-15

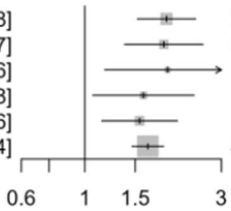

C

Disease Phenotype	Incident Cases, No.	Controls, No.	Odds Ratio (95%CI)	P value
Coronary artery disease	2540	31962	1.506 [0.857; 2.648]	0.155
Myocardial infarction	1311	33191	1.701 [0.785; 3.685]	0.178
Heart failure	603	33881	0.439 [0.161; 1.196]	0.107
Stroke	932	33570	1.494 [0.610; 3.658]	0.379
Atrial fibrillation	1241	33261	0.897 [0.424; 1.898]	0.776
Hypertension	11784	22718	1.359 [1.003; 1.842]	0.048

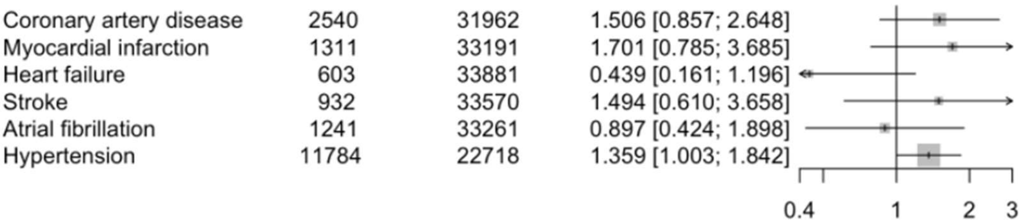

© 2022 Biddinger KJ et al. *JAMA Network Open.*

(II) Allele score associations using AUDIT-C-R genetic instrument. Models were run in (A) all subjects, (B) current drinkers, and (C) lifelong abstainers. Associations were determined using logistic regression models adjusting for age at assessment, sex, genotyping array, and principle components 1-10.

A

Disease Phenotype	Incident Cases, No.	Controls, No.	Odds Ratio (95%CI)	P value
Coronary artery disease	27667	343796	1.338 [1.129; 1.585]	7.56e-04
Myocardial infarction	14503	356960	1.217 [0.971; 1.526]	0.088
Heart failure	5812	365651	1.151 [0.813; 1.630]	0.428
Stroke	8710	362753	1.112 [0.837; 1.479]	0.463
Atrial fibrillation	14367	357096	1.238 [0.988; 1.552]	0.063
Hypertension	121708	249755	1.180 [1.074; 1.297]	5.71e-04

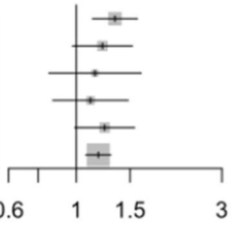

B

Disease Phenotype	Incident Cases, No.	Controls, No.	Odds Ratio (95%CI)	P value
Coronary artery disease	20371	269543	1.641 [1.338; 2.012]	1.93e-06
Myocardial infarction	10586	279328	1.475 [1.122; 1.938]	0.005
Heart failure	4018	285701	1.359 [0.882; 2.096]	0.164
Stroke	6078	283836	1.357 [0.953; 1.934]	0.091
Atrial fibrillation	11012	278902	1.329 [1.019; 1.732]	0.036
Hypertension	92179	197735	1.333 [1.193; 1.489]	3.85e-07

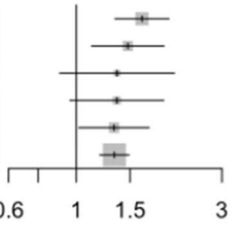

C

Disease Phenotype	Incident Cases, No.	Controls, No.	Odds Ratio (95%CI)	P value
Coronary artery disease	2540	31962	1.302 [0.786; 2.158]	0.305
Myocardial infarction	1311	33191	1.489 [0.746; 2.971]	0.259
Heart failure	603	33881	0.823 [0.313; 2.159]	0.692
Stroke	932	33570	1.203 [0.544; 2.661]	0.648
Atrial fibrillation	1241	33261	0.790 [0.402; 1.550]	0.493
Hypertension	11784	22718	1.066 [0.810; 1.401]	0.649

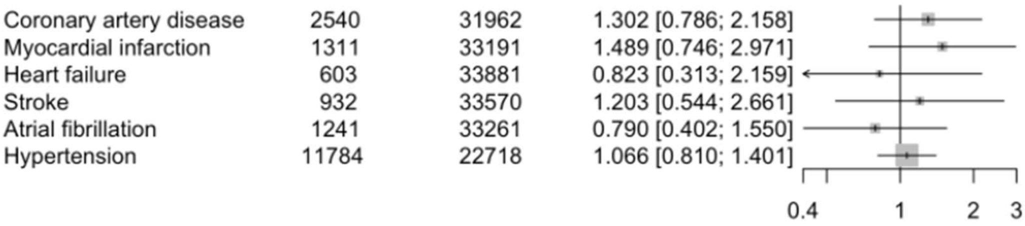

© 2022 Biddinger KJ et al. *JAMA Network Open.*

(III) Allele score associations using AUD genetic instrument. Models were run in (A) all subjects, (B) current drinkers, and (C) lifelong abstainers. Associations were determined using logistic regression models adjusting for age at assessment, sex, genotyping array, and principle components 1-10.

A

Disease Phenotype	Incident Cases, No.	Controls, No.	Odds Ratio (95%CI)	P value
Coronary artery disease	27667	343796	1.344 [1.150; 1.570]	1.97e-04
Myocardial infarction	14503	356960	1.274 [1.035; 1.568]	0.022
Heart failure	5812	365651	1.101 [0.801; 1.513]	0.555
Stroke	8710	362753	1.148 [0.884; 1.492]	0.301
Atrial fibrillation	14367	357096	1.224 [0.995; 1.506]	0.056
Hypertension	121708	249755	1.328 [1.217; 1.448]	1.55e-10

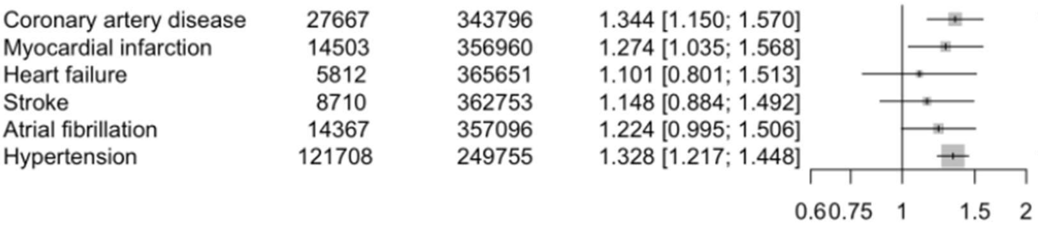

B

Disease Phenotype	Incident Cases, No.	Controls, No.	Odds Ratio (95%CI)	P value
Coronary artery disease	20371	269543	1.588 [1.317; 1.913]	1.19e-06
Myocardial infarction	10586	279328	1.510 [1.175; 1.940]	0.001
Heart failure	4018	285701	1.584 [1.061; 2.365]	0.024
Stroke	6078	283836	1.417 [1.023; 1.962]	0.036
Atrial fibrillation	11012	278902	1.367 [1.071; 1.744]	0.012
Hypertension	92179	197735	1.502 [1.356; 1.663]	5.88e-15

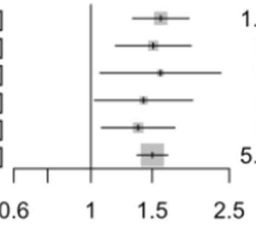

C

Disease Phenotype	Incident Cases, No.	Controls, No.	Odds Ratio (95%CI)	P value
Coronary artery disease	2540	31962	1.427 [0.893; 2.282]	0.137
Myocardial infarction	1311	33191	1.263 [0.672; 2.374]	0.469
Heart failure	603	33881	0.381 [0.164; 0.883]	0.024
Stroke	932	33570	1.469 [0.697; 3.096]	0.312
Atrial fibrillation	1241	33261	0.865 [0.461; 1.622]	0.651
Hypertension	11784	22718	1.095 [0.850; 1.412]	0.482

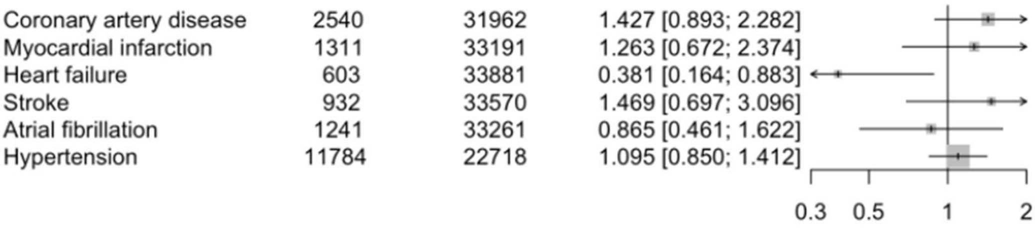

© 2022 Biddinger KJ et al. *JAMA Network Open.*

(IV) Allele score associations using AUDIT-C genetic instrument. Models were run in (A) all subjects, (B) current drinkers, and (C) lifelong abstainers. Associations were determined using logistic regression models adjusting for age at assessment, sex, genotyping array, and principle components 1-10.

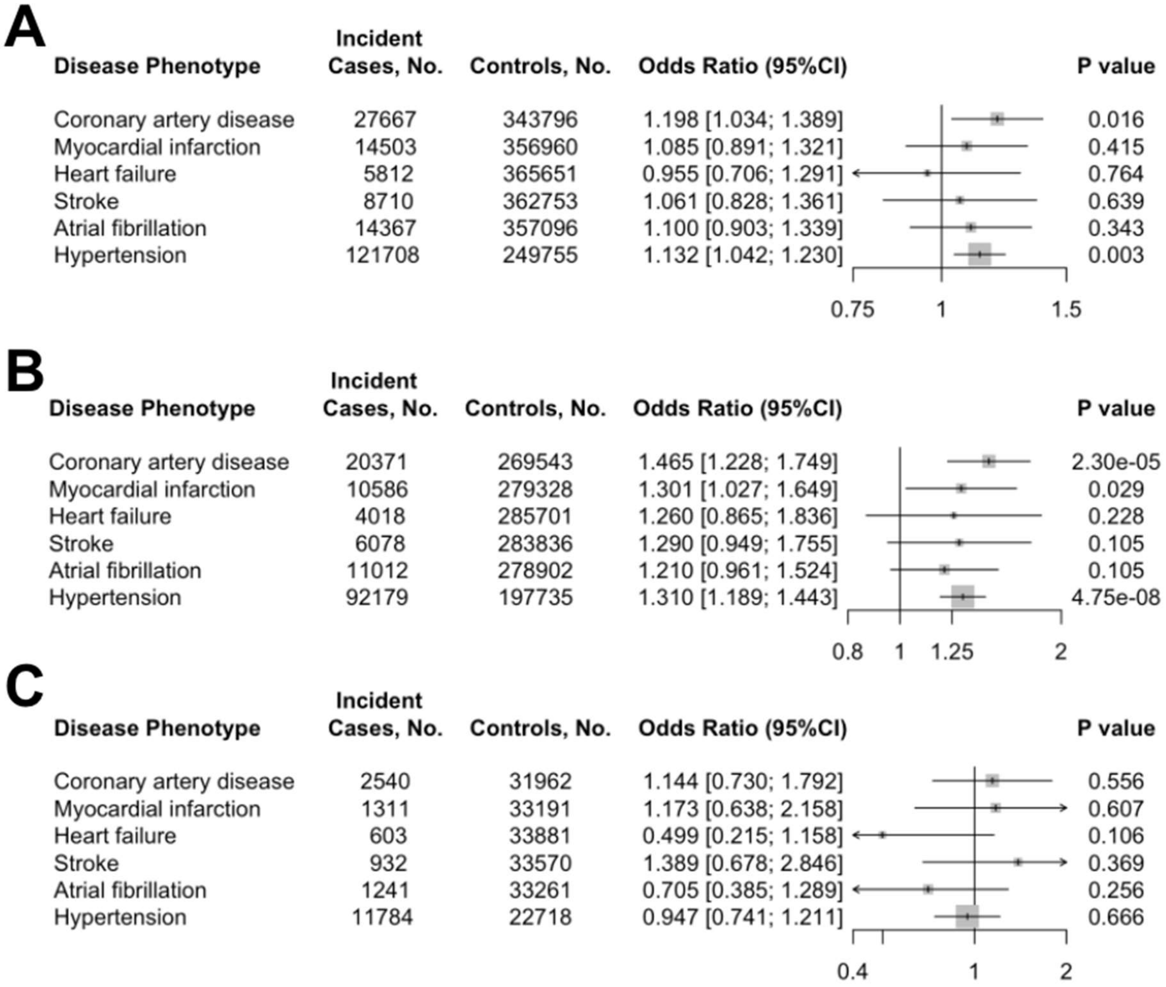

© 2022 Biddinger KJ et al. *JAMA Network Open.*

eFigure 5. Genetic Allele Score Associations of Alcohol With CVD Phenotypes Stratified by Category of Alcohol Consumption

(I) Genetic associations between AUD-R genetic risk score and heart disease phenotypes in (A) light drinkers, (B) moderate drinkers, (C) heavy drinkers, and (D) abusive drinkers. Associations were determined using logistic regression models adjusting for age at assessment, sex, genotyping array, and principle components 1-10.

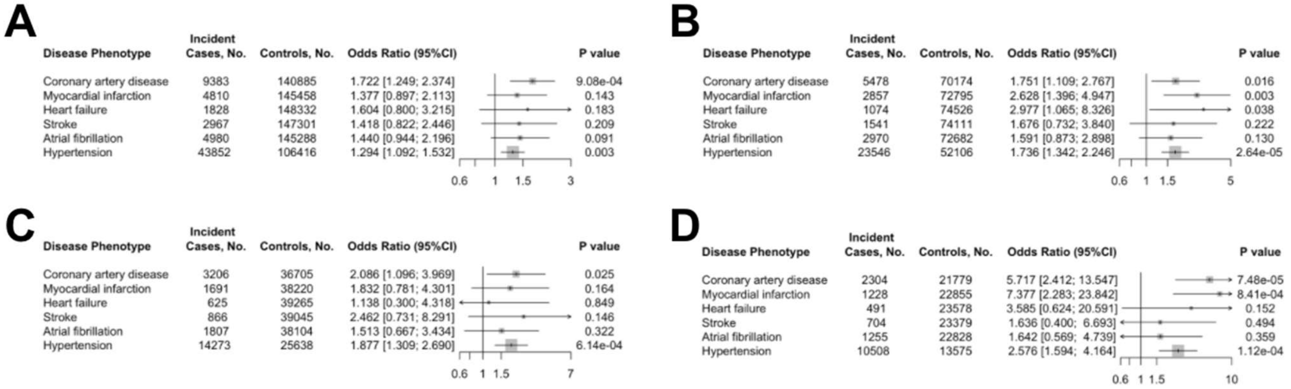

(II) Genetic associations between AUDIT-C-R genetic risk score and heart disease phenotypes in (A) light drinkers, (B) moderate drinkers, (C) heavy drinkers, and (D) abusive drinkers. Associations were determined using logistic regression models adjusting for age at assessment, sex, genotyping array, and principle components 1-10.

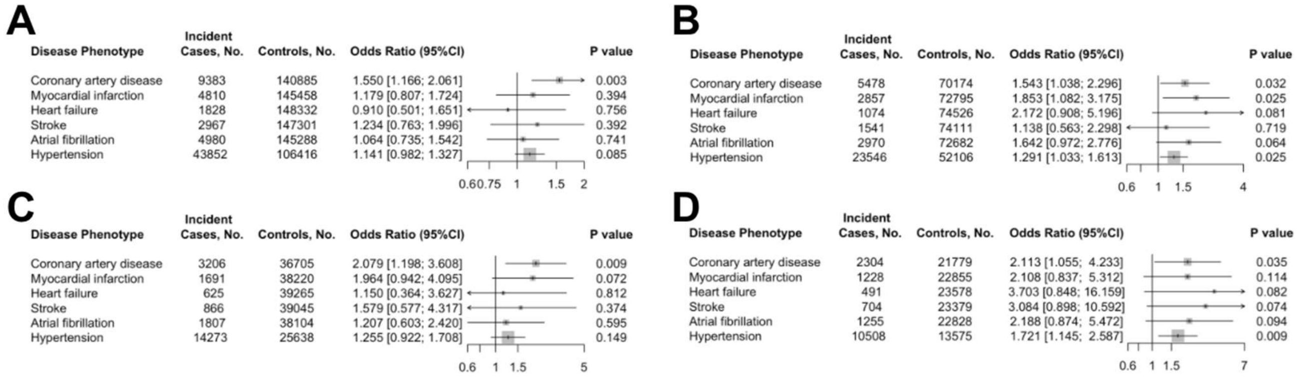

© 2022 Biddinger KJ et al. *JAMA Network Open.*

eFigure 6. Fractional Polynomial Nonlinear MR Analyses, Using AUD-R Genetic Instruments, of Alcohol and Secondary Cardiovascular Disease Phenotypes

LACE values were meta-regressed against mean consumption in each strata of alcohol, and these plots were reconstructed as the derivative of the best fit model. Shaded areas denote 95% confidence intervals for the model.

I) In all individuals

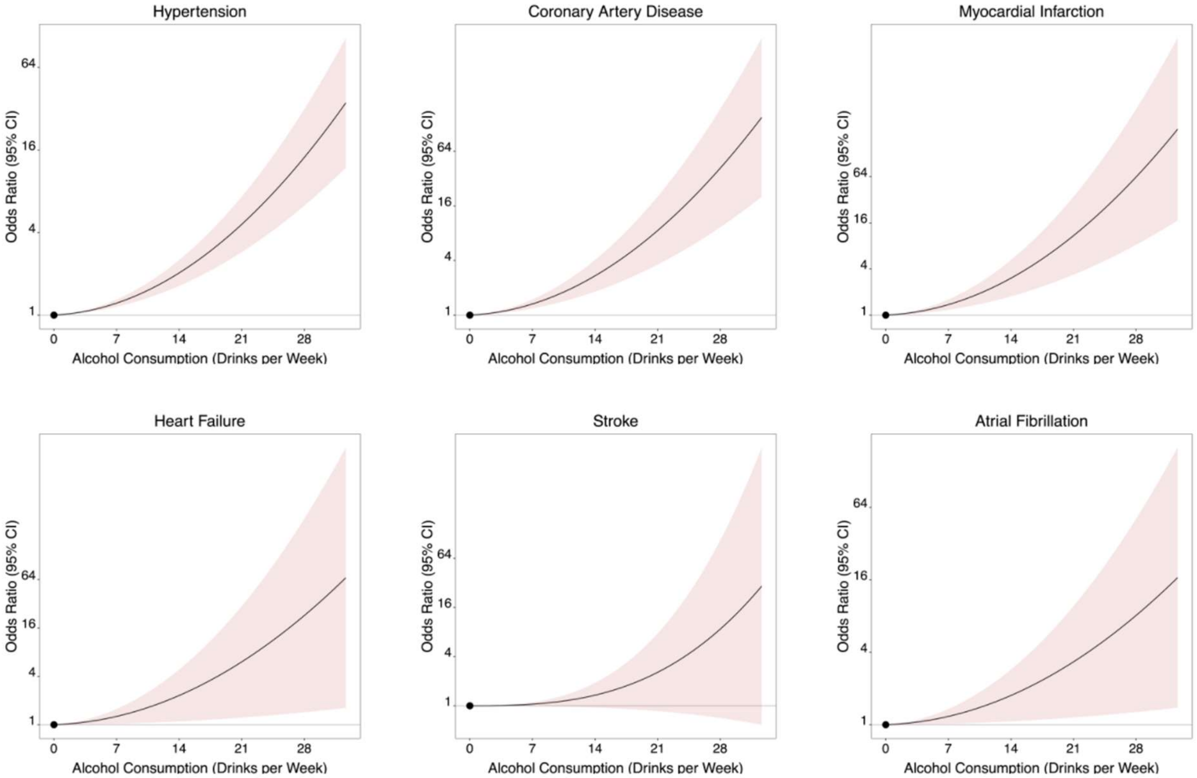

© 2022 Biddinger KJ et al. *JAMA Network Open.*

II) Excluding abstainers

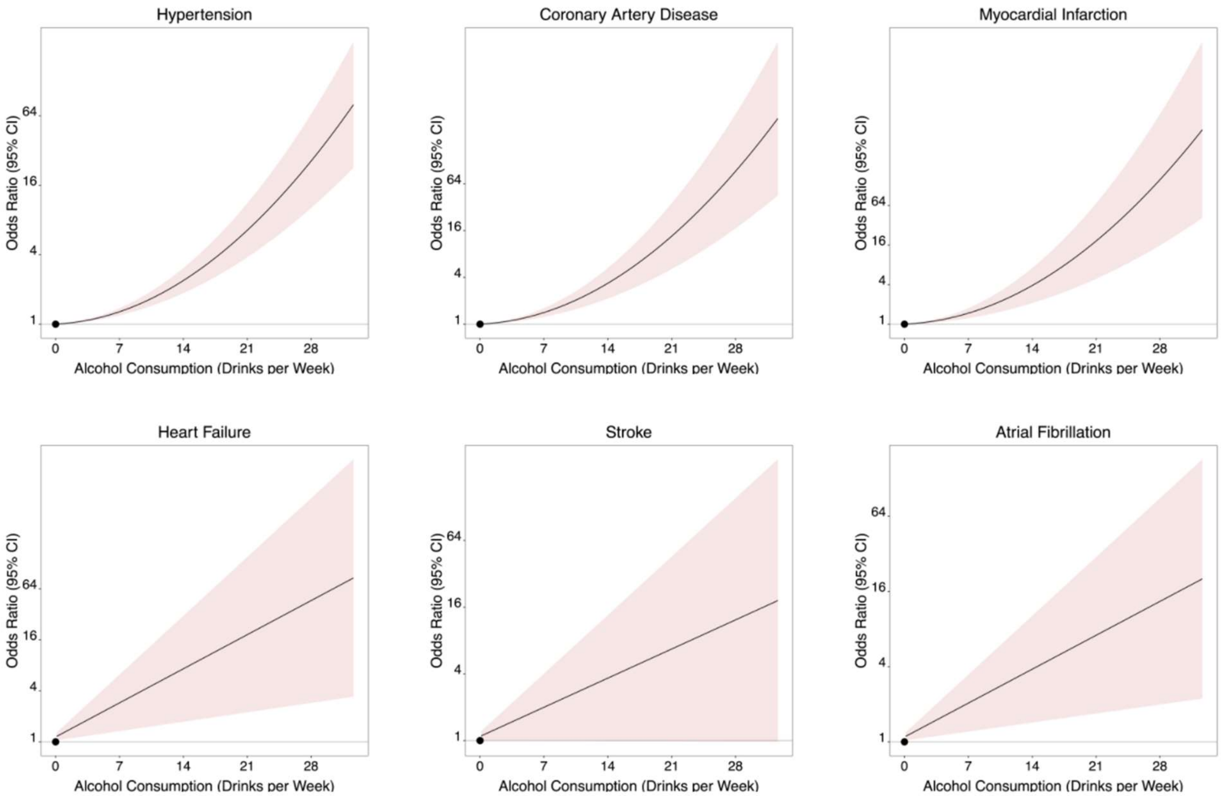

© 2022 Biddinger KJ et al. *JAMA Network Open.*

eFigure 7. Nonlinear MR Analyses of Alcohol and Total Mortality

Associations were tested using fractional polynomial (A) or piecewise linear methodology (B). LACE values were calculated using the AUD-R allele score. Shaded areas and error bars refer to the 95% confidence interval.

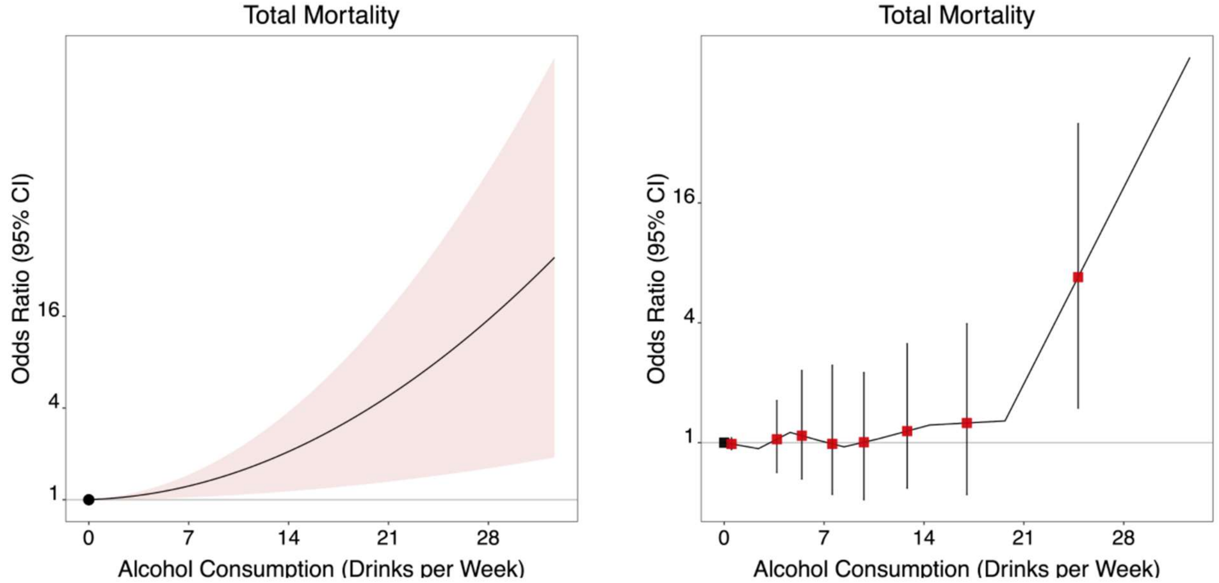

© 2022 Biddinger KJ et al. *JAMA Network Open.*

eFigure 8. Fractional Polynomial Nonlinear MR Analyses, Using Secondary Genetic Instruments, of Alcohol and 6 Cardiovascular Disease Phenotypes

LACE values were meta-regressed against mean consumption in each strata of alcohol intake, and these plots were reconstructed as the derivative of the best fit model. Shaded areas denote 95% confidence intervals for the model.

(I) Using AUDIT-C-R genetic instrument, all individuals

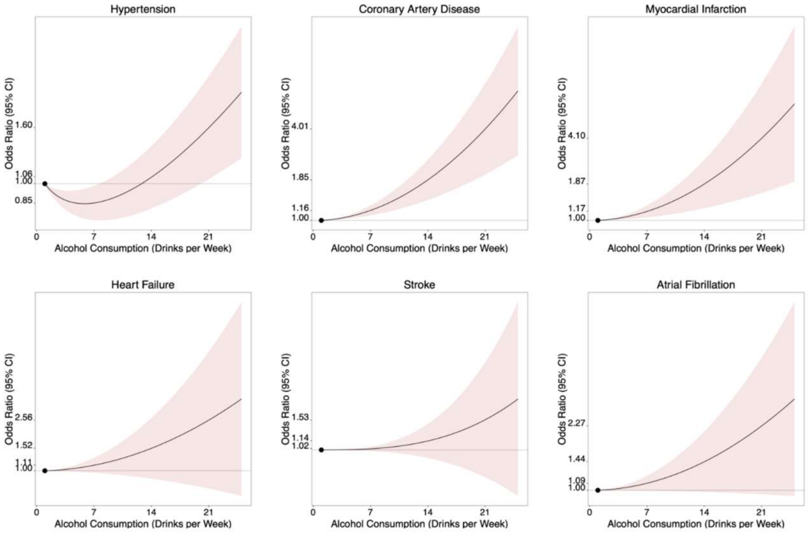

II) Using AUDIT-C-R instrument, excluding abstainers

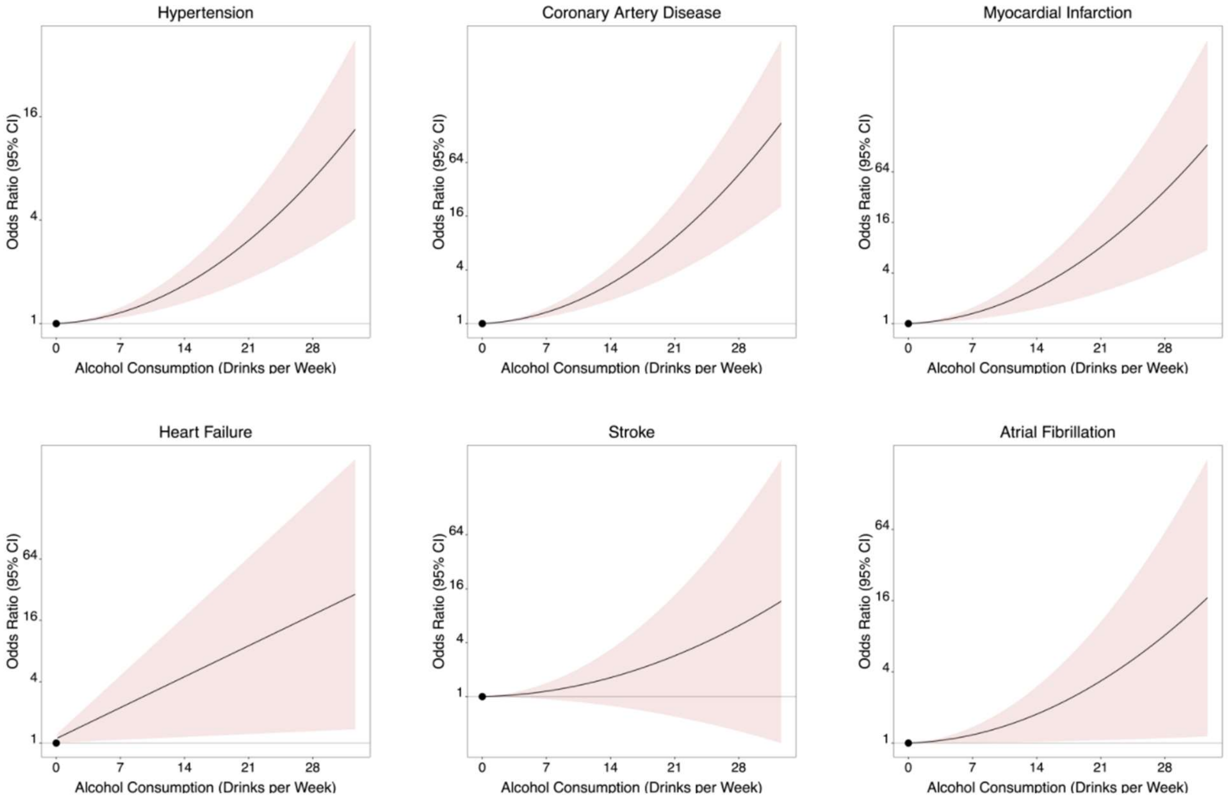

© 2022 Biddinger KJ et al. *JAMA Network Open.*

(III) Using number of alcohol-increasing alleles in rs1229984 in the *ADH1B* gene, all individuals

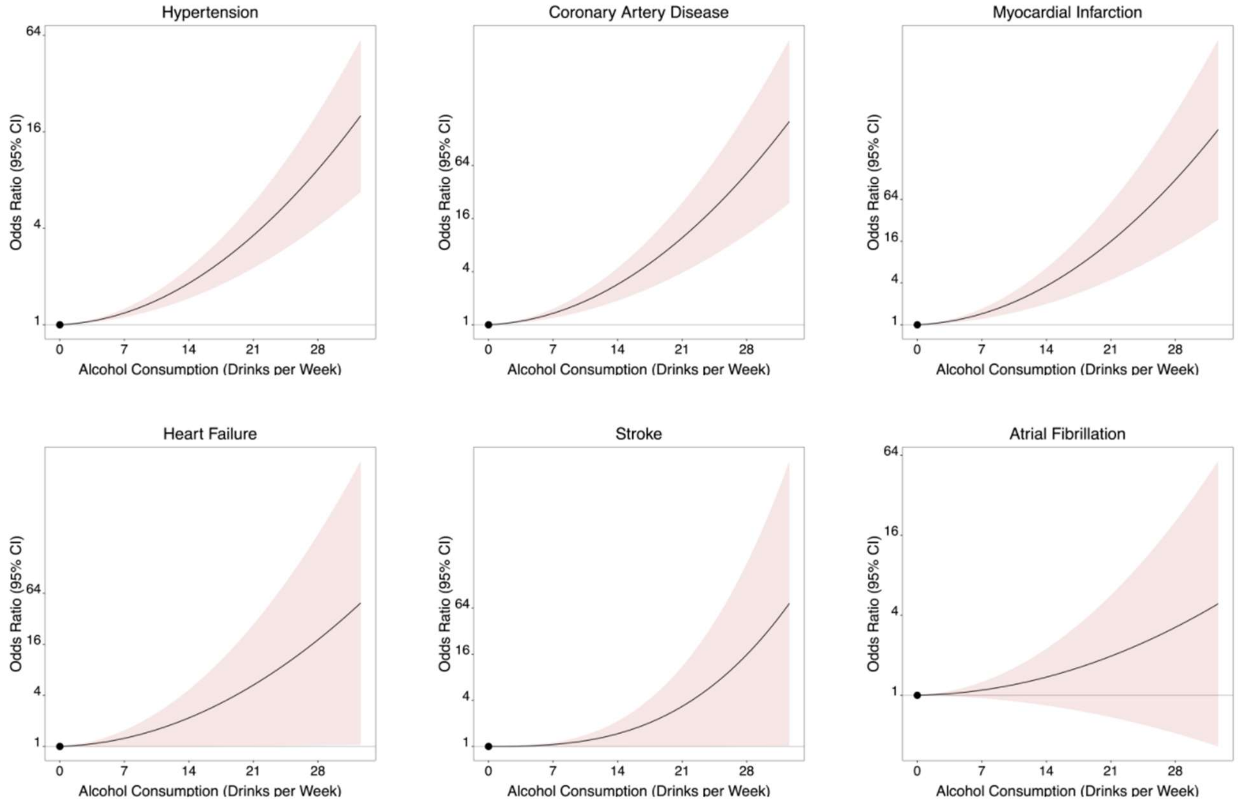

© 2022 Biddinger KJ et al. *JAMA Network Open.*

(IV) Using number of alcohol-increasing alleles in rs1229984 in the *ADH1B* gene, excluding abstainers

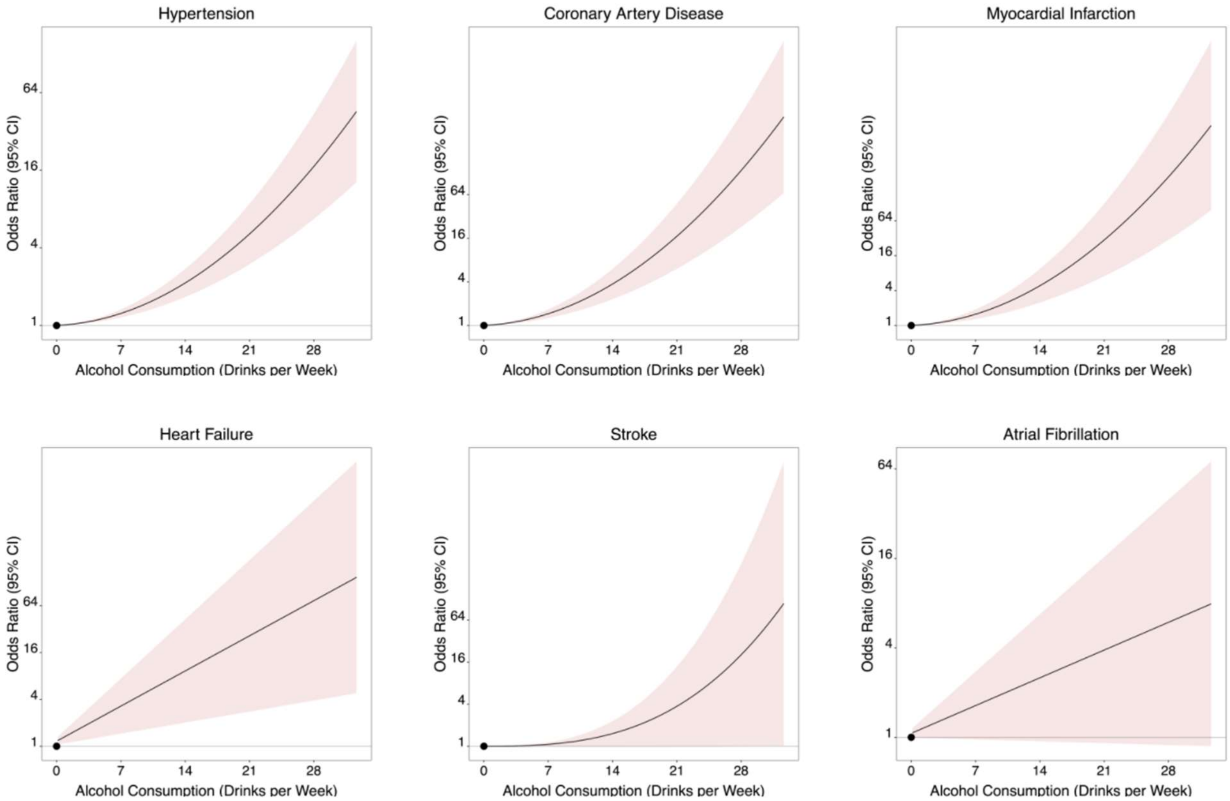

© 2022 Biddinger KJ et al. *JAMA Network Open.*

eFigure 9. Piecewise Nonlinear MR Analyses of Alcohol and 6 Cardiovascular Disease Phenotypes

Gradient at each value of weekly alcohol consumption is the localized average causal effect for the corresponding category of residual alcohol. Error bars refer to the 95% confidence interval.

(I) AUD-R genetic instrument, all individuals

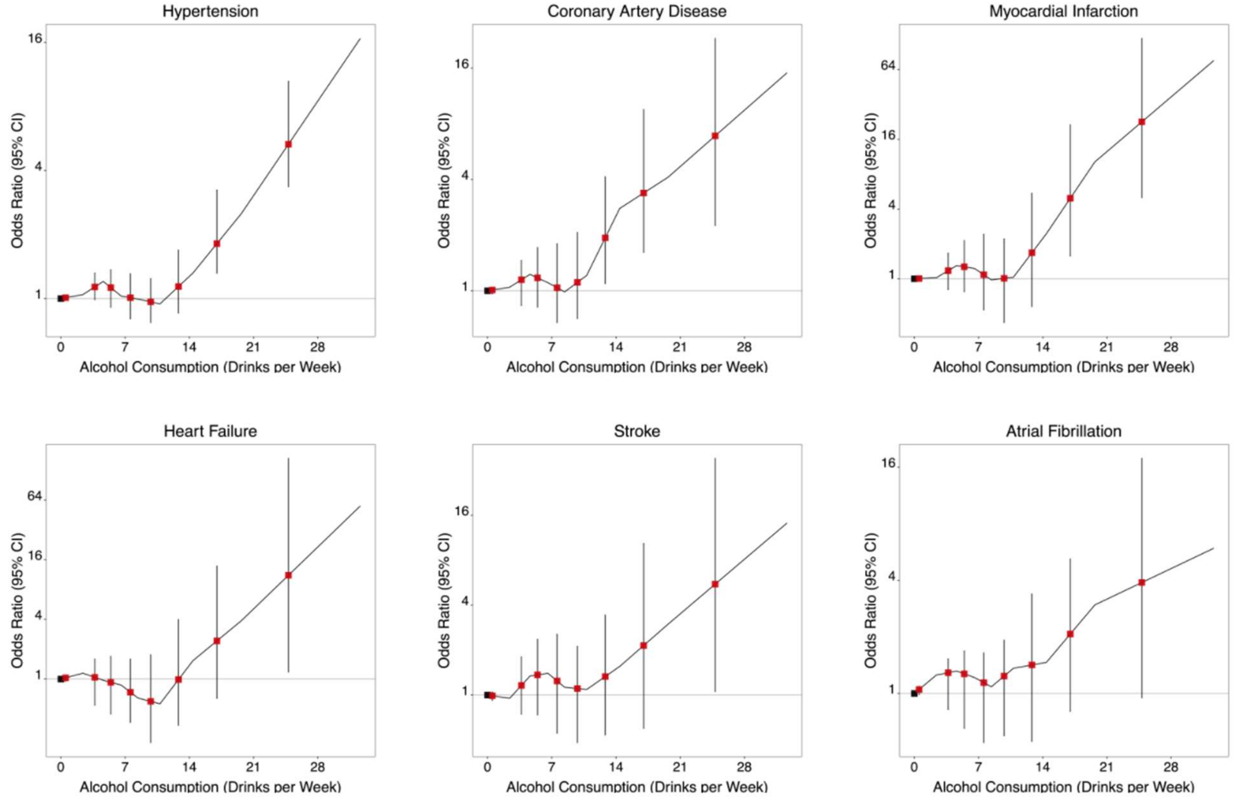

© 2022 Biddinger KJ et al. *JAMA Network Open.*

(II) AUD-R genetic instrument, excluding abstainers

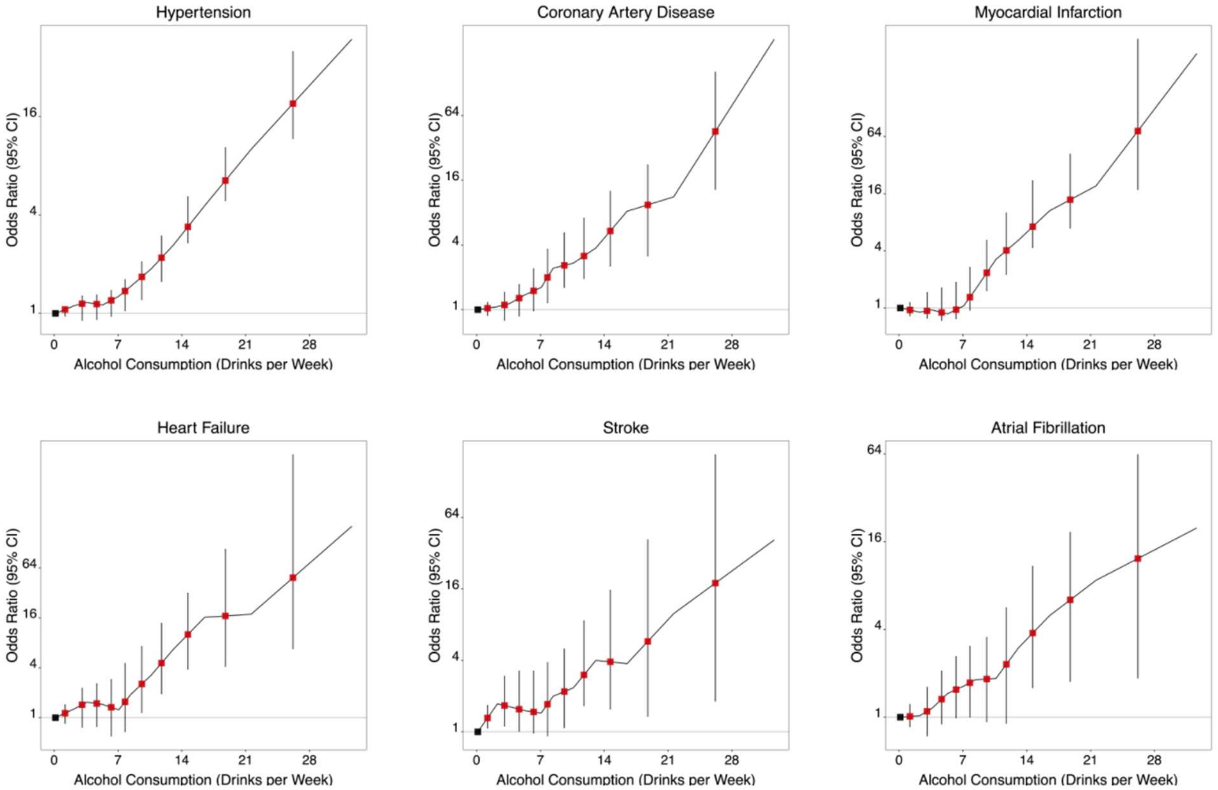

(III) AUDIT-C-R genetic instrument, all individuals

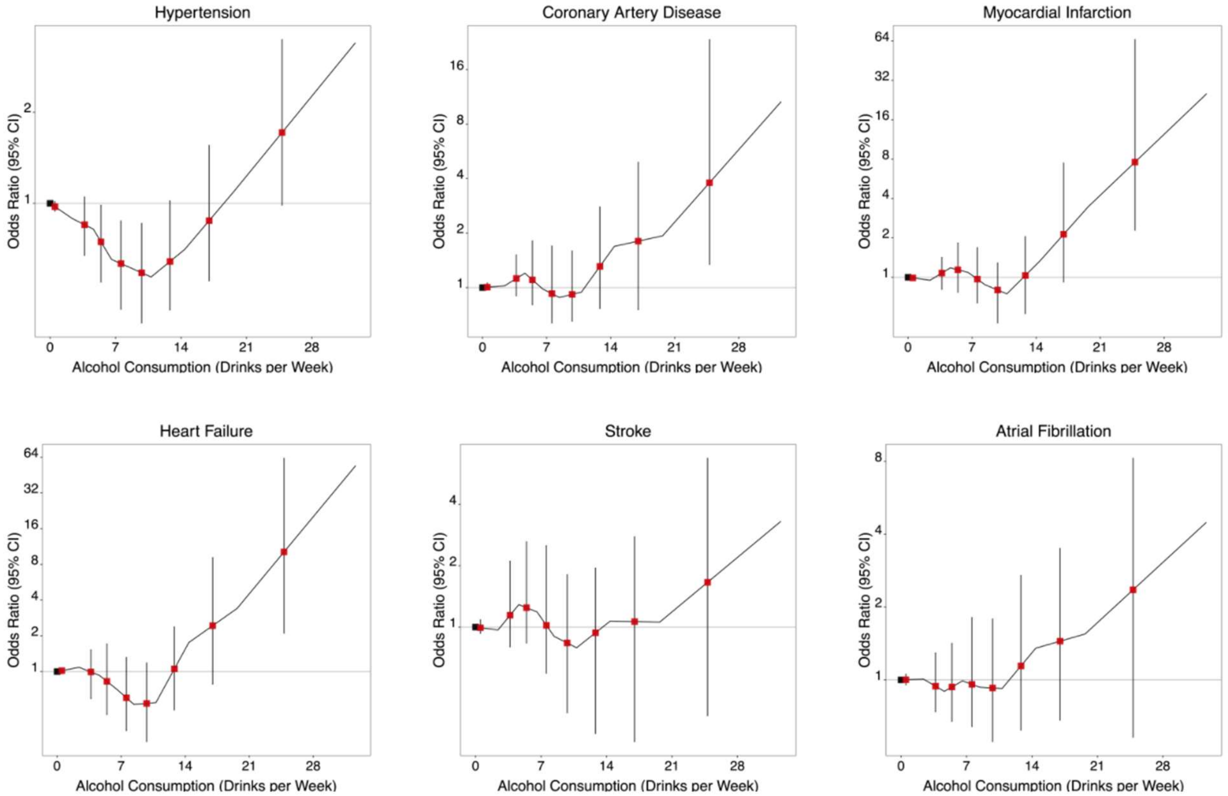

© 2022 Biddinger KJ et al. *JAMA Network Open.*

(IV) AUDIT-C-R genetic instrument, excluding abstainers

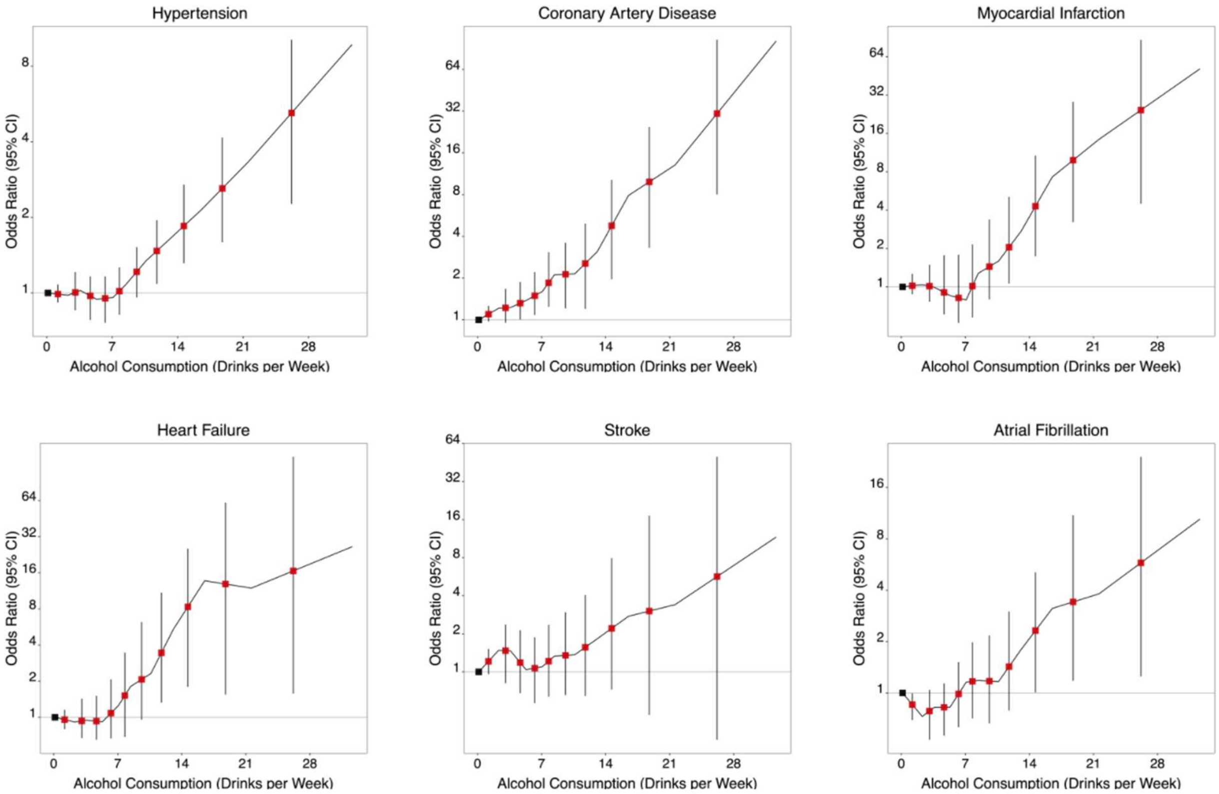

© 2022 Biddinger KJ et al. *JAMA Network Open.*

eFigure 10. Fractional Polynomial Nonlinear MR Analyses of Alcohol and Continuous Traits

(I) Fractional polynomial non-linear MR analyses for four primary continuous traits using AUD-R genetic instruments, in all individuals. Shaded areas denote 95% confidence intervals for the model.

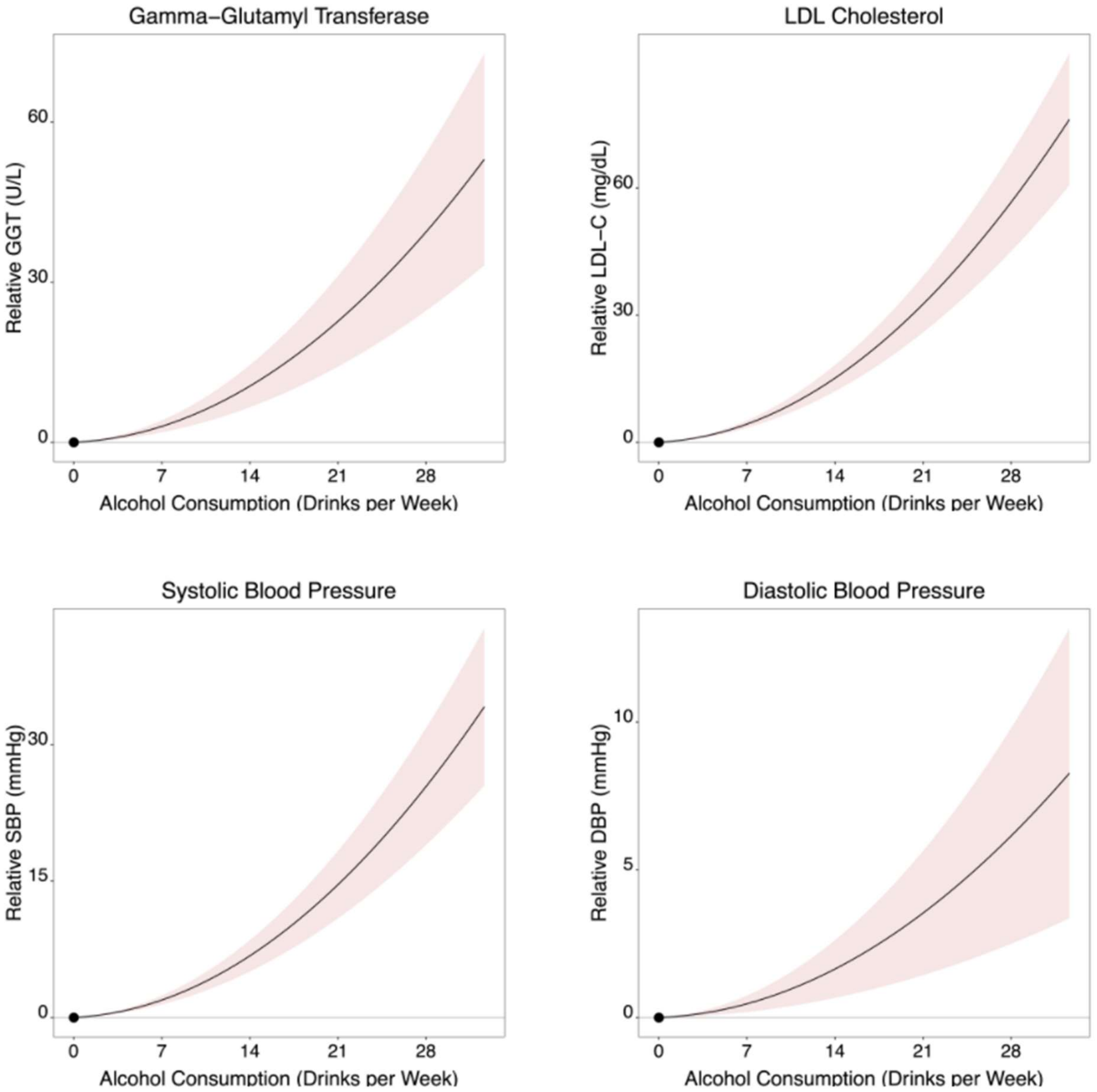

(II) Fractional polynomial non-linear MR analyses for six secondary continuous traits using AUD-R genetic instruments, in all individuals. Shaded areas denote 95% confidence intervals for the model.

© 2022 Biddinger KJ et al. *JAMA Network Open.*

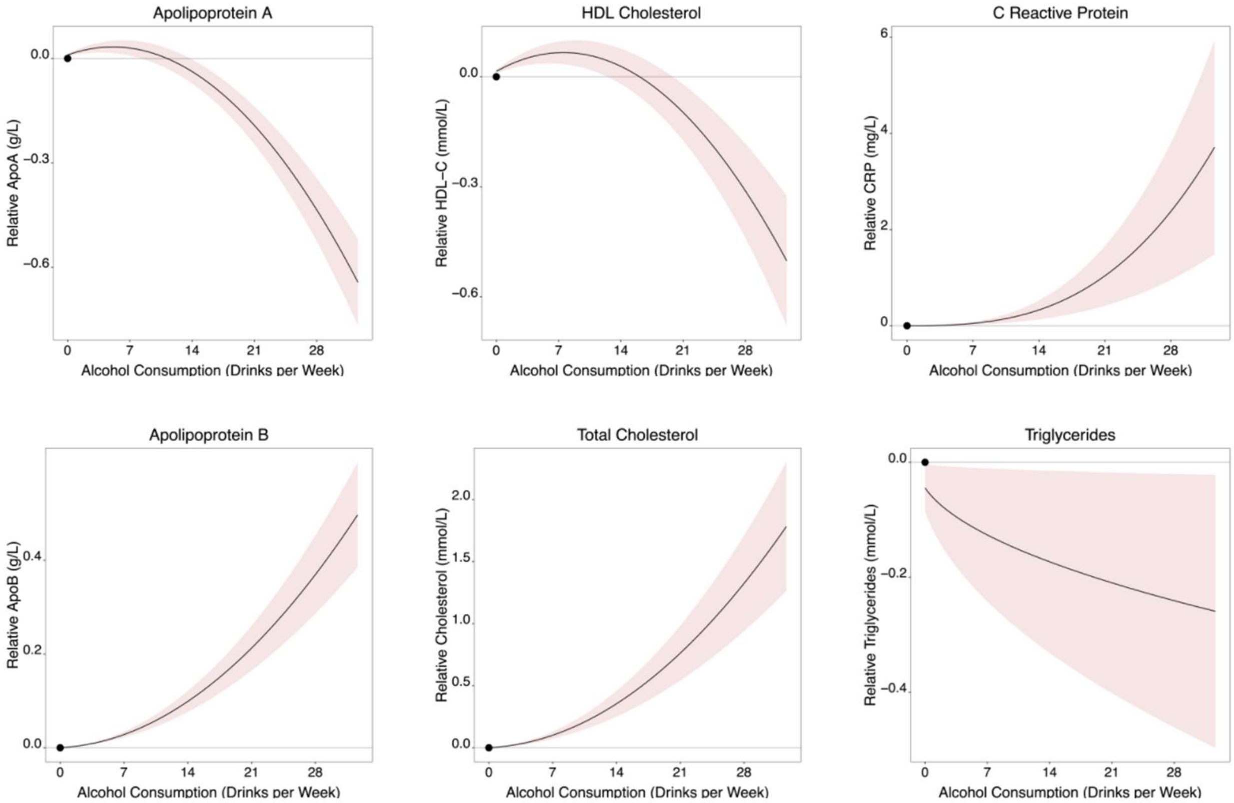

(III) Fractional polynomial non-linear MR analyses for four secondary continuous traits using AUD-R genetic instruments, excluding abstainers.

© 2022 Biddinger KJ et al. *JAMA Network Open.*

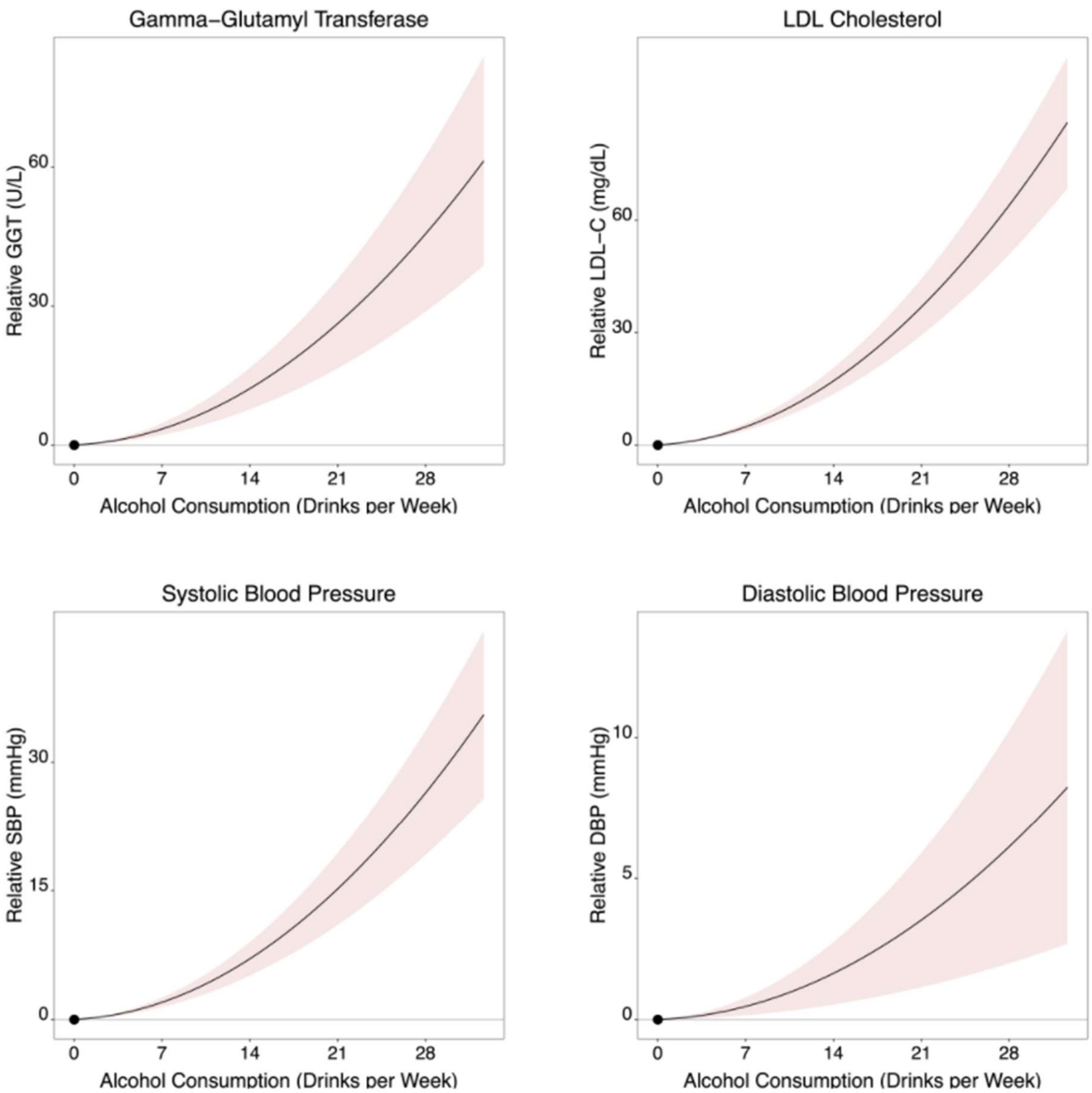

(IV) Fractional polynomial non-linear MR analyses for six secondary continuous traits using AUD-R genetic instruments, excluding abstainers.

© 2022 Biddinger KJ et al. *JAMA Network Open.*

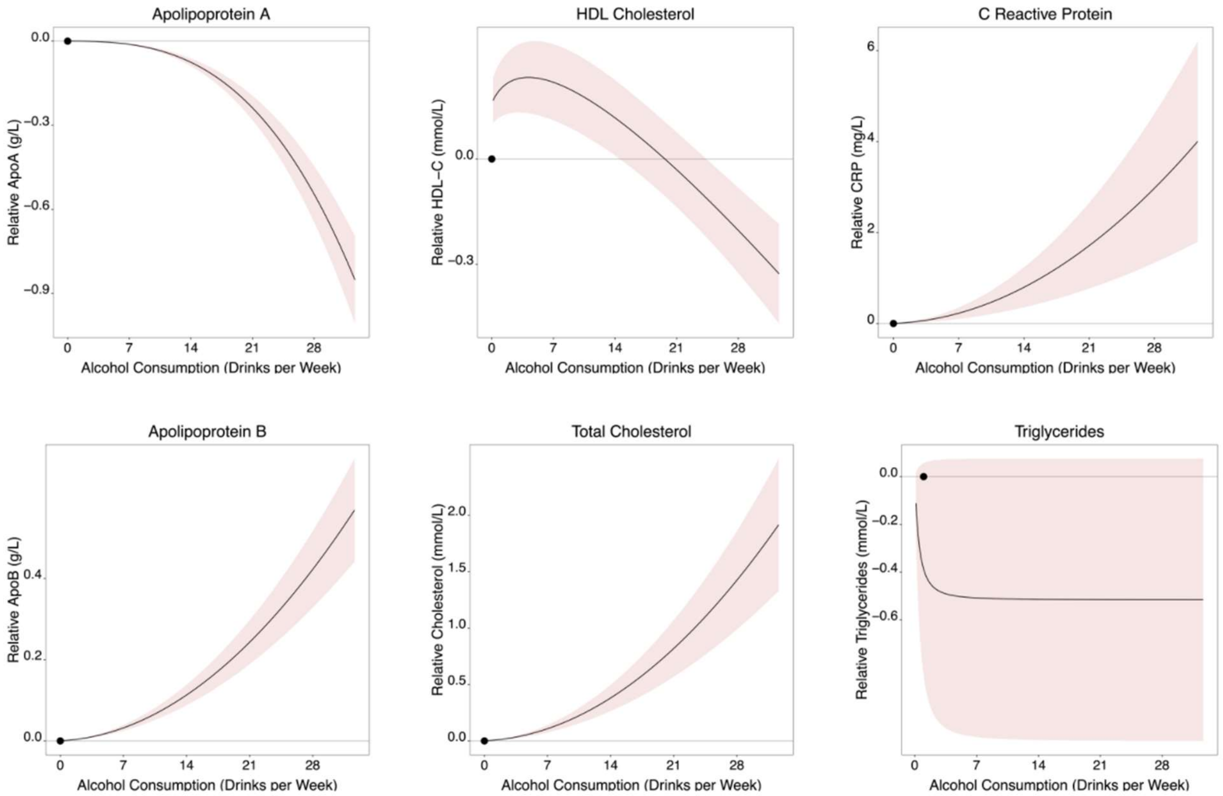

(V) Fractional polynomial non-linear MR analyses for four continuous traits using AUDIT-C-R genetic instruments. LACE values were meta-regressed against mean consumption in each strata of alcohol, and these plots were reconstructed as the derivative of the best fit model. Shaded areas denote 95% confidence intervals for the model.

© 2022 Biddinger KJ et al. *JAMA Network Open.*

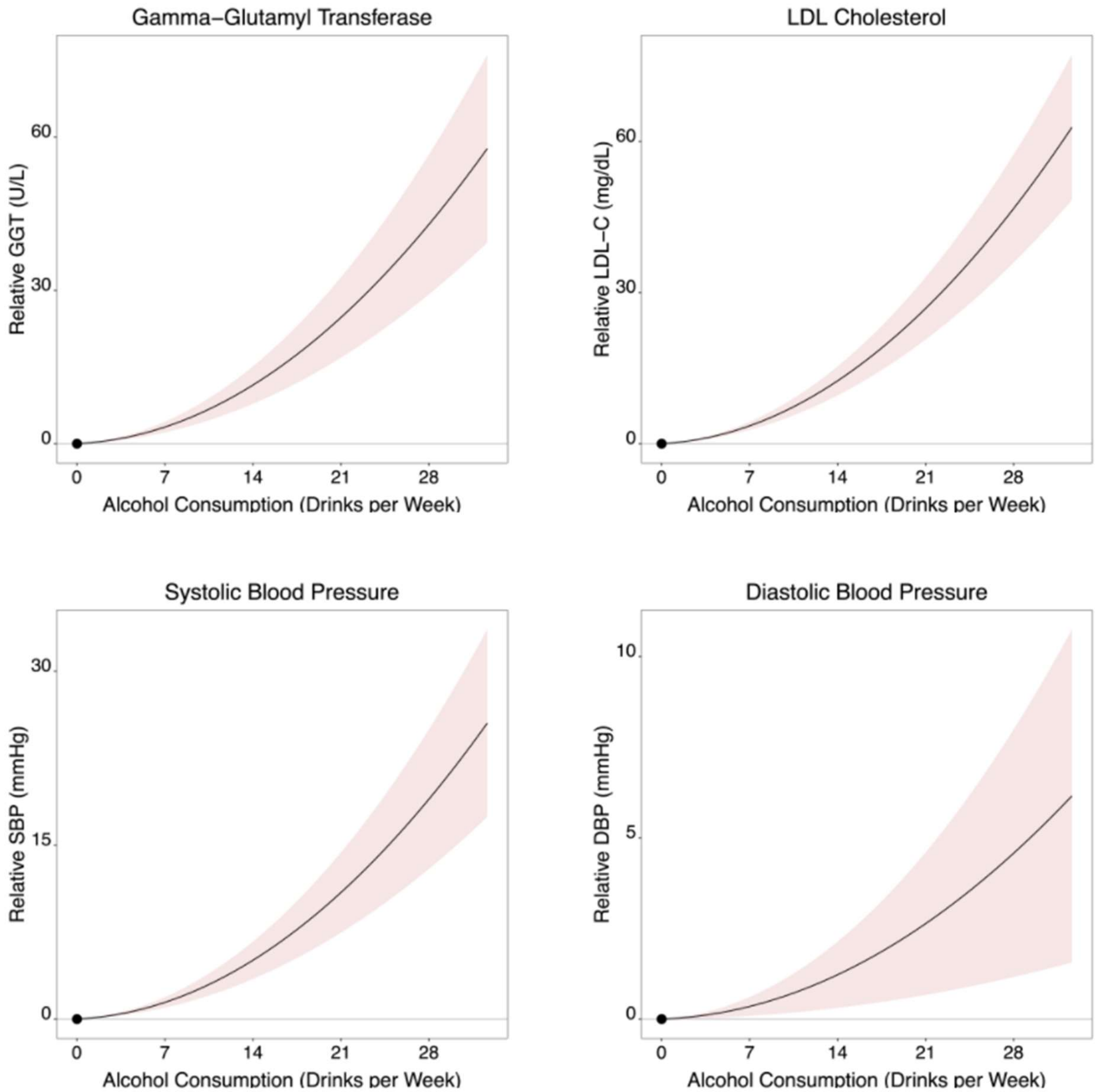

(VI) Fractional polynomial non-linear MR analyses for six secondary continuous traits using AUDIT-C-R genetic instruments. Shaded areas denote 95% confidence intervals for the model.

© 2022 Biddinger KJ et al. *JAMA Network Open.*

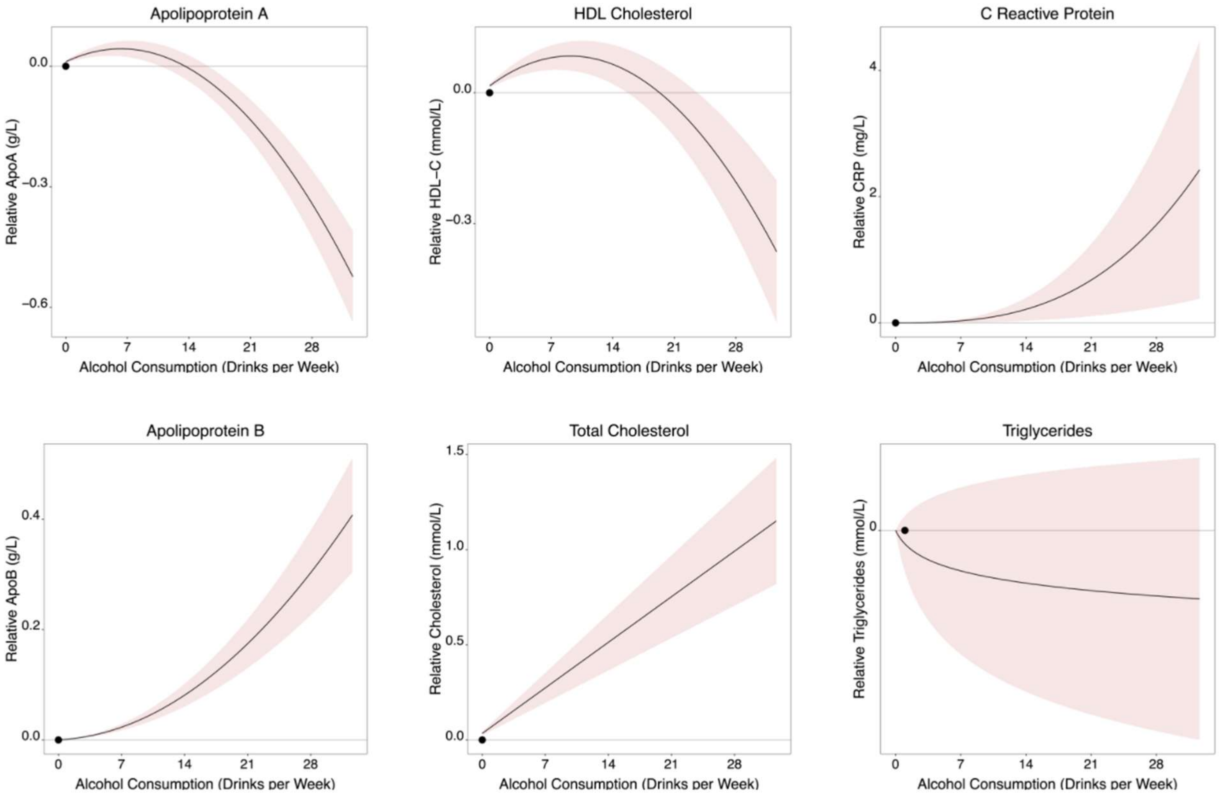

(VII) Fractional polynomial non-linear MR analyses for four continuous traits using rs1229984 from the biologically relevant *ADH1B* genetic instruments. Shaded areas denote 95% confidence intervals for the model.

© 2022 Biddinger KJ et al. *JAMA Network Open.*

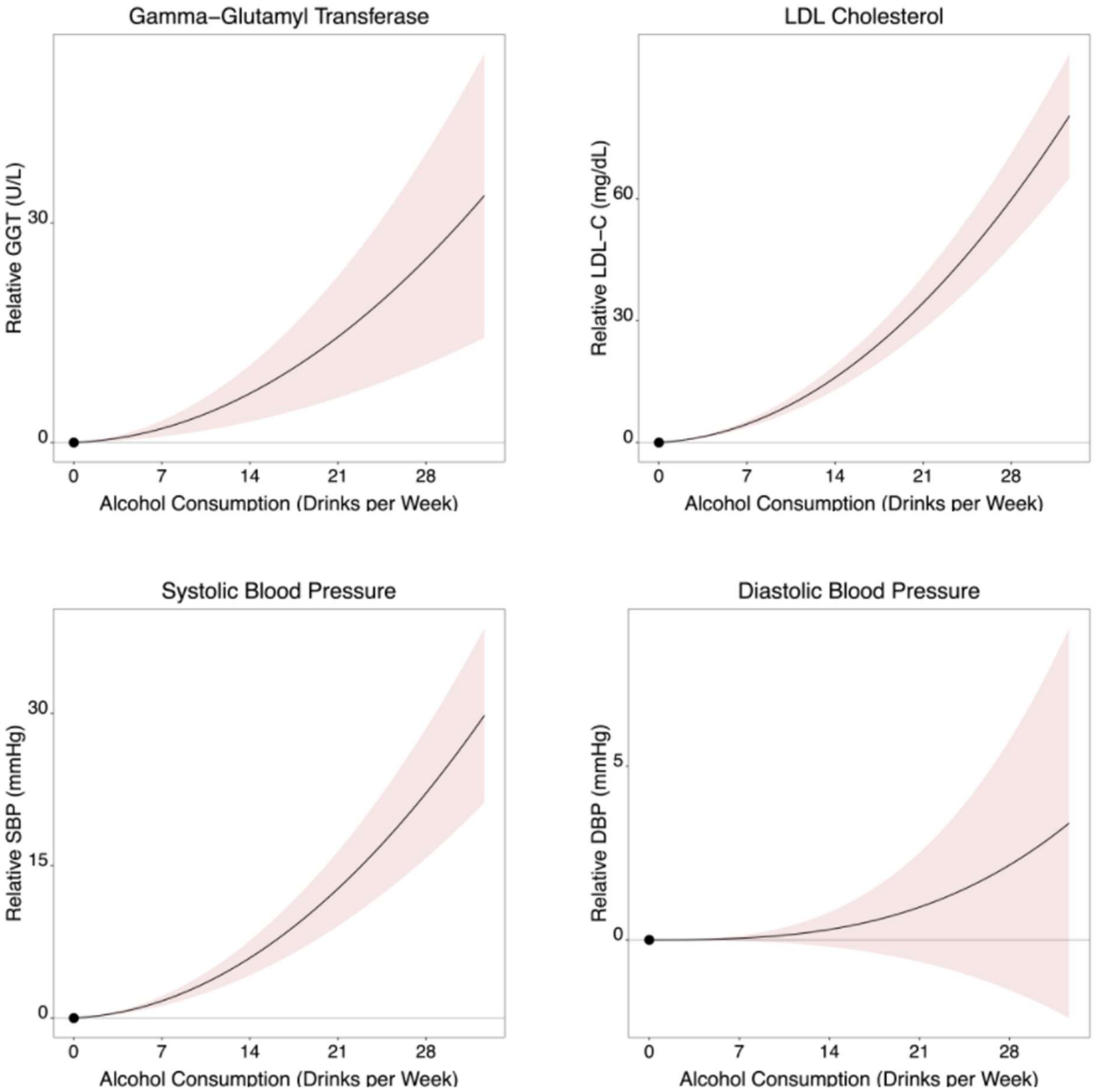

(VIII) Fractional polynomial non-linear MR analyses for six secondary continuous traits using rs1229984 from the biologically relevant *ADH1B* genetic instruments.

© 2022 Biddinger KJ et al. *JAMA Network Open.*

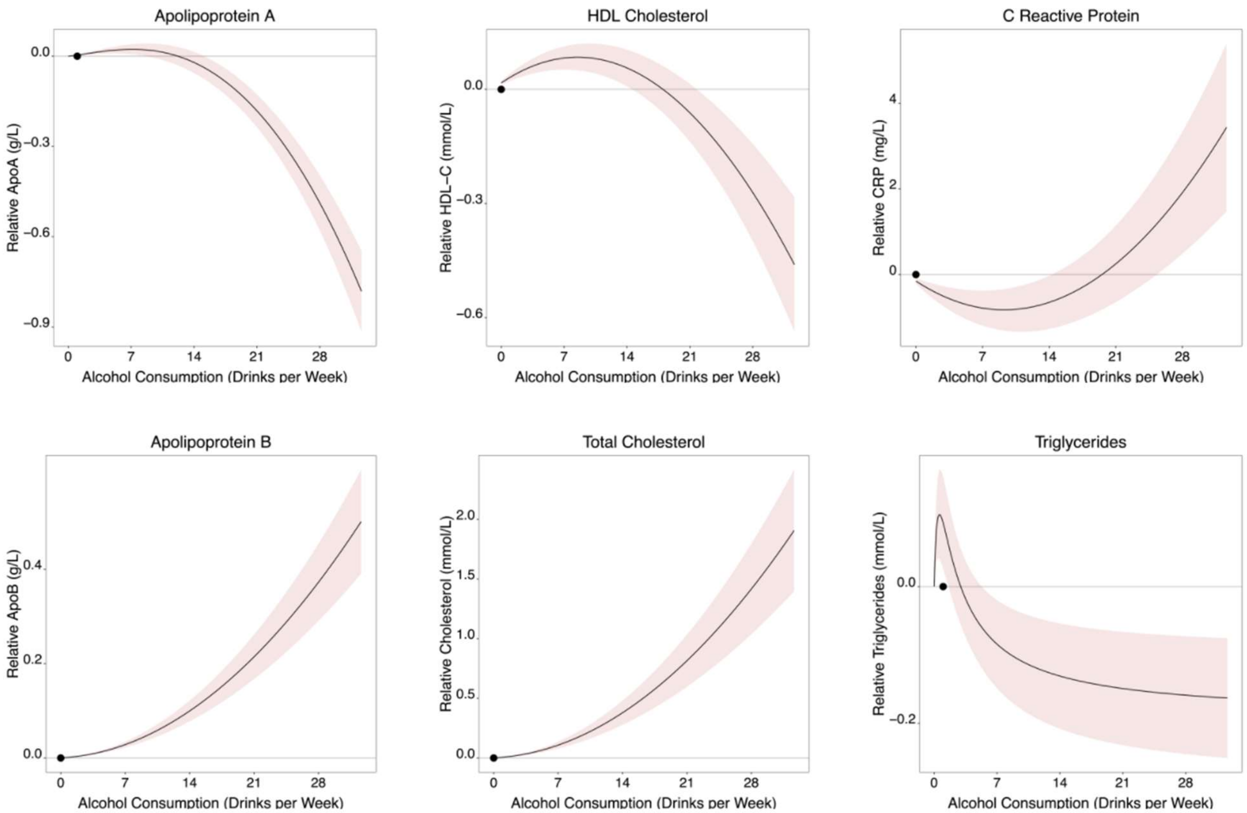

© 2022 Biddinger KJ et al. *JAMA Network Open.*

eFigure 11. Piecewise Nonlinear MR Analyses of Alcohol and Continuous Traits

(I) Piecewise non-linear MR analyses for four continuous traits, all individuals. Gradient at each value of weekly alcohol consumption is the localized average causal effect, calculated using the AUD-R score, for the corresponding category of residual alcohol. Error bars refer to the 95% confidence interval.

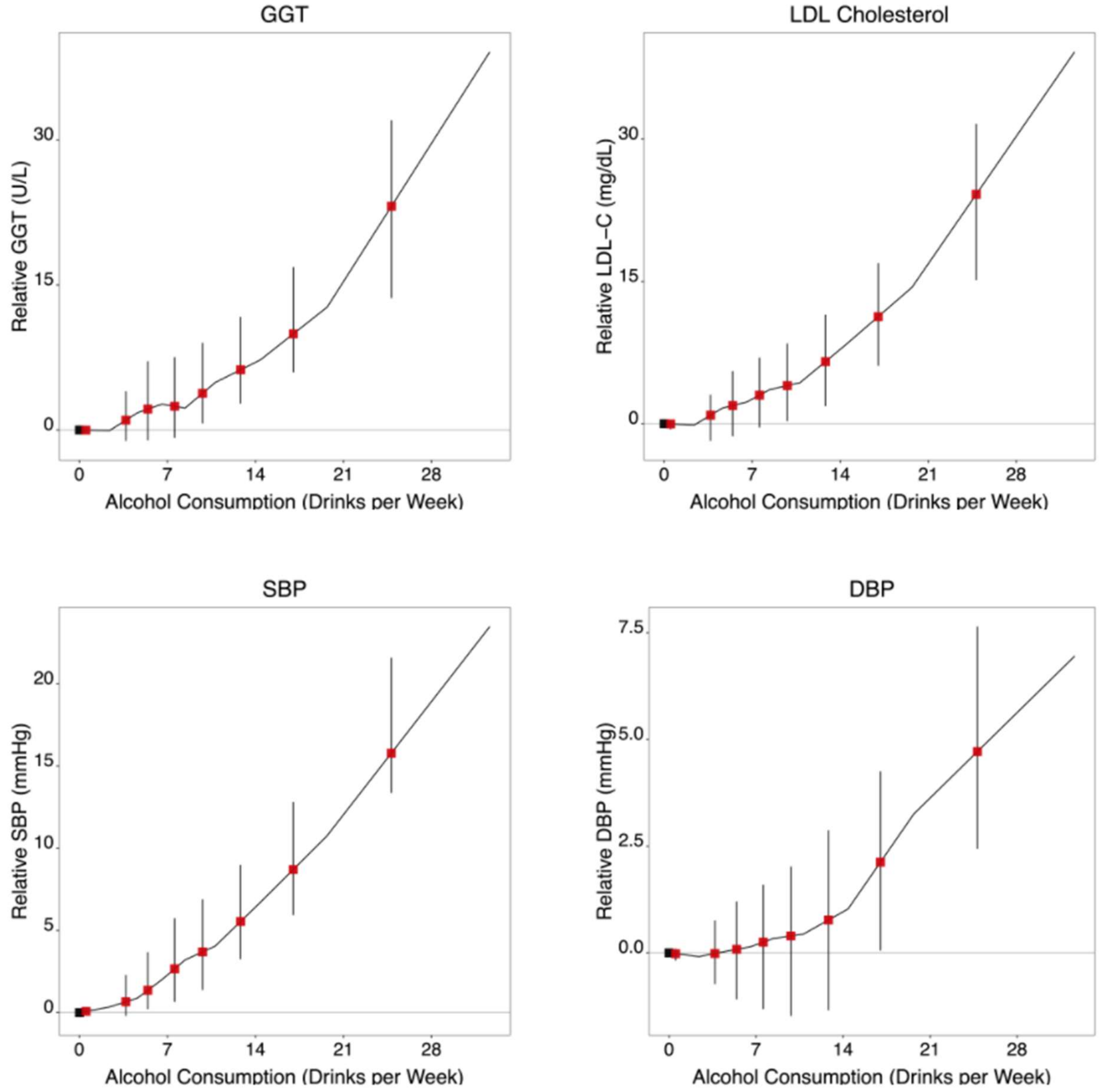

(II) Piecewise non-linear MR analyses for four continuous traits, excluding abstainers. Gradient at each value of weekly alcohol consumption is the localized average causal effect, calculated using the AUD-R score, for the corresponding category of residual alcohol. Error bars refer to the 95% confidence interval.

© 2022 Biddinger KJ et al. *JAMA Network Open.*

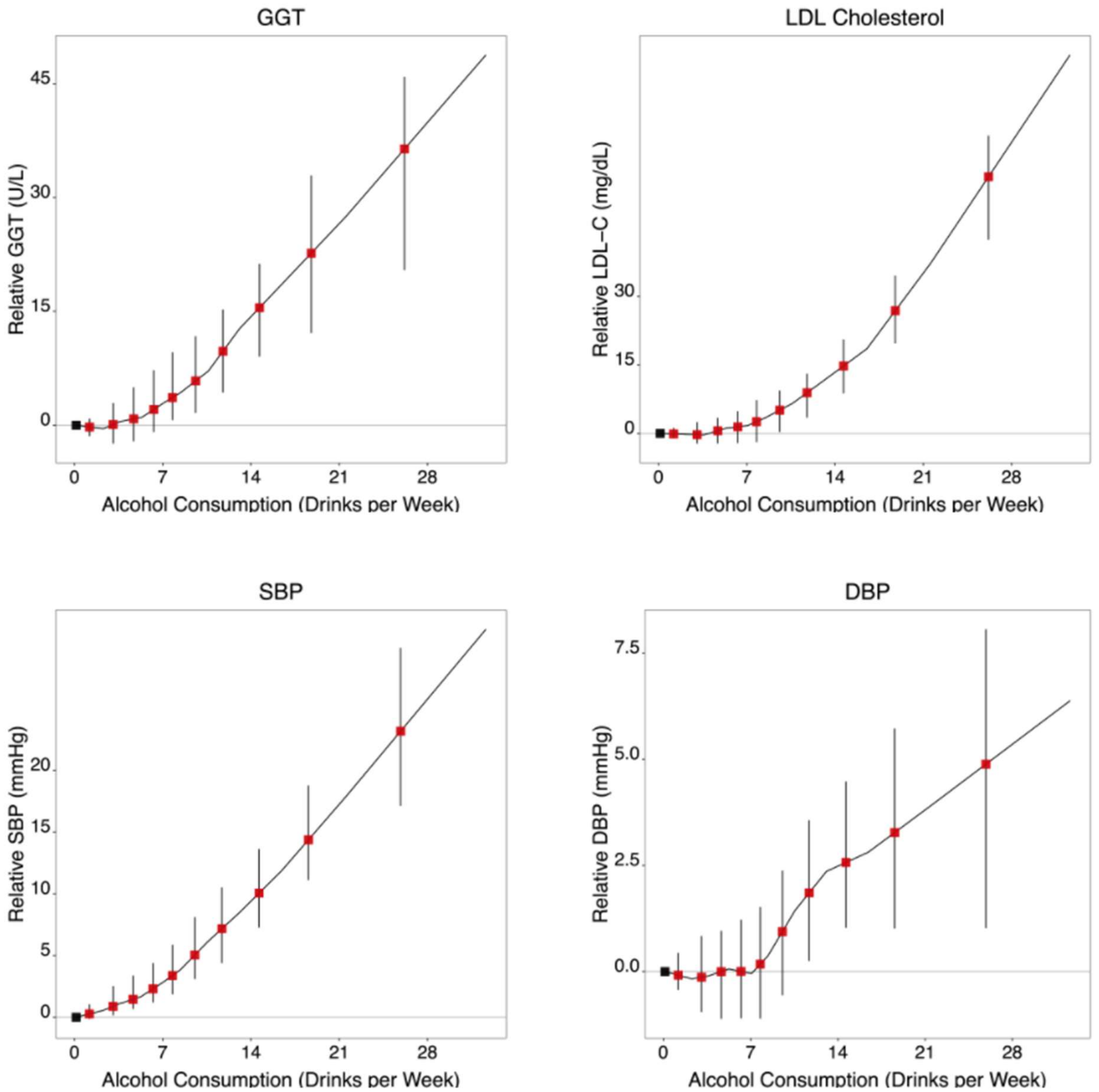

(III) Piecewise non-linear MR analyses for six secondary continuous traits, all individuals. Gradient at each value of weekly alcohol consumption is the localized average causal effect, calculated using the AUD-R score, for the corresponding category of residual alcohol. Error bars refer to the 95% confidence interval.

© 2022 Biddinger KJ et al. *JAMA Network Open.*

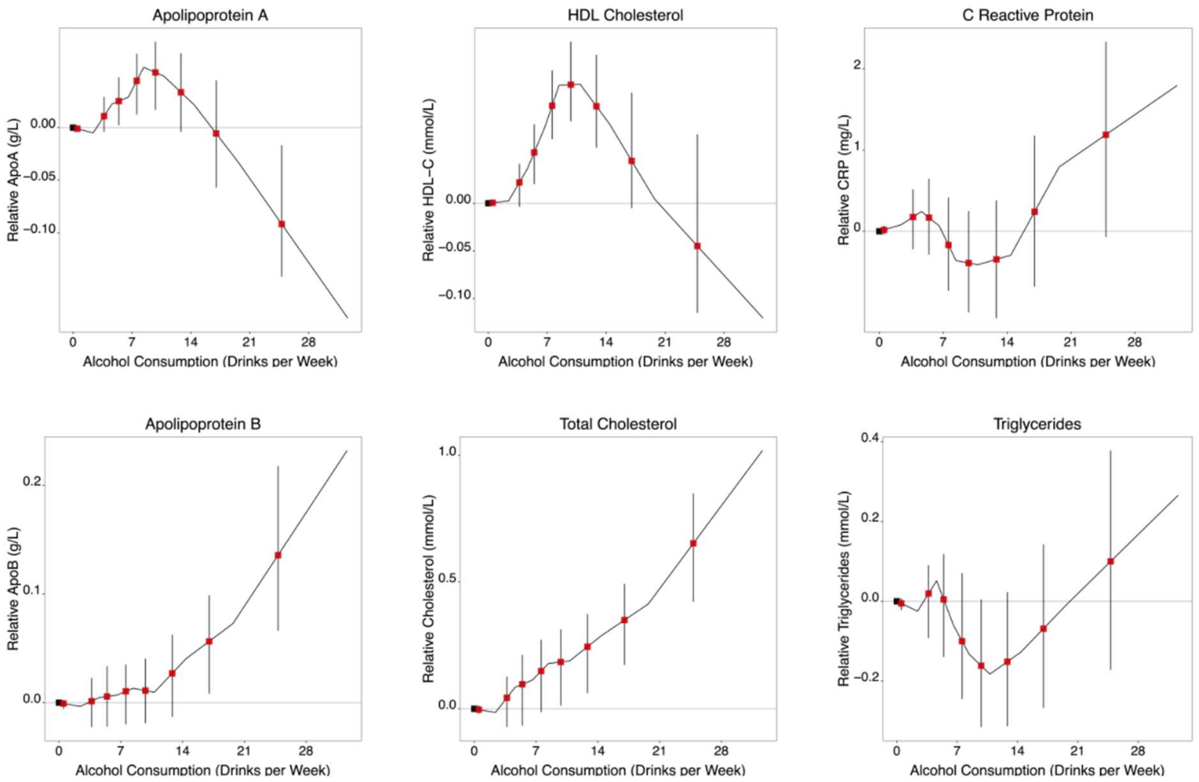

(IV) Piecewise non-linear MR analyses for six secondary continuous traits, excluding abstainers. Gradient at each value of weekly alcohol consumption is the localized average causal effect, calculated using the AUD-R score, for the corresponding category of residual alcohol. Error bars refer to the 95% confidence interval.

© 2022 Biddinger KJ et al. *JAMA Network Open.*

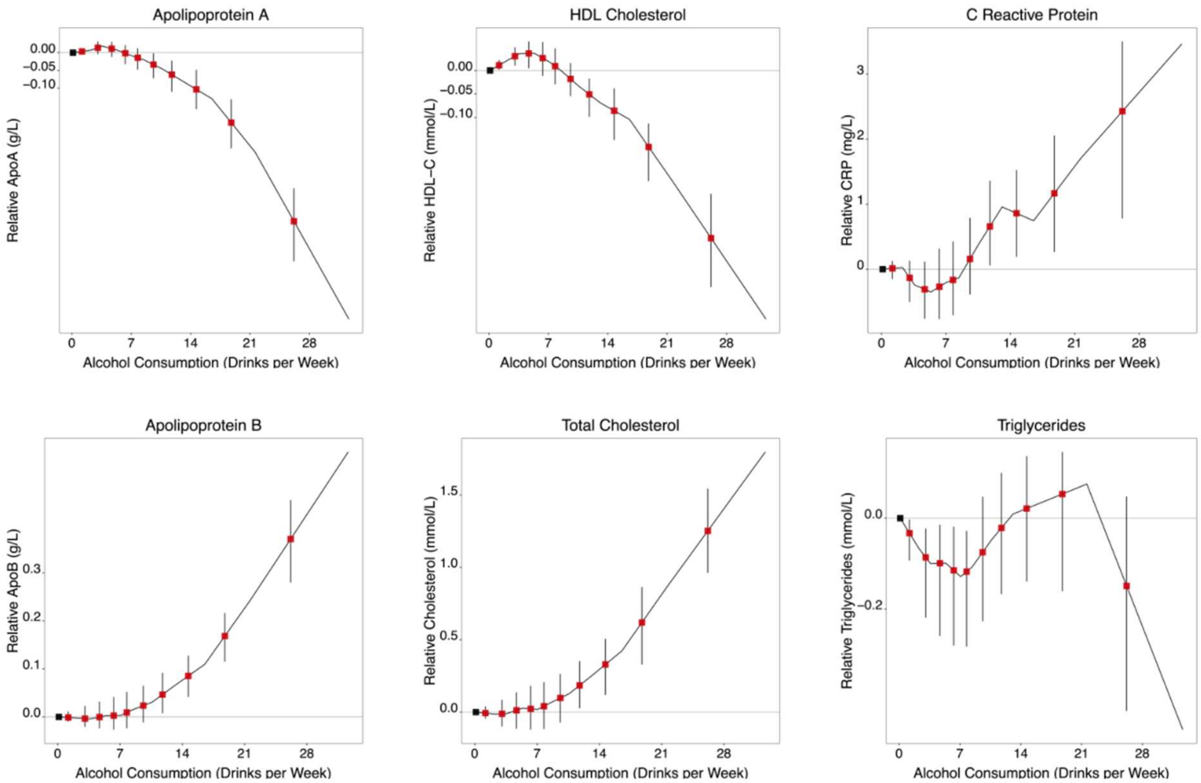

(V) Piecewise non-linear MR analyses for for four primary continuous traits, all individuals. Gradient at each value of weekly alcohol consumption is the localized average causal effect, calculated using the AUDIT-C-R score, for the corresponding category of residual alcohol. Error bars refer to the 95% confidence interval.

© 2022 Biddinger KJ et al. *JAMA Network Open.*

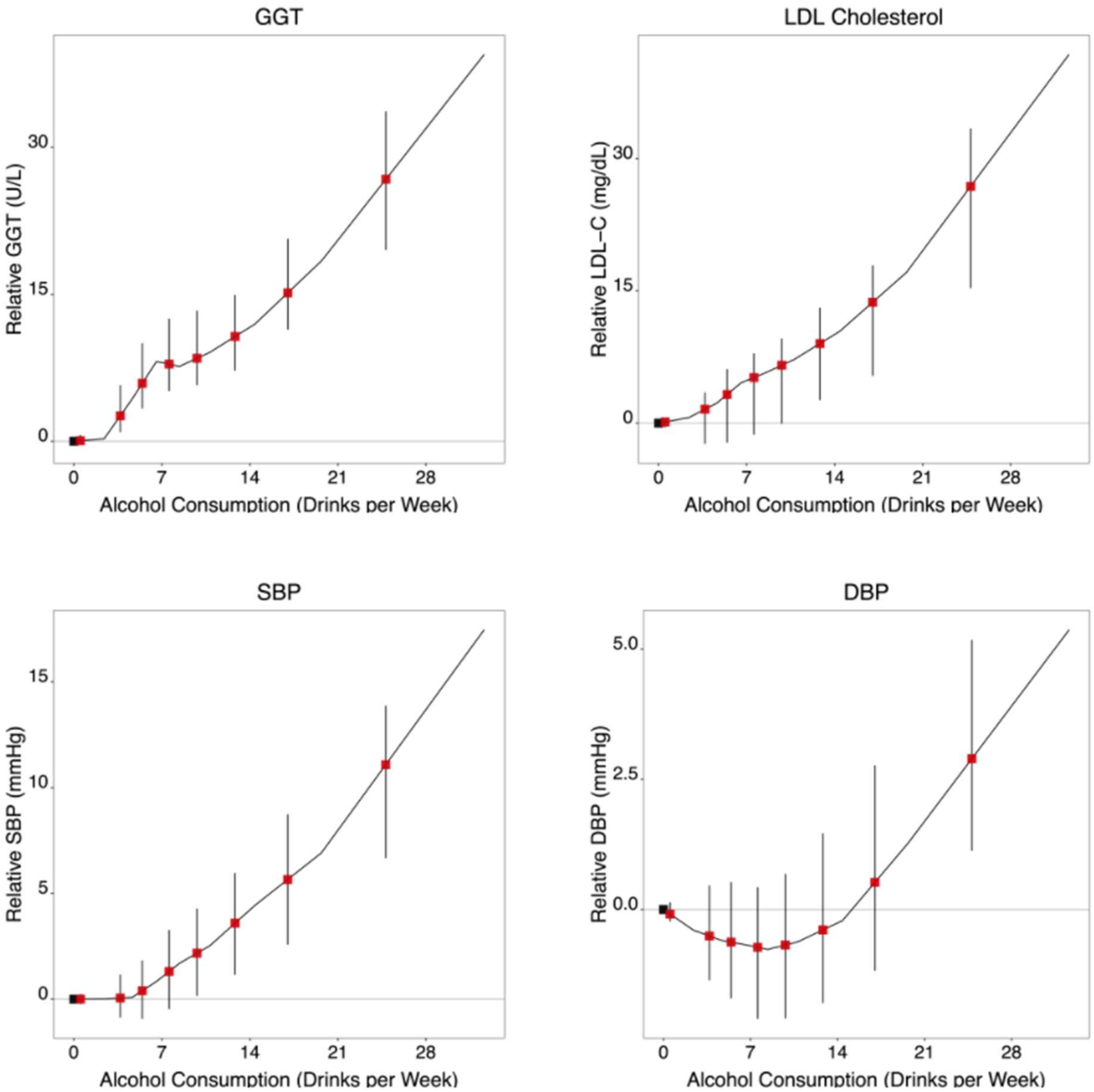

(VI) Piecewise non-linear MR analyses for four primary continuous traits, excluding abstainers. Gradient at each value of weekly alcohol consumption is the localized average causal effect, calculated using the AUDIT-C-R score, for the corresponding category of residual alcohol. Error bars refer to the 95% confidence interval.

© 2022 Biddinger KJ et al. *JAMA Network Open.*

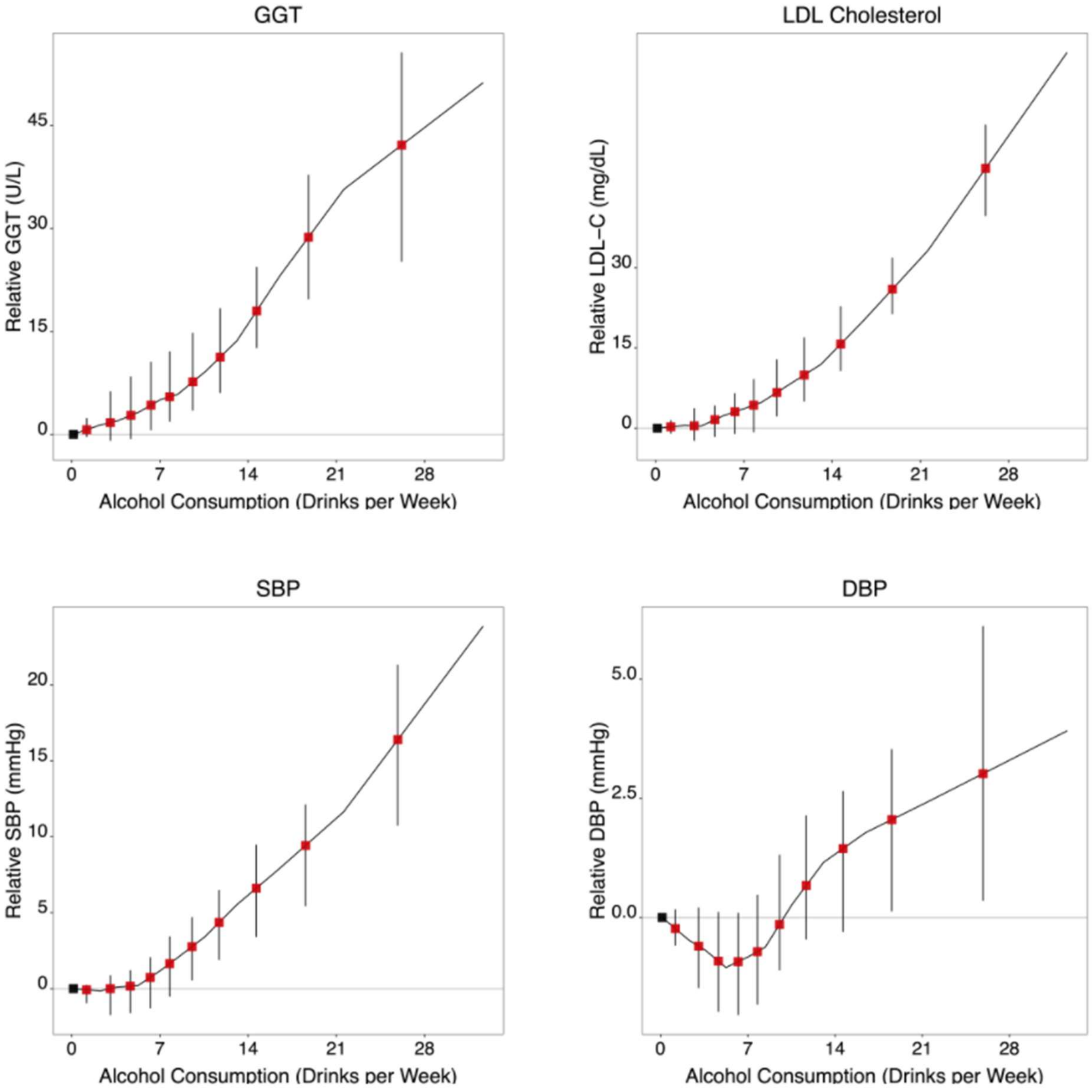

(VII) Piecewise non-linear MR analyses for six secondary continuous traits, all individuals. Gradient at each value of weekly alcohol consumption is the localized average causal effect, calculated using the AUDIT-C-R score, for the corresponding category of residual alcohol. Error bars refer to the 95% confidence interval.

© 2022 Biddinger KJ et al. *JAMA Network Open.*

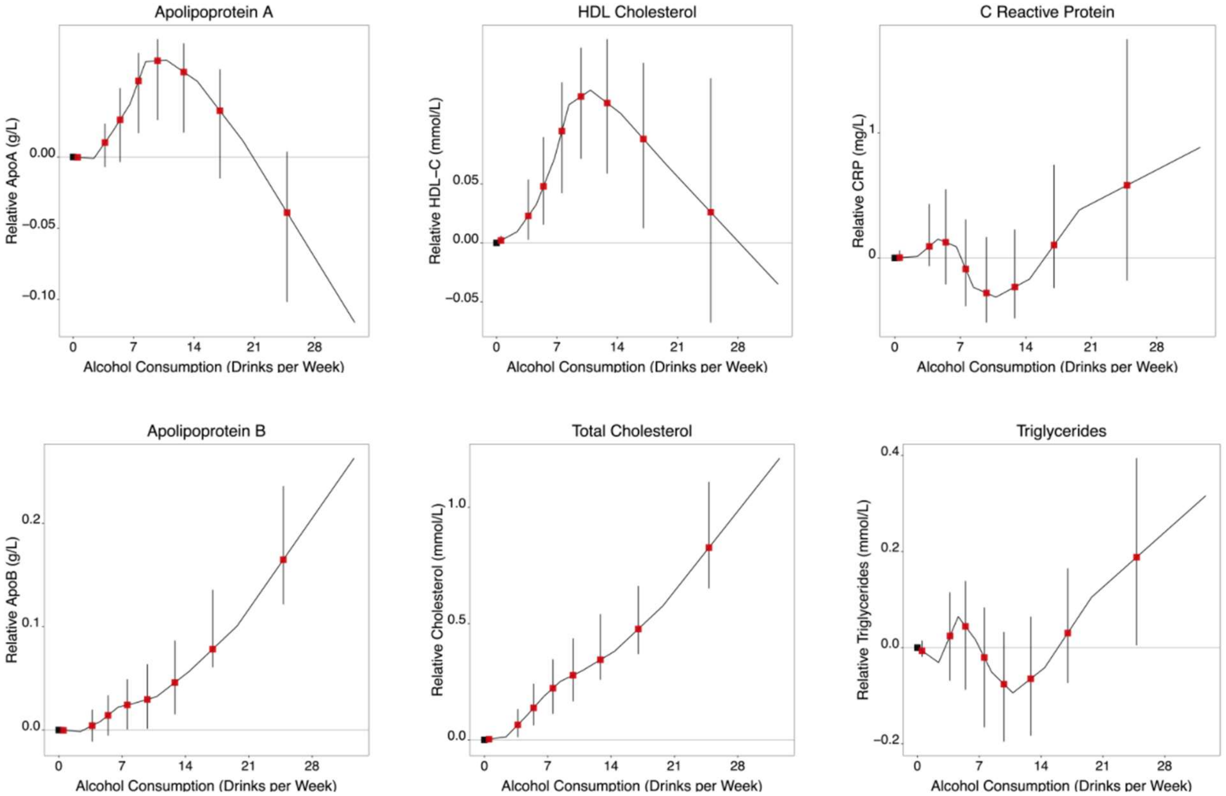

(VIII) Piecewise non-linear MR analyses for six secondary continuous traits, excluding abstainers. Gradient at each value of weekly alcohol consumption is the localized average causal effect, calculated using the AUDIT-C-R score, for the corresponding category of residual alcohol. Error bars refer to the 95% confidence interval.

© 2022 Biddinger KJ et al. *JAMA Network Open.*

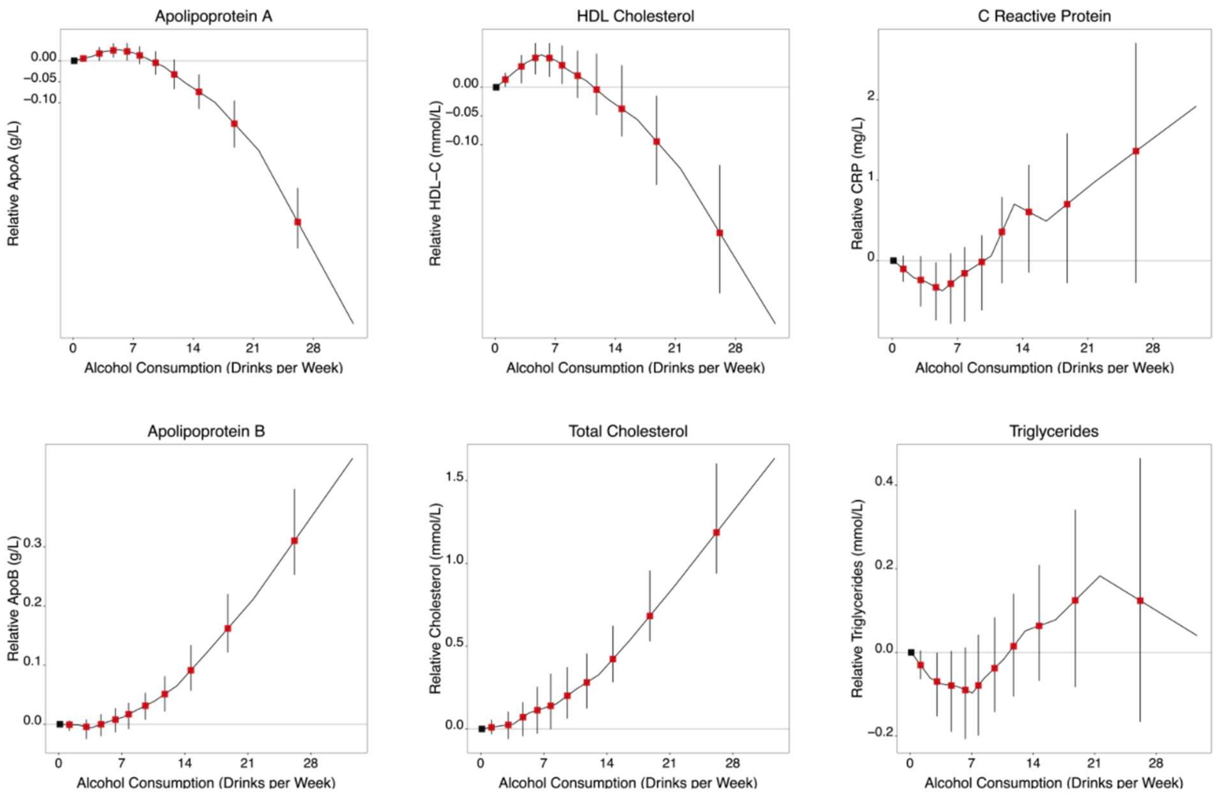

© 2022 Biddinger KJ et al. *JAMA Network Open.*

eFigure 12. Sex-Stratified Nonlinear MR Analyses for Primary Outcomes

Non-linear MR analyses were conducted using fractional polynomial methodology. LACE values were determined in men or women using the AUD-R genetic score. Shaded areas and error bars refer to the 95% confidence interval.

(I) Disease phenotypes, all individuals

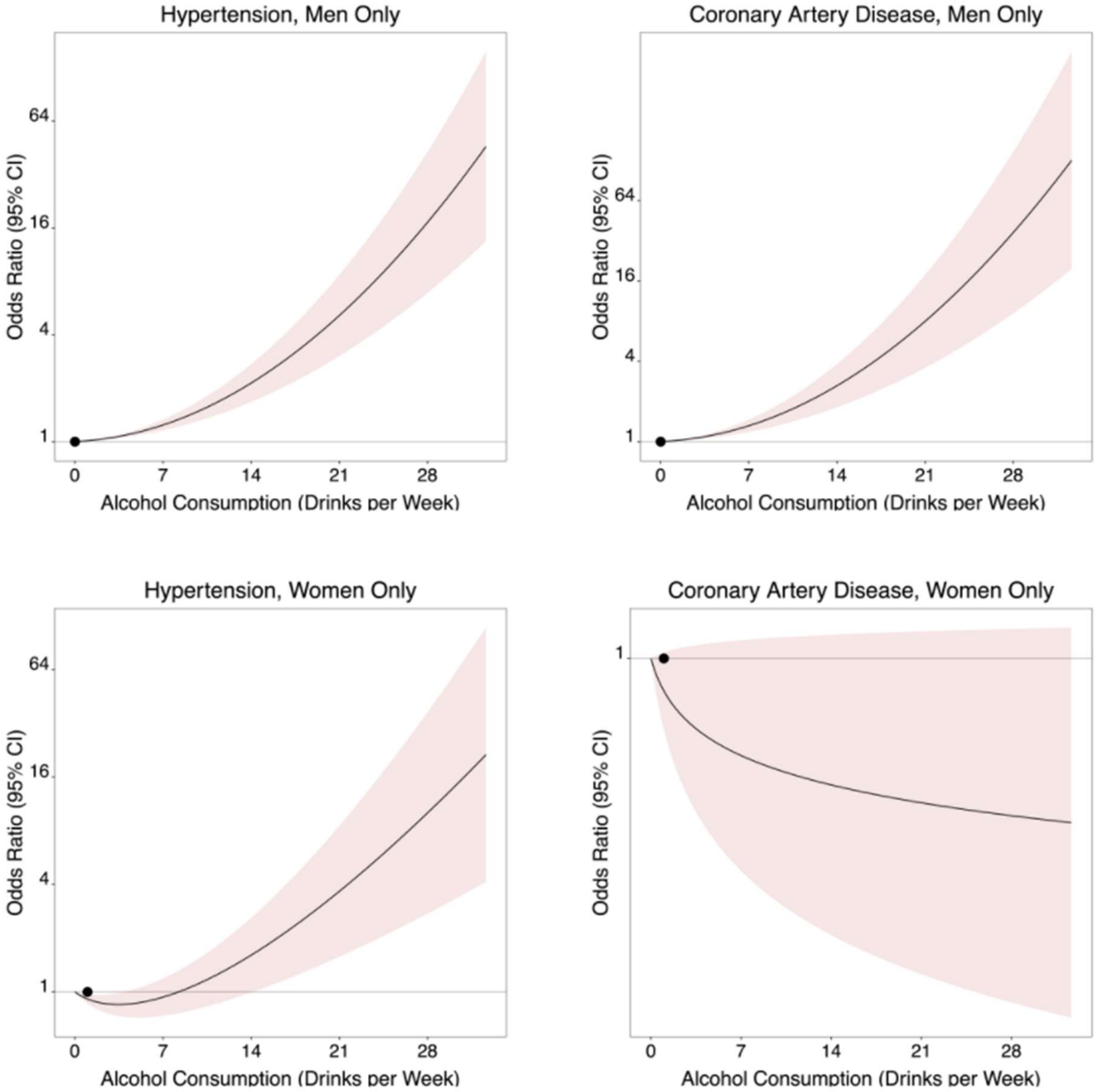

© 2022 Biddinger KJ et al. *JAMA Network Open.*

(II) Disease phenotypes, excluding abstainers

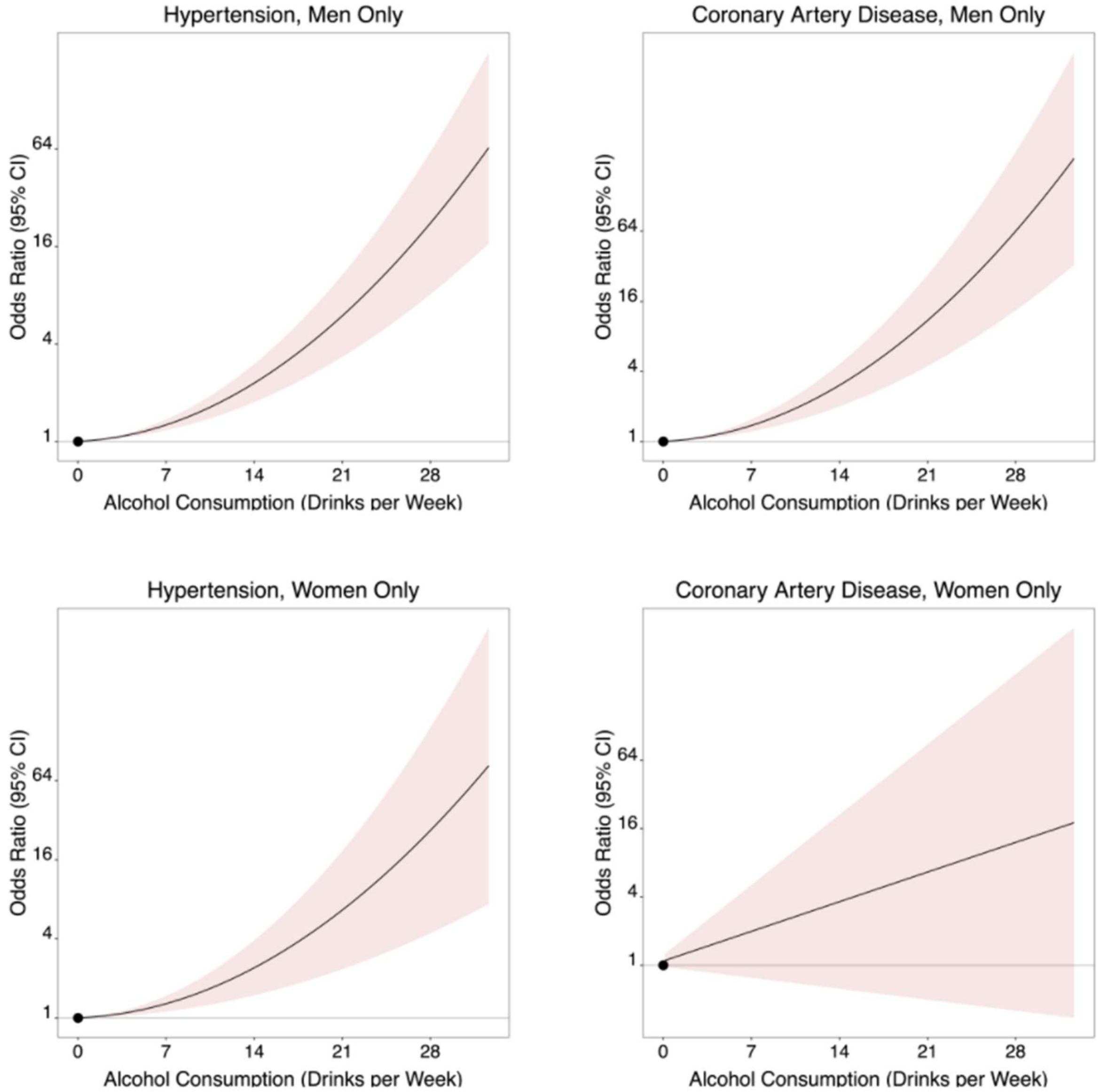

© 2022 Biddinger KJ et al. *JAMA Network Open.*

(III) Continuous outcomes, all individuals

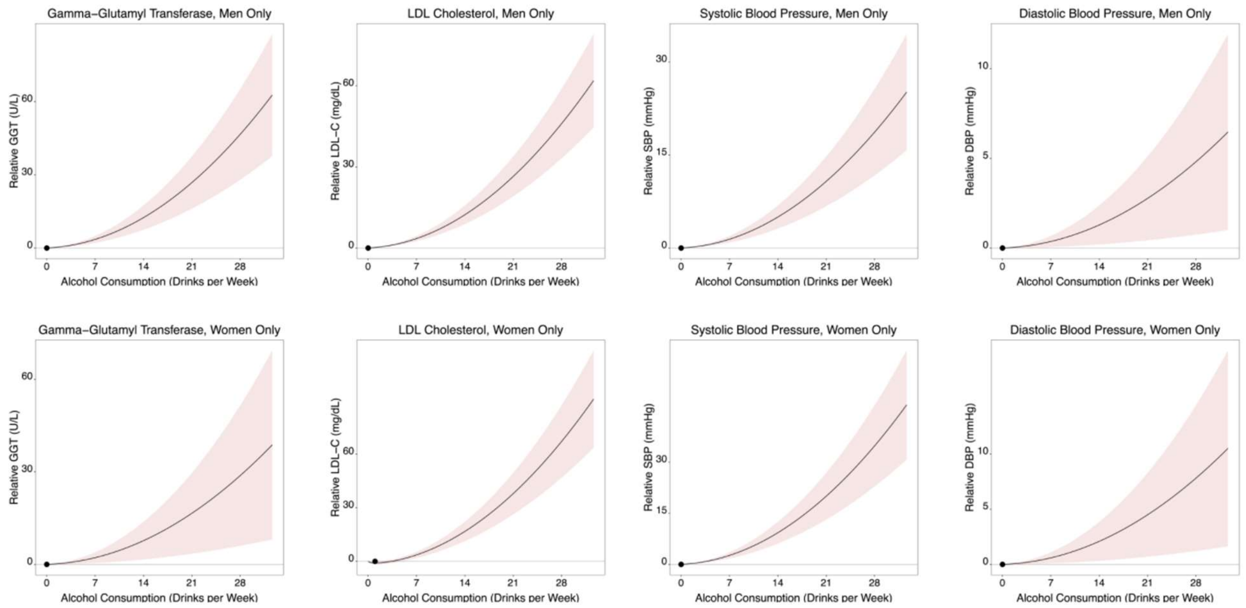

(IV) Continuous outcomes, excluding abstainers

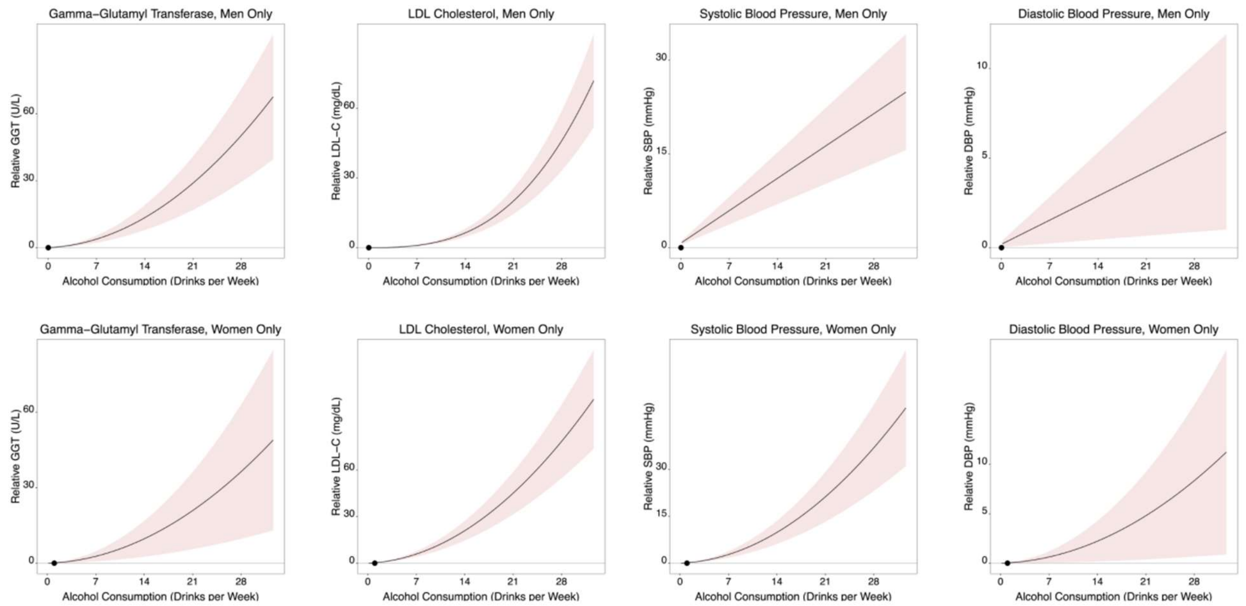

© 2022 Biddinger KJ et al. *JAMA Network Open.*

eFigure 13. Nonlinear MR Analyses of Alcohol and Medication-Corrected Blood Pressure

(I) AUD-R instrument, fractional polynomial analysis

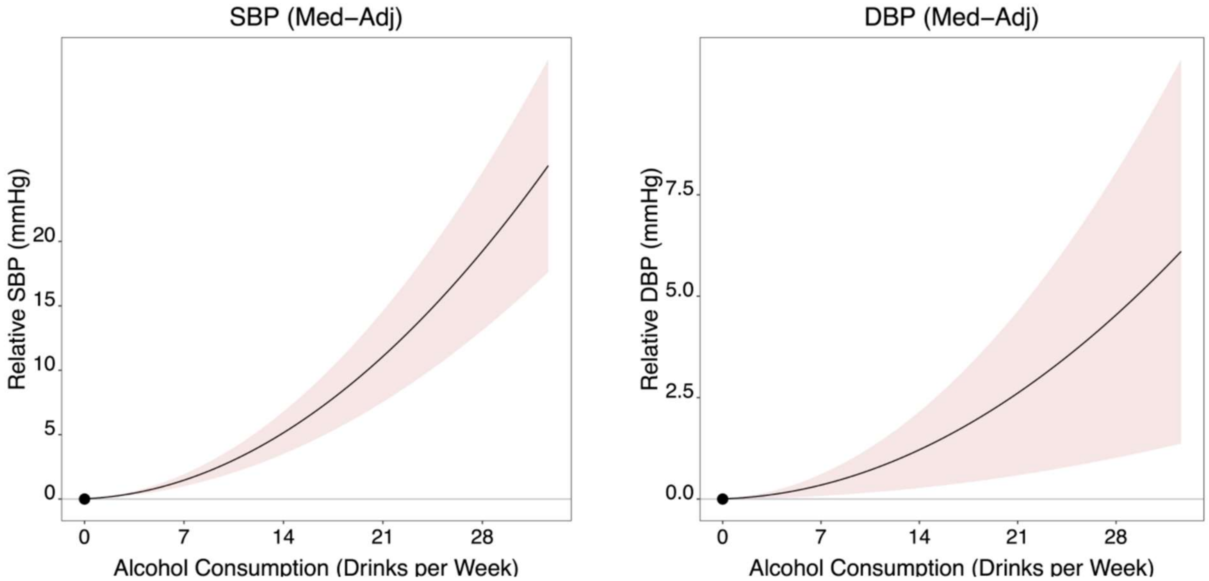

(II) AUD-R instrument, piecewise linear analysis

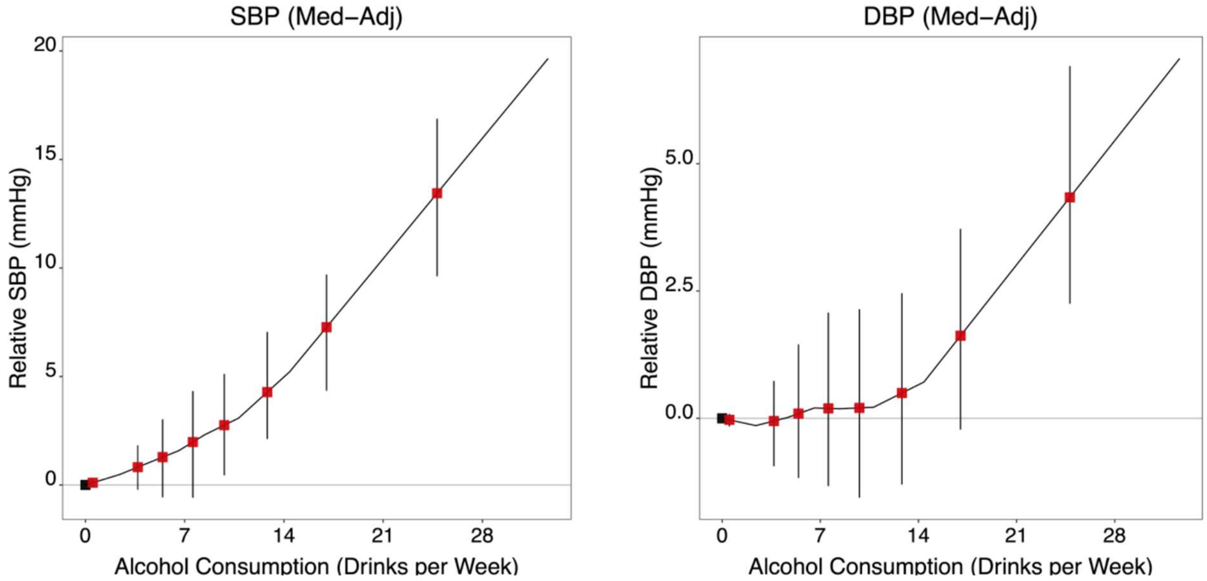

© 2022 Biddinger KJ et al. *JAMA Network Open.*

(III) AUDIT-C-R instrument, fractional polynomial analysis

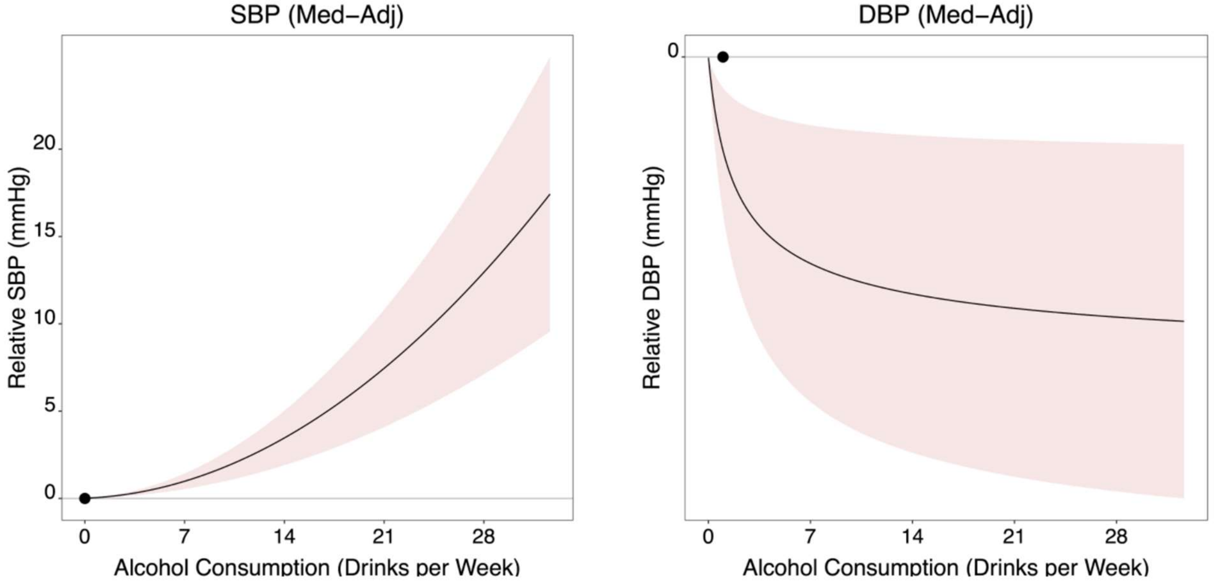

(IV) AUDIT-C-R instrument, piecewise linear analysis

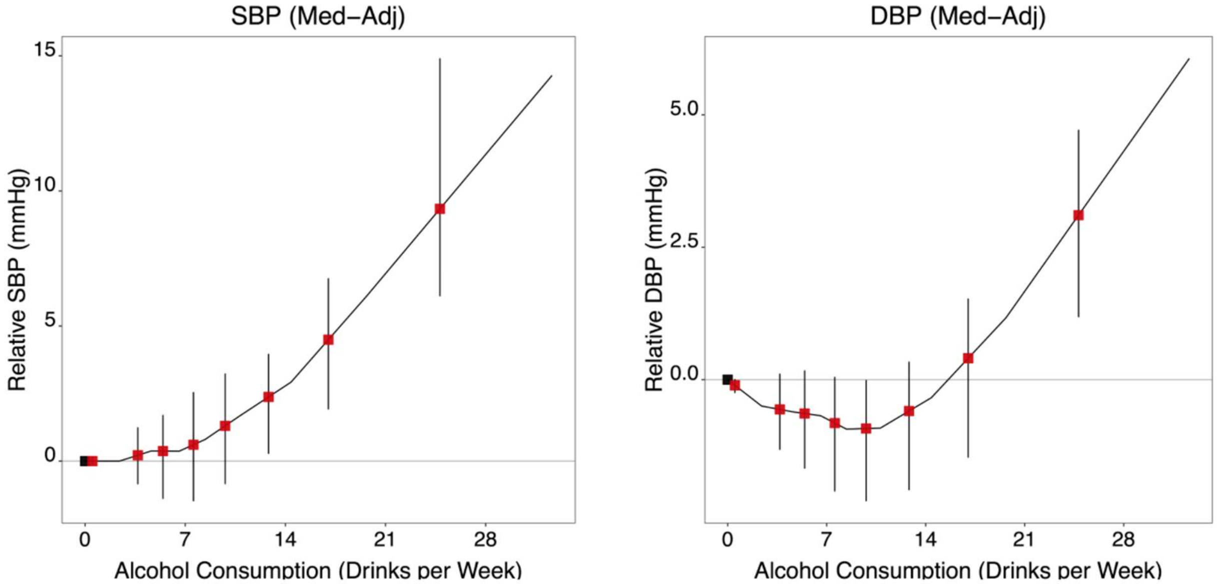

© 2022 Biddinger KJ et al. *JAMA Network Open.*

eFigure 14. Multivariable Fractional Polynomial Nonlinear MR Analyses, Adjusting for Smoking, BMI, and Depression

LACE values – assessed using the primary AUD-R score – were meta-regressed against mean consumption in each strata of alcohol intake, and these plots were reconstructed as the derivative of the best fit model. Shaded areas denote 95% confidence intervals for the model.

(I) Disease outcomes

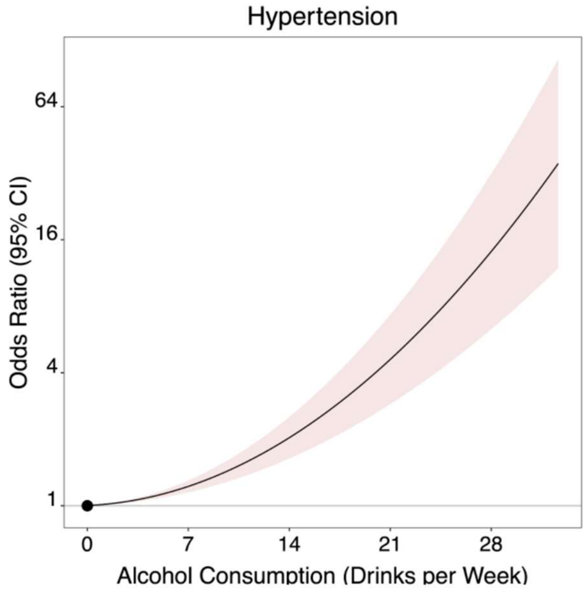

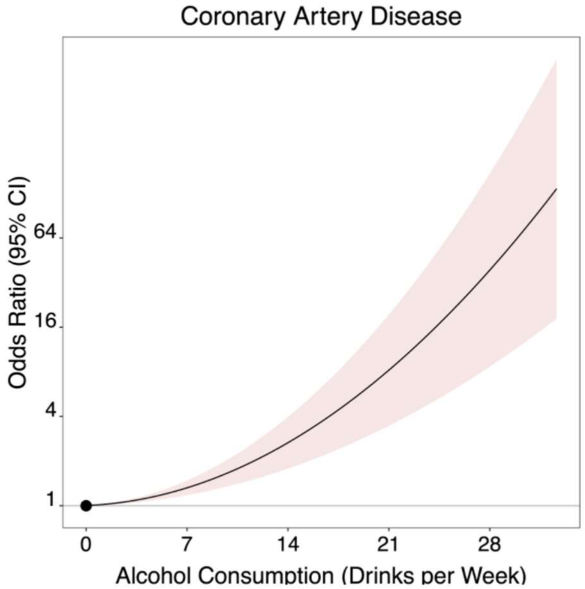

© 2022 Biddinger KJ et al. *JAMA Network Open.*

(II) Continuous traits

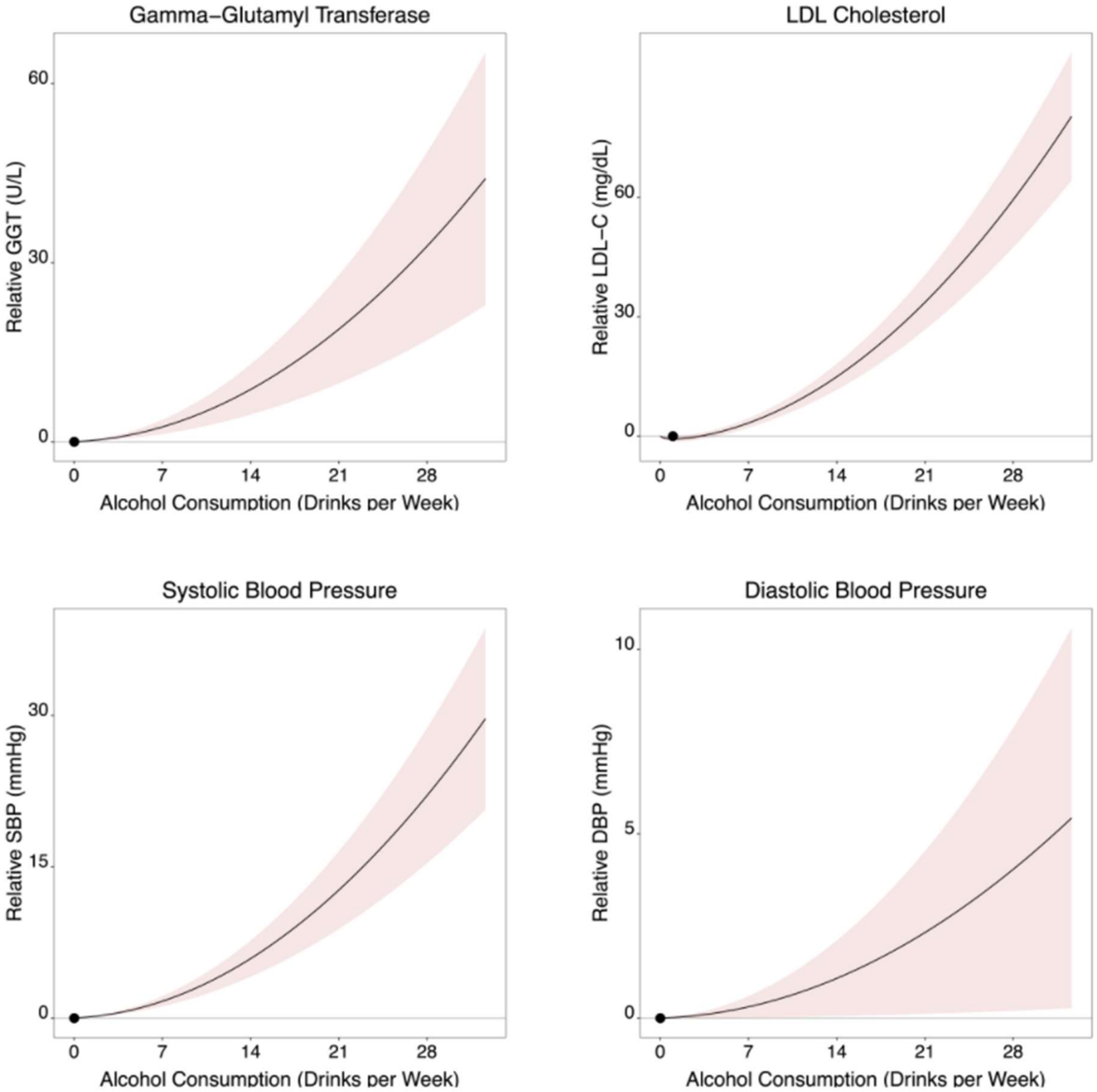

© 2022 Biddinger KJ et al. *JAMA Network Open.*

eFigure 15. Fractional Polynomial Nonlinear MR Analyses of Alcohol and Blood Pressure in Mass General Brigham Biobank

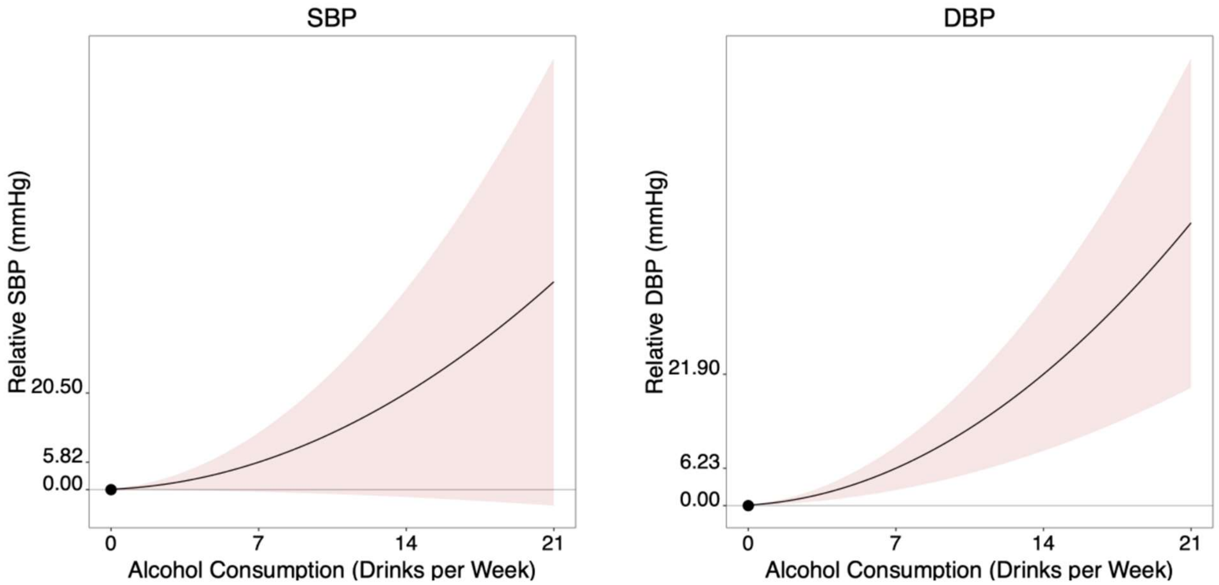

© 2022 Biddinger KJ et al. *JAMA Network Open.*

eReferences

1. About UK Biobank | UK Biobank. Accessed June 9, 2020. https://www.ukbiobank.ac.uk/about-biobank-uk/
2. Bycroft C, Freeman C, Petkova D, et al. The UK Biobank resource with deep phenotyping and genomic data. Nature. 2018;562(7726):203-209. doi:10.1038/s41586-018-0579-z
3. Alcohol units. nhs.uk. Published April 26, 2018. Accessed June 9, 2020. https://www.nhs.uk/live-well/alcohol-support/calculating-alcohol-units/
4. What Is Fortified Wine? Types, Benefits, and Downsides. Accessed June 9, 2020. https://www.healthline.com/nutrition/fortified-wine#types
5. Drinking Levels Defined. National Institute on Alcohol Abuse and Alcoholism (NIAAA). Published September 14, 2011. Accessed June 9, 2020. https://www.niaaa.nih.gov/alcohol-health/overview-alcohol-consumption/moderate-binge-drinking
6. Abel EL, Kruger ML, Friedl J. How do physicians define "light," "moderate," and "heavy" drinking? Alcohol Clin Exp Res. 1998;22(5):979-984. doi:10.1111/j.1530-0277.1998.tb03692.x
7. Aragam KG, Dobbyn A, Judy R, et al. Limitations of Contemporary Guidelines for Managing Patients at High Genetic Risk of Coronary Artery Disease. Journal of the American College of Cardiology. 2020;75(22):2769-2780. doi:10.1016/j.jacc.2020.04.027
8. Kranzler HR, Zhou H, Kember RL, et al. Genome-wide association study of alcohol consumption and use disorder in 274,424 individuals from multiple populations. Nature Communications. 2019;10(1):1499. doi:10.1038/s41467-019-09480-8
9. Instrument: AUDIT-C Questionnaire | NIDA CTN Common Data Elements. Accessed June 9, 2020. https://cde.drugabuse.gov/instrument/f229c68a-67ce-9a58-e040-bb89ad432be4
10. Price AL, Patterson NJ, Plenge RM, Weinblatt ME, Shadick NA, Reich D. Principal components analysis corrects for stratification in genome-wide association studies. Nature Genetics. 2006;38(8):904-909. doi:10.1038/ng1847
11. Burgess S, Thompson SG. Interpreting findings from Mendelian randomization using the MR-Egger method. Eur J Epidemiol. 2017;32(5):377-389. doi:10.1007/s10654-017-0255-x
12. Lloyd-Jones Donald M., Hong Yuling, Labarthe Darwin, et al. Defining and Setting National Goals for Cardiovascular Health Promotion and Disease Reduction. Circulation. 2010;121(4):586-613. doi:10.1161/CIRCULATIONAHA.109.192703
13. Khera AV, Emdin CA, Drake I, et al. Genetic Risk, Adherence to a Healthy Lifestyle, and Coronary Disease. New England Journal of Medicine. 2016;375(24):2349-2358. doi:10.1056/NEJMoa1605086
14. Staley JR, Burgess S. Semiparametric methods for estimation of a nonlinear exposure-outcome relationship using instrumental variables with application to Mendelian randomization. Genet Epidemiol. 2017;41(4):341-352. doi:10.1002/gepi.22041
15. Arvanitis Marios, Qi Guanghao, Bhatt Deepak L., et al. A Linear and Non-Linear Mendelian Randomization Analysis of the Association Between Diastolic Blood Pressure and Cardiovascular Events: The J Curve Revisited. Circulation. 0(0). doi:10.1161/CIRCULATIONAHA.120.049819
16. Millwood IY, Walters RG, Mei XW, et al. Conventional and genetic evidence on alcohol and vascular disease aetiology: a prospective study of 500 000 men and women in China. The Lancet. 2019;393(10183):1831-1842. doi:10.1016/S0140-6736(18)31772-0
17. Sun Y-Q, Burgess S, Staley JR, et al. Body mass index and all cause mortality in HUNT and UK Biobank studies: linear and non-linear mendelian randomisation analyses. BMJ. 2019;364. doi:10.1136/bmj.l1042
18. Royston P, Altman DG. Regression Using Fractional Polynomials of Continuous Covariates: Parsimonious Parametric Modelling. Journal of the Royal Statistical Society: Series C (Applied Statistics). 1994;43(3):429-453. doi:10.2307/2986270
19. Wood AM, Kaptoge S, Butterworth AS, et al. Risk thresholds for alcohol consumption: combined analysis of individual-participant data for 599 912 current drinkers in 83 prospective studies. The Lancet. 2018;391(10129):1513-1523. doi:10.1016/S0140-6736(18)30134-X
20. International Consortium for Blood Pressure Genome-Wide Association Studies, Ehret GB, Munroe PB, et al. Genetic variants in novel pathways influence blood pressure and cardiovascular disease risk. Nature. 2011;478(7367):103-109. doi:10.1038/nature10405
21. True WR, Xian H, Scherrer JF, et al. Common Genetic Vulnerability for Nicotine and Alcohol Dependence in Men. Arch Gen Psychiatry. 1999;56(7):655. doi:10.1001/archpsyc.56.7.655
22. Wills AG, Evans LM, Hopfer C. Phenotypic and genetic relationship between BMI and drinking in a sample of UK adults. Behav Genet. 2017;47(3):290-297. doi:10.1007/s10519-017-9838-2

23. Burgess S, Malik R, Liu B, et al. Dose–response relationship between genetically proxied average blood glucose levels and incident coronary heart disease in individuals without diabetes mellitus. Diabetologia. 2021;64(4):845-849. doi:10.1007/s00125-020-05377-0
24. Locke AE, Kahali B, Berndt SI, et al. Genetic studies of body mass index yield new insights for obesity biology. Nature. 2015;518(7538):197-206. doi:10.1038/nature14177
25. Xu K, Li B, McGinnis KA, et al. Genome-wide association study of smoking trajectory and meta-analysis of smoking status in 842,000 individuals. Nature Communications. 2020;11(1):5302. doi:10.1038/s41467-020-18489-3
26. Levey DF, Stein MB, Wendt FR, et al. Bi-ancestral depression GWAS in the Million Veteran Program and meta-analysis in >1.2 million individuals highlight new therapeutic directions. Nat Neurosci. 2021;24(7):954-963. doi:10.1038/s41593-021-00860-2

© 2022 Biddinger KJ et al. *JAMA Network Open.*
